# A common allele of *HLA* is associated with asymptomatic SARS-CoV-2 infection

**DOI:** 10.1038/s41586-023-06331-x

**Published:** 2023-07-19

**Authors:** Danillo G. Augusto, Lawton D. Murdolo, Demetra S. M. Chatzileontiadou, Joseph J. Sabatino, Tasneem Yusufali, Noah D. Peyser, Xochitl Butcher, Kerry Kizer, Karoline Guthrie, Victoria W. Murray, Vivian Pae, Sannidhi Sarvadhavabhatla, Fiona Beltran, Gurjot S. Gill, Kara L. Lynch, Cassandra Yun, Colin T. Maguire, Michael J. Peluso, Rebecca Hoh, Timothy J. Henrich, Steven G. Deeks, Michelle Davidson, Scott Lu, Sarah A. Goldberg, J. Daniel Kelly, Jeffrey N. Martin, Cynthia A. Vierra-Green, Stephen R. Spellman, David J. Langton, Michael J. Dewar-Oldis, Corey Smith, Peter J. Barnard, Sulggi Lee, Gregory M. Marcus, Jeffrey E. Olgin, Mark J. Pletcher, Martin Maiers, Stephanie Gras, Jill A. Hollenbach

**Affiliations:** 1 Weill Institute for Neurosciences, Department of Neurology, University of California, San Francisco, CA USA; 2 Department of Biological Sciences, The University of North Carolina at Charlotte, Charlotte, NC USA; 3 Programa de Pós-Graduação em Genética, Universidade Federal do Paraná, Curitiba, Brazil; 4 Department of Biochemistry and Chemistry, La Trobe Institute for Molecular Science, La Trobe University, Bundoora, Victoria Australia; 5 Department of Biochemistry and Molecular Biology, Biomedicine Discovery Institute, Monash University, Clayton, Victoria Australia; 6 Division of Cardiology, Department of Medicine, University of California, San Francisco, CA USA; 7 Division of HIV, Infectious Diseases and Global Medicine, Department of Medicine, University of California, San Francisco, CA USA; 8 Department of Laboratory Medicine, University of California, San Francisco, CA USA; 9 Clinical and Translational Science Institute, University of Utah, Salt Lake City, UT USA; 10 Division of Experimental Medicine, Department of Medicine, University of California, San Francisco, CA USA; 11 Department of Medicine, University of California, San Francisco, CA USA; 12 Department of Epidemiology and Biostatistics, University of California, San Francisco, CA USA; 13 F.I. Proctor Foundation, University of California, San Francisco, CA USA; 14 CIBMTR (Center for International Blood and Marrow Transplant Research), National Marrow Donor Program/Be The Match, Minneapolis, MN USA; 15 ExplantLab, Newcastle-upon-Tyne, UK; 16 QIMR Berghofer Centre for Immunotherapy and Vaccine Development Brisbane, QIMR Berghofer Medical Research Institute, Brisbane, Queensland Australia; 17 Division of General Internal Medicine, University of California, San Francisco, CA USA

**Keywords:** Disease genetics, CD8-positive T cells, Viral infection, SARS-CoV-2, Antigen processing and presentation

## Abstract

Studies have demonstrated that at least 20% of individuals infected with SARS-CoV-2 remain asymptomatic^1–4^. Although most global efforts have focused on severe illness in COVID-19, examining asymptomatic infection provides a unique opportunity to consider early immunological features that promote rapid viral clearance. Here, postulating that variation in the human leukocyte antigen (*HLA*) loci may underly processes mediating asymptomatic infection, we enrolled 29,947 individuals, for whom high-resolution *HLA* genotyping data were available, in a smartphone-based study designed to track COVID-19 symptoms and outcomes. Our discovery cohort (*n* = 1,428) comprised unvaccinated individuals who reported a positive test result for SARS-CoV-2. We tested for association of five *HLA* loci with disease course and identified a strong association between HLA-B*15:01 and asymptomatic infection, observed in two independent cohorts. Suggesting that this genetic association is due to pre-existing T cell immunity, we show that T cells from pre-pandemic samples from individuals carrying HLA-B*15:01 were reactive to the immunodominant SARS-CoV-2 S-derived peptide NQKLIANQF. The majority of the reactive T cells displayed a memory phenotype, were highly polyfunctional and were cross-reactive to a peptide derived from seasonal coronaviruses. The crystal structure of HLA-B*15:01–peptide complexes demonstrates that the peptides NQKLIANQF and NQKLIANAF (from OC43-CoV and HKU1-CoV) share a similar ability to be stabilized and presented by HLA-B*15:01. Finally, we show that the structural similarity of the peptides underpins T cell cross-reactivity of high-affinity public T cell receptors, providing the molecular basis for HLA-B*15:01-mediated pre-existing immunity.

## Abstract (web summary)

The human leukocyte antigen allele HLA-B*15:01 is associated with asymptomatic SARS-CoV-2 infection due to pre-existing T cell immunity.

## Main

Despite some inconsistent reporting of symptoms^1^, studies have shown that at least 20% of individuals infected with severe acute respiratory syndrome coronavirus 2 (SARS-CoV-2) remain asymptomatic^2–4^. The examination of asymptomatic infection provides a unique opportunity to consider early disease and immunological features that promote rapid viral clearance. Specific focus on asymptomatic infection has the potential to further our understanding of disease pathogenesis and supports ongoing efforts towards vaccine development and the identification of potential therapeutic targets.

It remains unclear why many individuals successfully clear infection without major complications while others develop severe disease, even without known risk factors for severe COVID-19 outcomes^5^. However, host genetics is known to be implicated in differential immunological responses to infection and disease progression. Numerous studies intending to understand the genetic basis of differential outcomes in COVID-19 have been underway since nearly the start of the global pandemic, including the multicentre Host Genetics Initiative^6^. However, the vast majority of these studies have examined genetic associations with severe disease course, in primarily hospitalized cohorts^7,8^. As a result, although most individuals infected with SARS-CoV-2 experience mild disease course or are entirely asymptomatic, very few studies have examined genetics in the context of non-hospitalized, prospective, community-based cohorts.

The human leukocyte antigen (*HLA*) region (6p21) is the most polymorphic and medically important human genomic region. Variation in *HLA* has been associated with hundreds of diseases and conditions, including infection. Among the many genes involved in human immune responses, *HLA* variants have among the strongest associations with viral infections. For example, *HLA* is associated with rapid progression and viral load control of human immunodeficiency virus (HIV)^9^, hepatitis B, hepatitis C and other infectious diseases^10^. Notably, *HLA* class I and class II alleles have also been associated with the severe acute respiratory syndrome caused by SARS-CoV^11–13^.

In silico analyses have pointed to HLA as relevant molecules for SARS-CoV-2 risk and essential targets for vaccine development^14–17^. For example, HLA-B*46:01 has low predicted binding to peptides of SARS-CoV-2, suggesting that individuals expressing this molecule may be more vulnerable to COVID-19^16^, corroborating previous results showing HLA-B*46:01 association with SARS risk^12^. By contrast, HLA-B*15:03 was predicted to protect against COVID-19 by presenting highly conserved SARS-CoV-2 peptides to T cells^16^. More recently, it was demonstrated that, although there is some overlap, many SARS-CoV-2 epitopes for CD8^+^ T cells are HLA specific^16^. To date, relatively few studies have directly examined *HLA* associations with infection, with mixed and inconclusive results in relatively small cohorts^18–20^. Larger studies that relied on genome-wide data to impute *HLA* did not find robust associations with disease^7,21^; however, these studies focused primarily on hospitalized patients with a severe disease course.

Understanding the impact of *HLA* variation in disease promises to provide meaningful insights that are relevant to understanding the immunopathogenesis of COVID-19, while informing vaccine development and potential immunotherapies. Here we present a large study directly examining *HLA* variation in the context of primarily mild disease. We invited volunteer bone marrow donors, from whom high-resolution *HLA* genotyping data were already available, to participate in the COVID-19 Citizen Science Study—a smartphone-based study designed to track COVID-19 symptoms and outcomes, including self-reported positive tests for SARS-CoV-2 infection, to develop a prospective cohort currently comprising nearly 30,000 individuals, as well as two additional independent cohorts. We further contextualize our findings by examining T cell reactivity, T cell receptor repertoire, affinity and structural implications for the observed *HLA* associations. Our results provide strong support for the role of HLA class I in viral clearance leading to asymptomatic infection among individuals with SARS-CoV-2 infection and provide an important framework for additional studies aimed at revealing the immunological and genetic basis for recovery from SARS-CoV-2 infection.

## HLA-B*15:01 in asymptomatic COVID-19

Our final cohort comprised 1,428 individuals who reported a positive test for active SARS-CoV-2 infection and self-identified as white. Basic demographics for all individuals are given in Extended Data Table 1. The full list of reported diseases and conditions and their frequency in this cohort is given in Supplementary Table 1. To identify whether *HLA* variation affects the likelihood of an individual remaining asymptomatic after SARS-CoV-2 infection, we analysed high-resolution genotyping data for five highly polymorphic *HLA* class I and class II genes (*HLA-A*, *HLA-B*, *HLA-C*, *HLA-DRB1*, *HLA-DQB1*).

We found that the allele HLA-B*15:01 was significantly overrepresented in asymptomatic individuals relative to symptomatic individuals (frequency = 0.1103 versus 0.0495, odds ratio (OR) = 2.38, 95% confidence interval (CI) = 1.51–3.65, *P* = 3 × 10^−5^, Bonferroni-corrected *P* (*P*_adj_) = 0.002). No other *HLA* allele at any locus was significantly associated after correction for multiple comparisons. Allele frequencies for all loci are provided in Supplementary Table 2.

To adjust for the effect of comorbid conditions, as well as sex and age differences, we fitted a series of regression models but did not find any effect of patient-reported comorbidities on the likelihood of asymptomatic disease. Thus, our final model was adjusted only for age and sex, again showing a significant association of HLA-B*15:01 with asymptomatic infection after adjustment for these variables (OR = 2.40, 95% CI = 1.54–3.64, *P* = 5.67 × 10^−5^, *P*_adj_ = 0.003; Table 1).

Finally, we observed a strong additive effect for the associated genotype. Individuals carrying two copies of HLA-B*15:01 are more than eight times more likely to remain asymptomatic than individuals carrying other genotypes (OR = 8.58, 95% CI = 1.74–34.43, *P* = 0.001). Overall, one in five individuals (20%) who remained asymptomatic after infection carried HLA-B*15:01, compared with 9% among patients reporting symptoms.

## HLA-DRB1*04:01 enhances HLA-B*15:01

To understand whether additional *HLA* alleles might interact with HLA-B*15:01 in asymptomatic infection, we tested all pairwise two-locus haplotypes containing *HLA-B*. Overall, haplotypic associations for *HLA-B~HLA-DRB1* and *HLA-A~HLA-B* were found to be significant at *P* = 0.01. Examining specific allelic haplotypes, these associations were driven by two HLA-B*15:01 haplotypes: HLA-B*15:01~HLA-DRB1*04:01 and HLA-A*02:01~HLA-B*15:01 (Supplementary Tables 3 and 4).

After adjusting for sex and age, only the combination of HLA-B*15:01 and HLA-DRB1*04:01 remained significant after correction for multiple comparisons (*P* = 3 × 10^−4^, *P*_adj_ = 0.01). We found an OR for this combination (OR = 3.17, 95% CI = 1.65–5.80) that exceeds that for HLA-B*15:01 alone, suggesting that, although not significantly associated with the asymptomatic infection on its own in this cohort, the class II allele HLA-DRB1*04:01 enhances the effect of HLA-B*15:01.

## Replication in independent cohorts

To confirm our findings, we examined two independent cohorts of patients with European ancestry. We first undertook a reanalysis of the primary *HLA* genotype data in a UK cohort that was previously reported^22^; HLA-B*15:01 was not examined with respect to asymptomatic infection owing to its marginal significance in their analyses. Testing only the allele of interest, we found that HLA-B*15:01 is strongly associated with asymptomatic infection in this cohort when adjusting for sex and age (*P* = 0.02, OR = 3.56, 95% CI = 1.15–10.94). Similar to our discovery cohort, we found that the carrier frequency for HLA-B*15:01 was 17% in asymptomatic individuals compared with 7% in symptomatic patients (Table 1).

**Table 1:** HLA-B*15:01 is associated with asymptomatic SARS-CoV-2 infection

	Asymptomatic	Symptomatic				
	cf	cf	OR	95% CI	*P*	*P*_adj_
**Discovery cohort**						
HLA-B*15:01	0.199	0.094	2.40	1.54–3.64	5.67 × 10^−5^	0.002
HLA-B*15:01/15:01	0.022	0.005	8.58	1.74–34.43	0.001	
**UK cohort**						
HLA-B*15:01	0.171	0.070	3.56	1.15–10.97	0.02	
**CHIRP/LIINC cohort**						
HLA-B*15:01	0.250	0.086	3.44	0.50–23.64	0.13	

Results are shown for two-sided tests based on a generalized linear model, including adjustment for sex and age. cf, carrier frequency. The ORs are relative to non-HLA-B*15:01 carriers.

We next examined the association between HLA-B*15:01 and asymptomatic infection in the combined UCSF prospective longitudinal COVID-19 Host Immune Response Pathogenesis (CHIRP) and Long-term Impact of Infection with Novel Coronavirus (LIINC) cohort. Here, 12 out of 82 individuals with European ancestry were identified as having an asymptomatic disease course. We found again that the carrier frequency of HLA-B*15:01 was exceptionally high (25%) in asymptomatic individuals compared with in symptomatic patients (8.6%). Although the power was somewhat limited by the sample size, and therefore cannot be considered to be a fully independent replication, the findings are strongly trending in support of our finding of a strong association of this allele with asymptomatic disease (*P* = 0.13, OR = 3.44, 95% CI = 0.50–23.64; Table 1). Finally, a meta-analysis across all three datasets (Citizen Science, UK, CHIRP/LIINC) confirmed the strong and consistent association of HLA-B*15:01 with asymptomatic infection (*P* < 10^−4^, OR = 2.55, 95% CI = 1.73–3.77; Supplementary Fig. 1).

## Unexposed T cells react to SARS-CoV-2

**Fig. 1:**
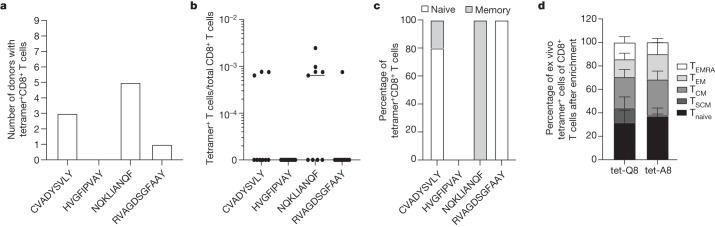
T cell reactivity in pre-pandemic samples from individuals carrying HLA-B*15:01. **a**–**c**, Ex vivo combinatorial tetramer analysis for the four indicated peptides was performed in nine pre-pandemic donor samples. **a**,**b**, The number of donors with detectable tetramer^+^CD8^+^ T cells (**a**) and their frequencies (**b**) are shown. **c**, The proportion of naive (CD45RA^+^CCR7^+^) and memory (combination of CD45RA^−^CCR7^−^, CD45RA^−^CCR7^+^, CD45RA^+^CCR7^−^) cells among tetramer^+^CD8^+^ T cells (based on *n* = 9 samples). **d**, Phenotypic analysis after TAME of ex vivo tetramer^+^ NQK-Q8-specific (tet-Q8) and NQK-A8-specific (tet-A8) T cells in 7 and 6 donors, respectively. Cell types were defined as follows: T_naive_ (CD45RA^+^CCR7^+^CD95^−^); T_SCM_ (stem cell memory, CD45RA^+^CCR7^+^CD95^+^); T_CM_ (central memory, CD45RA^−^CCR7^+^); T_EM_ (effector memory, CD45RA^−^CCR7^−^); T_EMRA_ (terminally differentiated, CD45RA^+^CCR7^−^). Data are mean ± s.e.m.

Owing to their high avidity for their cognate T cell receptors, pHLA (peptide–HLA) tetramers have been systematically used to visualize and quantify low-frequency antigen-specific T cells ex vivo using flow cytometry^23^. We focused on four SARS-CoV-2 epitopes (CVADYSVLY, HVGEIPVAY, NQKLIANQF and RVAGDSGFAAY) that were previously shown to elicit cellular immunity mediated by cytotoxic CD8^+^ T cells in patients with COVID-19 carrying HLA-B*15:01 (refs. ^24–28^). We next performed ex vivo pHLA tetramer evaluation with the SARS-CoV-2 peptides to detect antigen-specific CD8^+^ T cells in nine pre-pandemic peripheral blood mononuclear cell (PBMC) samples. We observed tetramer^+^CD8^+^ T cells for three of the SARS-CoV-2 epitopes (Fig. 1a,b, Supplementary Tables 5 and 6 and Supplementary Figs. 2 and 3).

NQKLIANQF (hereafter, NQK-Q8) was detectable in the highest proportion of samples (5 out of 9; 55.6%). Notably, in those donors, 100% of ex vivo NQK-Q8 tetramer^+^CD8^+^ T cells were memory T cells, indicating pre-existing T cell immunity against SARS-CoV-2 in a subset of individuals carrying HLA-B*15:01 who did not have any previous contact with the virus (Fig. 1c and Supplementary Table 5).

**Extended Data Fig. 1:**
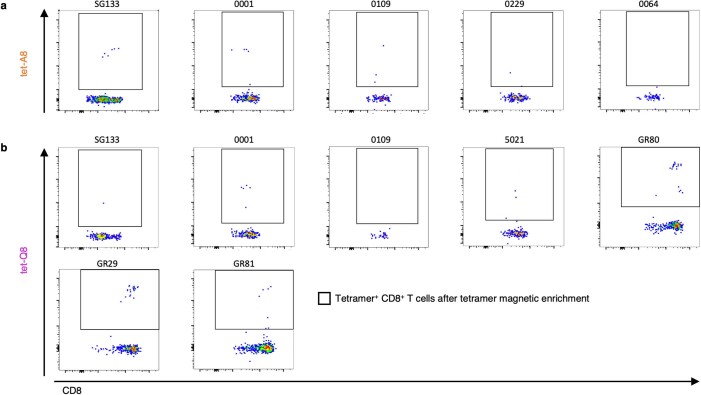
*Ex vivo* raw data from pre-pandemic samples from individuals carrying HLA-B*15:01. (**a–b**) Individual FACS plots for each pre-pandemic donor samples used for *ex vivo* tetramer staining following tetramer magnetic enrichment with tet-A8 tetramer (**a**) and with tet-Q8 tetramer (**b**), from 5 and 7 donors, respectively. The enriched tetramer^+^ CD8^+^ T cells observed are indicated within the inner square.

**Extended Data Fig. 2:**
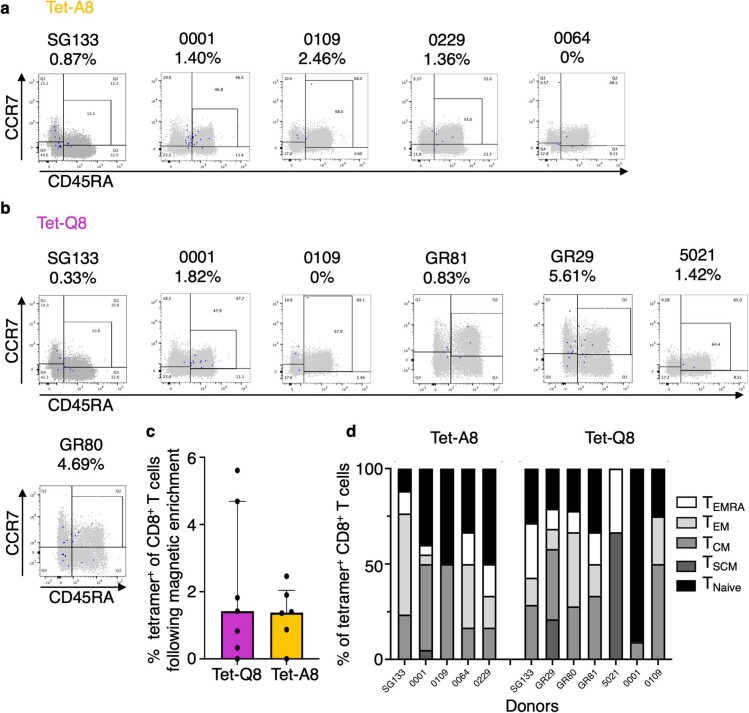
*Ex vivo* tetramer and phenotype analysis of NQK-Q8- and NQK-A8-specific T cells, related to Fig. 1d. (**a,b**) FACS plots of NQK-A8-tetramer^+^ CD8^+^ T cells following tetramer magnetic enrichment (**a**) and NQK-Q8-tetramer^+^ CD8^+^ T cells (**b**). The NQK-A8- and NQK-Q8- tetramer^+^ CD8^+^ T cells shown as blue dots were plotted versus CD3^+^ T cells shown as grey dots and gated on CCR7 and CD45RA to determine memory status, following tetramer magnetic enrichment for each donor. (**c**) The percentage of tetramer^+^ of CD8^+^ T cells after tetramer magnetic enrichment is reported for each donor with the purple bar for the Tet-Q8 tetramer and the orange bar for the Tet-A8 tetramer (n = 7 and n = 6 biologically independent samples, respectively). Data are presented as median values with IQR (interquartile range). The individual percentage for each donor is indicated on panels **a** and **b**. (**d**) The proportion of T_naïve_ (CD45RA^+^CCR7^+^CD95^-^); T_SCM_ (stem cell memory, CD45RA^+^CCR7^+^CD95^+^); T_CM_ (central memory, CD45RA^-^CCR7^+^); T_EM_ (effector memory, CD45RA^-^CCR7^-^); T_EMRA_ (terminally differentiated, CD45RA^+^CCR7^-^) cells in different donors.

NQK-Q8 was previously identified as immunodominant^28^, and these authors also demonstrated that NQK-Q8-specific T cells from HLA-B*15:01^+^ patients with infection were cross-reactive to the highly homologous peptide NQKLIANAF (hereafter, NQK-A8) from the seasonal coronaviruses HKU1-CoV and OC43-CoV. Given the high level of NQK-Q8-specific memory T cells compared with naive CD8^+^ T cells observed in the initial set of pre-pandemic samples that we tested (Fig. 1c), we sought to determine whether the same phenotype was also observed for the peptide NQK-A8 in additional samples from HLA-B*15:01^+^ individuals with no exposure to SARS-CoV-2. We performed ex vivo tetramer magnetic enrichment (TAME) with tetramers of each peptide, NQK-Q8 and NQK-A8, bound to HLA-B*15:01 (tet-Q8 and tet-A8, respectively) (Fig. 1d, Extended Data Figs. 1 and 2 and Supplementary Fig. 4) using a larger number of additional samples (*n* = 7 and *n* = 5 for tet-Q8 and tet-A8, respectively). We used pre-pandemic samples from the USA and Australia according to our previous study^25^. All of these samples exhibited tetramer^+^CD8^+^ T cells. Overall, we observed NQK-Q8-specific T cells in 75% of HLA-B*15:01^+^ donors with no previous exposure to SARS-CoV-2 (*n* = 12 out of 16; Fig. 1a–d and Extended Data Fig. 1).

We next analysed the phenotype of the tetramer^+^CD8^+^ T cells for both NQK peptides (Fig. 1d). We observed an abundance of memory T cells specific for NQK-Q8 in pre-pandemic samples (Fig. 1d and Extended Data Fig. 2). A similar phenotypic profile was observed for the NQK-A8-specific T cells, with most tetramer^+^CD8^+^ T cells being memory cells (Fig. 1d and Extended Data Fig. 2). The high proportion of effector memory and effector memory re-expressing CD45RA (T_EMRA_) T cells (29% for NQK-Q8 and 31% for NQK-A8) indicates a potent T cell response towards these peptides, which is a desirable characteristic for protective immunity. We were able to detect ex vivo tetramer^+^CD8^+^ T cells for NQK-Q8 (*n* = 7) and NQK-A8 (*n* = 6) (Fig. 1d and Extended Data Fig. 1). Overall, our data show the presence of CD8^+^ T cells specific for both NQK peptides, with a similar phenotype and magnitude in unexposed donors.

## Cross-reactive NQK-specific T cells

**Fig. 2:**
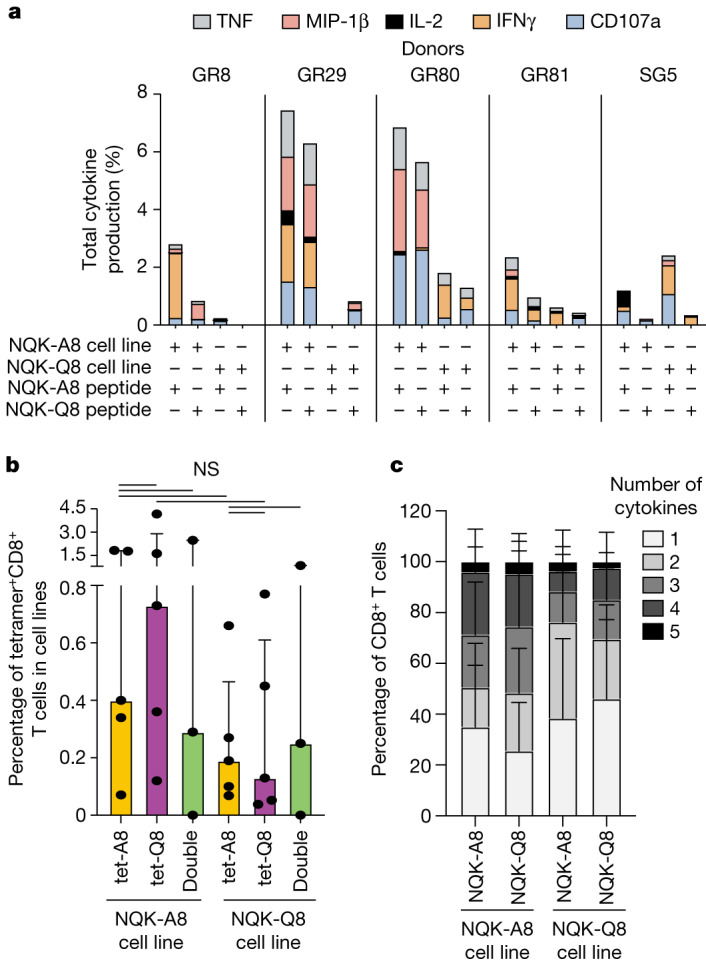
NQK-specific T cells are cross-reactive. **a**, Total cytokine production by CD8^+^ T cells in NQK-A8- and NQK-Q8-specific T cell lines. Each peptide-specific T cell line was restimulated individually with its cognate peptide or the homologous peptide as indicated, and the cytokine response was measured by intracellular cytokine staining (*n* = 5 donors). Percentages of effector functions (IFNγ, TNF, IL-2, MIP-1β, CD107a) minus the no peptide control are reported. **b**, In vitro tetramer analysis for the NQK-Q8- and NQK-A8-specific T cell lines (*n* = 5 donors). The cell lines were tetramer stained with a single tet-A8 (orange bar) or tet-Q8 (purple bar) tetramer or both tetramers (green bar). The frequency of tetramer^+^CD8^+^ T cells is shown. Data are median ± interquartile range. Differences between two groups were compared using two-tailed unpaired *t*-tests. *P* < 0.05 was considered to be significant. NS, not significant. **c**, Polyfunctionality analysis of CD8^+^ NQK-peptide-specific T cells from five unexposed donors. The number of functions is shown on a scale from 5 (black) to 1 (white). Data are the relative frequency (%) of total cytokine^+^CD8^+^ T cells. Data are mean ± s.e.m. Differences between two groups were determined using two-tailed unpaired *t*-tests. *P* < 0.05 was considered to be significant, and the result was not significant.

We next sought to determine whether the T cell cross-reactivity observed previously^28^ in patients with infection could be observed in unexposed individuals. We set up T cell lines with each peptide separately using PBMCs from unexposed and unvaccinated donors (*n* = 5, in vitro). Each cell line was then restimulated with either peptide. CD8^+^ T cell recognition and activation were determined by tetramer staining and intracellular cytokine staining, respectively (Fig. 2a and Supplementary Fig. 4).

All of the cell lines were characterized by the presence of tetramer^+^CD8^+^ T cells for both peptides (Supplementary Fig. 5), showing a bidirectional cross-reactivity whereby T cells can recognize both NQK peptides derived from the different viruses. The magnitude of tetramer^+^CD8^+^ T cells was different among donors and slightly higher for the cell lines generated with the NQK-A8 peptide (Fig. 2b). The co-staining of the T cell lines with both tetramers (tet-Q8, allophycocyanin (APC) conjugated; tet-A8, phycoerythrin (PE) conjugated) showed that the vast majority of T cells were cross-reactive (Supplementary Fig. 5).

We subsequently measured the level of T cell responsiveness to each peptide. T cells from all donors, apart from GR8, responded to the cognate peptide (Fig. 2a). In four out of five donors, a stronger response was observed in the NQK-A8-specific cell lines independent of the peptide (Fig. 2a). These findings were similar to what we previously observed for the N(105–113) peptides derived from SARS-CoV-2 and HKU1-CoV/OC43-CoV, in the context of HLA-B*07:02, whereby the T cells were more strongly stimulated after presentation of the peptide derived from seasonal coronavirus compared with the SARS-CoV-2-derived peptide in unexposed donors^25^.

**Extended Data Fig. 3:**
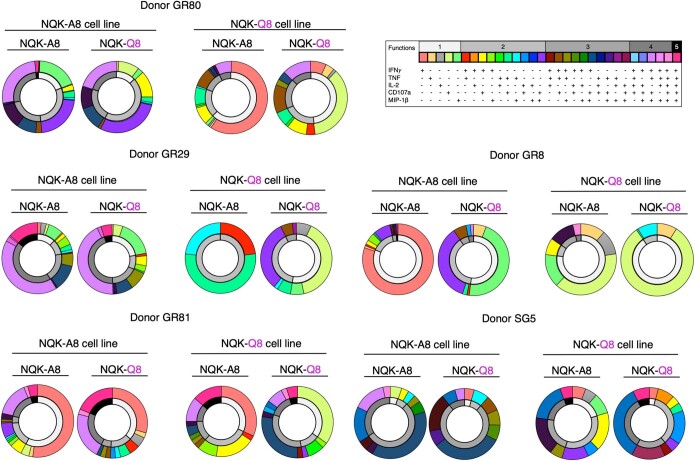
Functionality pie charts of T cell lines. Frequency of CD8^+^ T cells with different effector functions (IFNγ, TNF, IL-2, MIP-1β, CD107a) was determined, along with the number of different functions, minus the no peptide control. The outer ring of the double ring pie shows the function profile in different colours according to the table, and the inner ring represents the number of functions with black for 5 and white for 1.

Notably, although the percentage of cytokine^+^CD8^+^ T cells was different between donors (around 0–8%; Fig. 2a), the level and profile of cytokines were comparable for each cell line between the two peptides. This observation suggests that both NQK peptides stimulate T cells at similar magnitudes, which may reflect a high level of T cell cross-reactivity. We next examined the functional profile of the CD8^+^ T cells, which corresponds to their ability to exhibit different effector functions (IFNγ, TNF, IL-2, MIP-1β, CD107a; Fig. 2c). A highly polyfunctional T cell response, with up to five functions expressed, was observed for both cell lines that were restimulated with either peptide. At least 40% (*n* = 2 out of 5) and up to 80% (*n* = 4 out of 5) of donors had T cells exhibiting all five functions in one or more cell line. An average of 2.5–4.7% of CD8^+^ T cells exhibited all five functions tested (Fig. 2c and Extended Data Fig. 3).

In summary, the T cell response against NQK peptides from seasonal coronaviruses and SARS-CoV-2 in individuals never exposed to SARS-CoV-2 is highly cross-reactive and polyfunctional.

## NQK-specific T cells express public TCRs

**Fig. 3:**
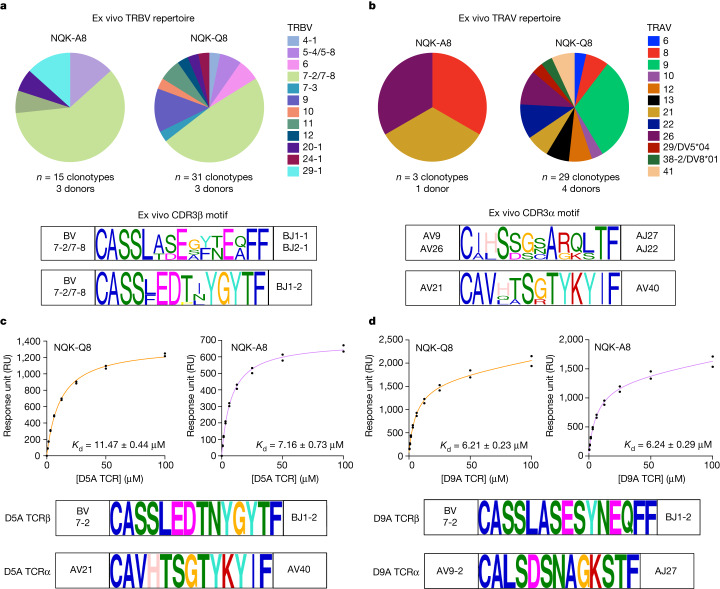
NQK-specific T cells are characterized by the presence of high-affinity public TCRs in unexposed donors. **a**,**b**, Analysis of the ex vivo TCR repertoire for NQK-A8- and NQK-Q8-specific T cells after TAME on the basis of TRBV (**a**) and TRAV (**b**) use. The pie charts show the percentage of each TRBV or TRAV used in the single-cell sorted clonotypes. The CDR3β and CDR3α sequence motifs are shown below the pie charts, respectively, for the TCRs with the biased expression of TRBV7-2/7-8 paired with TRAV9/26 or TRAV21, including the public TCRs. **c**,**d**, The binding response (response units (RU)) of D5A (**c**) and D9A (**d**) TCRs (analyte) against HLA-B*15:01–NQK complexes (A8 in orange and Q8 in purple). The public TCR D5A expresses TRBV7-2 paired with TRAV21, and the public D9A TCR expresses TRBV7-2 paired with TRAV9-2; the CDR3β and CDR3α sequences for each public TCRs are shown below the binding curves. The SPR steady-state binding curves represent binding under a TCR concentration range of 0.39–100 μM. *n* = 2 biologically independent experiments performed in duplicate; the graph shows the results from one experiment performed in duplicate, represented by the black dots.

**Extended Data Fig. 4:**
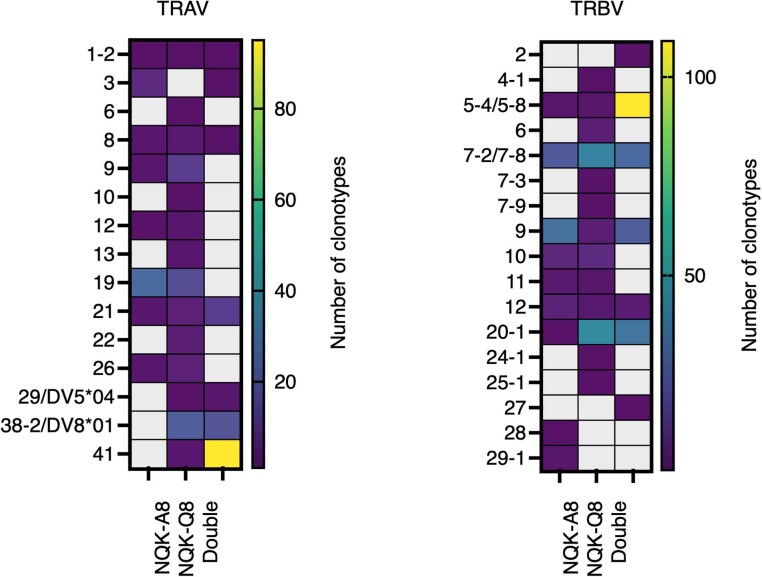
TRAV and TRBV usage specific for each tetramer used in unexposed individuals. Heatmaps displaying preferred TRAV (left) and TRBV (right) usage of NQK-Q8-, NQK-A8-, or both (double) peptide-specific TCRs in all unexposed donors collectively.

**Extended Data Fig. 5:**
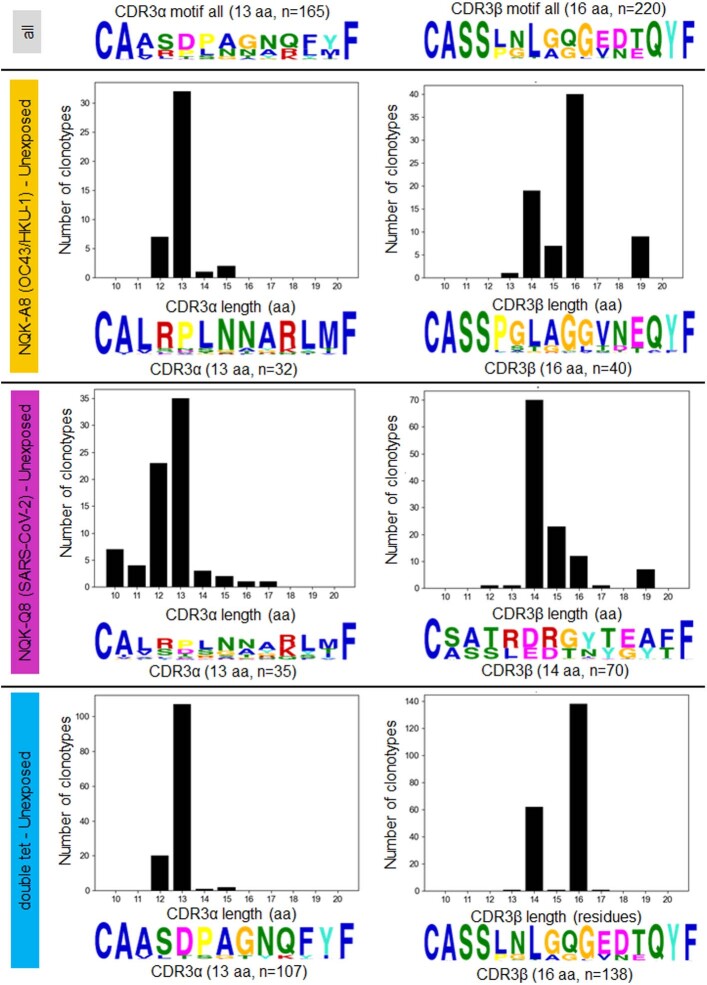
NQK-specific TCR repertoire CDR3 analysis in unexposed donors. Summary of CDR3α (left) and CDR3β (right) lengths and motif analysis for the NQK-A8 (top), NQK-Q8 (middle), and both peptides (bottom)-specific TCR clonotypes in unexposed donors. The MEME motif discovery program was used to identify aa motifs enriched; the relative size of each residue symbol is proportional to its frequency, while the total height of aa symbols indicates the information content of the position in bits. The motif on top left panel is the CDR3α 13 amino acid (aa) long sequence motif derived from all CDR3α sequences obtained (n = 165); on the right top panel is the CDR3β 16 aa long sequence motif derived from all CDR3β sequences obtained (n = 220).

We next determined the T cell receptor (TCR) repertoire specific to both NQK-A8 and NQK-Q8 peptides. Using single-cell sorting of tetramer^+^CD8^+^ T cells and TCR sequencing, we obtained 456 productive clonotypic sequences from eight unexposed donors and one triple-vaccinated donor (Supplementary Table 7). The TCR repertoires of T cells specific to each or both NQK peptides (single or double tetramer^+^CD8^+^ T cells) were obtained from both T cell lines (in vitro) and unstimulated PBMCs (ex vivo) (Fig. 3a,b, Supplementary Table 7, Extended Data Figs. 4 and 5 and Supplementary Fig. 6).

The NQK-specific TCR repertoires overlap extensively with shared clonotypes and public TCRs (Supplementary Tables 7 and 8). The ex vivo TCR repertoire was strongly biased with clonotypes expressing TRAV9 (31%) or TRAV21 (15%) paired with the dominant TRBV7-2/7-8 (71%) (Fig. 3a,b and Supplementary Table 7). Although the TRBV7-2/7-8 clonotypes were also present in the in vitro repertoire, their frequency was decreased (11%); we also observed expansion of the TRBV5-4/5-8^+^ (35%), TRBV9^+^ (18%) and TRBV20-1^+^ (24%) clonotypes. Similarly, a frequency shift for TRAV gene usage was observed in the in vitro TCR repertoire with a decrease in TRAV21^+^ clonotypes (6%), an absence of TRAV9^+^ clonotypes, and the emergence of TRAV19^+^ (21%), TRAV38-2/DV8*01^+^ (19%) and TRAV41^+^ (47%) clonotypes. The TRAV38-2/DV8*01^+^ and TRAV41^+^ clonotypes were expanded in only one donor each (GR81 and GR80, respectively), and these paired only with TRBV20-1 and TRBV5-4/5-8, respectively; these expanded clonotypes were cross-reactive (Supplementary Table 7). The CD8^+^ T cells able to bind both tet-A8 and tet-Q8 primarily expressed TRBV7-2/7-8 (42%) paired with TRAV21 (54%); TRBV9 (36%, pairing unknown); or TRBV5-4/5-8 (12%) paired with TRAV41 (36%) (Supplementary Table 7). Moreover, the CDR3 loop length and sequence motifs shared similarities (Fig. 3 and Extended Data Fig. 5). Public TCRs, defined as identical TCRs shared between individuals, were also isolated^29^. The public NQK-specific TCRs were TRBV7-2/7-8^+^ paired with TRAV21 or TRAV9-2 and were observed both ex vivo and in vitro in unexposed donors (Fig. 3c,d and Supplementary Table 8). These public and cross-reactive TCRs have also been isolated from donors who had recovered from COVID-19 and/or vaccinated donors previously^28^ (Supplementary Table 8). This finding shows the potential for a strong pre-existing immune memory pool of cross-reactive public TCRs specific to the NKQ peptides that is similar to that observed after infection. The phenotypic profiles of the clonotypes expressing the public TCRs ex vivo in unexposed donors were either stem memory or central memory T cells.

To summarize, the NQK-Q8- and NQK-A8-specific TCR repertoires are biased with the presence of public and cross-reactive TCRs in unexposed donors, comparable to observations in donors who have recovered from COVID-19 and/or vaccinated donors (Supplementary Figs. 6 and 7). These findings suggest that the existence of memory cross-reactive NQK-specific TCRs before infection could have a critical role in the immune response to SARS-CoV-2, contributing to asymptomatic infection in individuals carrying HLA-B*15:01.

## NQK peptides share structure similarity

The HLA-B*15:01-restricted peptide NQK-Q8 is conserved among all SARS-CoV-2 variants, even the new XBB variant of Omicron, and differs by only one amino acid from the HKU1-CoV/OC43-CoV peptide (Supplementary Fig. 8). Importantly, NQK-Q8-reactive T cells from HLA-B*15:01^+^ individuals infected with SARS-CoV-2 were previously shown to be cross-reactive to the homologous peptide from seasonal coronaviruses^28^. As we have shown the presence of pre-existing T cell immunity in pre-pandemic samples and cross-reactivity of the NQK peptide-specific T cells (Figs. 1 and 2), we sought to investigate whether the amino acid change in the NQK peptides from SARS-CoV-2 and the seasonal coronaviruses HKU1-CoV and OC43-CoV affects the stability of HLA-B*15:01.

**Fig. 4:**
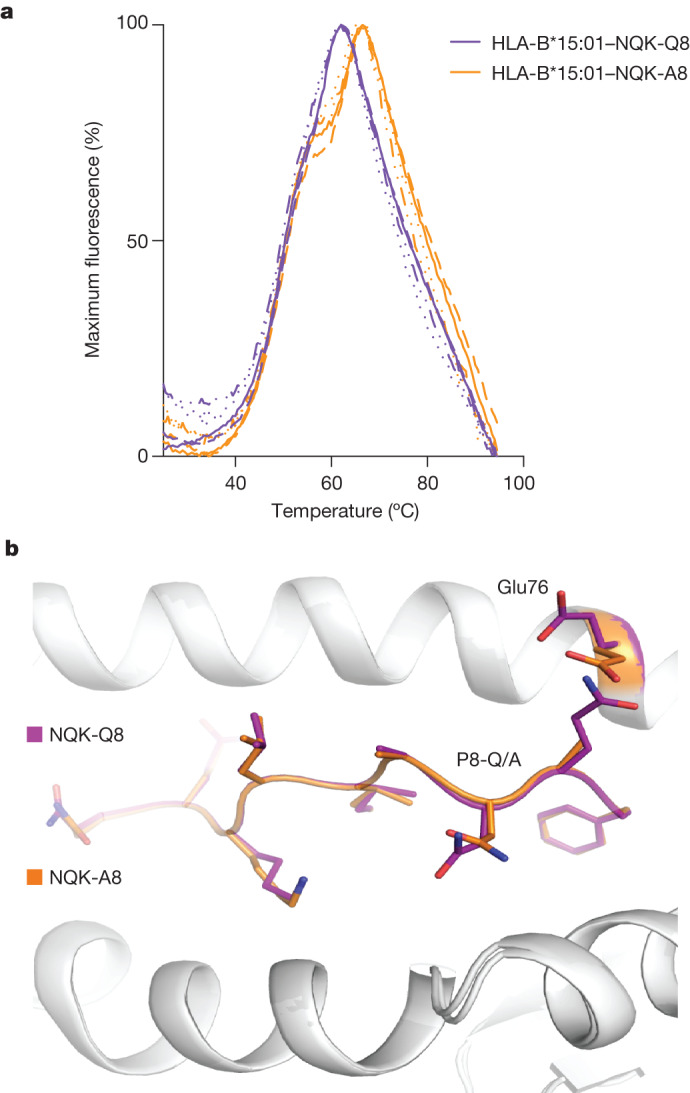
NQK peptides are stable and adopt the same conformation bound to the HLA-B*15:01 molecule. **a**, DSF plots showing the normalized fluorescence intensity versus temperature for HLA-B*15:01 in a complex with the NQK-Q8 (purple) or NQK-A8 (orange) peptide measured at concentrations of 5 μM and 10 μM. *n* = 2 biologically independent experiments performed in duplicate, represented by the different lines. **b**, Superimposition of the crystal structures of HLA-B*15:01 (white cartoon) in a complex with either the NQK-Q8 (purple stick) or the NQK-A8 (orange stick) peptide.

**Extended Data Fig. 6:**
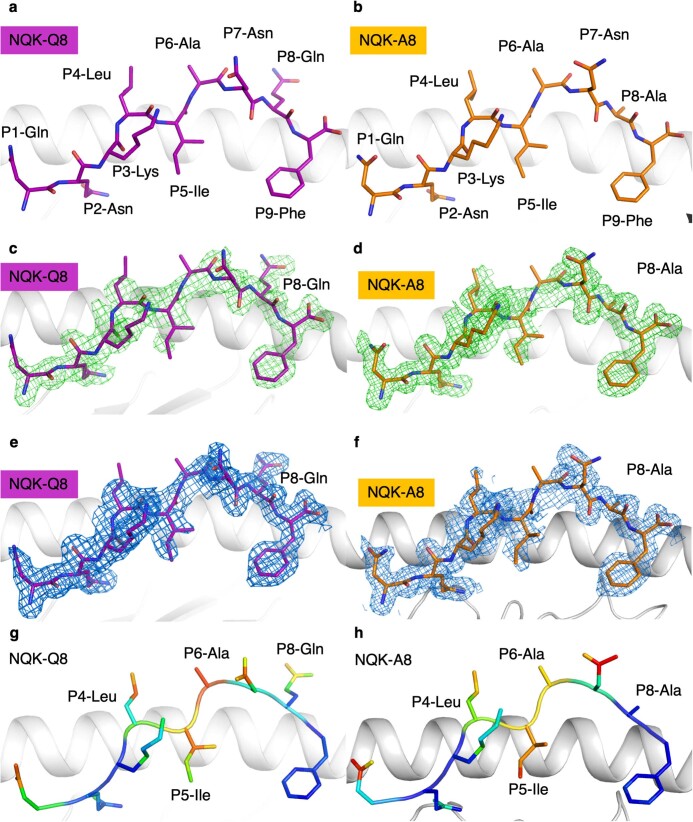
HLA-B*15:01 interaction with peptides derived from SARS-CoV-2 and seasonal coronaviruses. (**a–b**) Structures of HLA-B*15:01-NQK-Q8 (peptide as purple sick) (**a**) and HLA-B*15:01-NQK-A8 (peptide as orange stick) (**b**), with the HLA chain in white cartoon. (**c–d**) Electron density map around the NQK-Q8 (purple stick) and NQK-A8 (orange stick) peptides, respectively, in green at 3s for the Fo-Fc map. (**e–f**) Electron density map around the NQK-Q8 (purple stick) and NQK-A8 (orange stick) peptides, respectively, in blue at 1s for the 2Fo-Fc map. (**g–h**) B_factor_ analysis of the atoms of the NQK-Q8 and NQK-A8 peptides, respectively. The atoms are coloured according to their B_factor_ that indicates the mobility of each atom from low (blue) to high (red).

We refolded the HLA-B*15:01 molecule in the presence of each peptide and performed a thermal melt assay using differential scanning fluorimetry (DSF). Both pHLA complexes exhibited the same thermal melting point (*T*_m_; Fig. 4a and Supplementary Table 9), indicating that the Gln>Ala amino acid change does not affect the overall stability of the pHLA. We crystallized each peptide in a complex with HLA-B*15:01 and solved their structures at high resolution (Fig. 4b, Extended Data Table 2 and Extended Data Fig. 6). Overall, NQK-Q8 adopted a canonical conformation within the antigen-binding cleft of the HLA-B*15:01 molecule^30^. The Gln at position 2 (P2) was deeply inserted into the B pocket of HLA-B*15:01, whereas the P9-Phe primary anchor residue bound to the F pocket. The central part was more mobile than the rest of the peptide (Extended Data Fig. 6). The NQK-Q8 peptide exposed to the solvent, and potentially to circulating T cells, five of its nine residues (P1-Asn, P4-Leu, P6-Ala, P5-Asn and P8-Gln). The NQK-A8 peptide bound similarly in the HLA-B*15:01 cleft (Fig. 4b and Extended Data Fig. 6). The superposition of the two pHLA structures revealed very little difference between the two complexes, with a root mean squared deviation of 0.08 Å for the Cα atoms of the antigen-binding cleft (residues 1–180) and 0.12 Å for the peptides. Only some local rearrangement around the P8 of the peptide was observed with a shift of the Glu76 side chain to avoid steric clashes with the P8-Gln (Fig. 4b). This change, which is on the surface of the peptide, could affect T cell interaction and might change the TCR affinity. To test the effect of the P8 difference within the NQK peptides, we selected some representative TCRs to perform affinity measurements using surface plasmon resonance (SPR). We selected the three TRBV7-2^+^ public TCRs paired with either TRAV9-2 (D9A TCR) or TRAV21 (A6A and D5A TCRs) (Fig. 3c,d and Supplementary Table 8).

**Extended Data Fig. 7:**
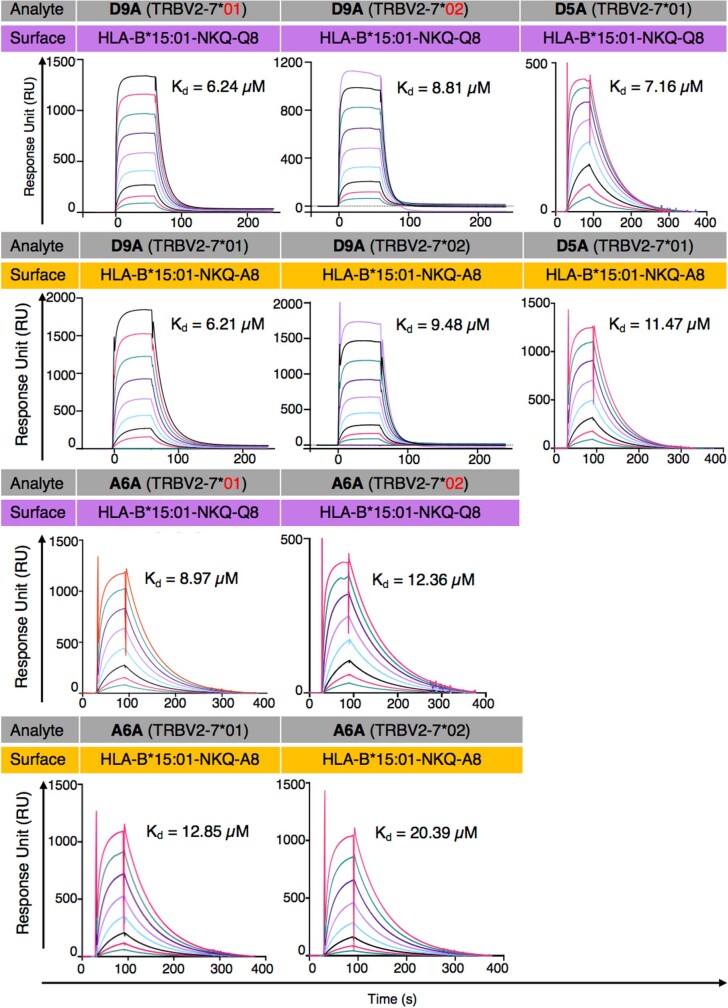
Surface plasmon resonance sensorgrams. SPR sensograms for the 5 tested TCRs with each curve showing different concentration of the TCR.

**Extended Data Fig. 8:**
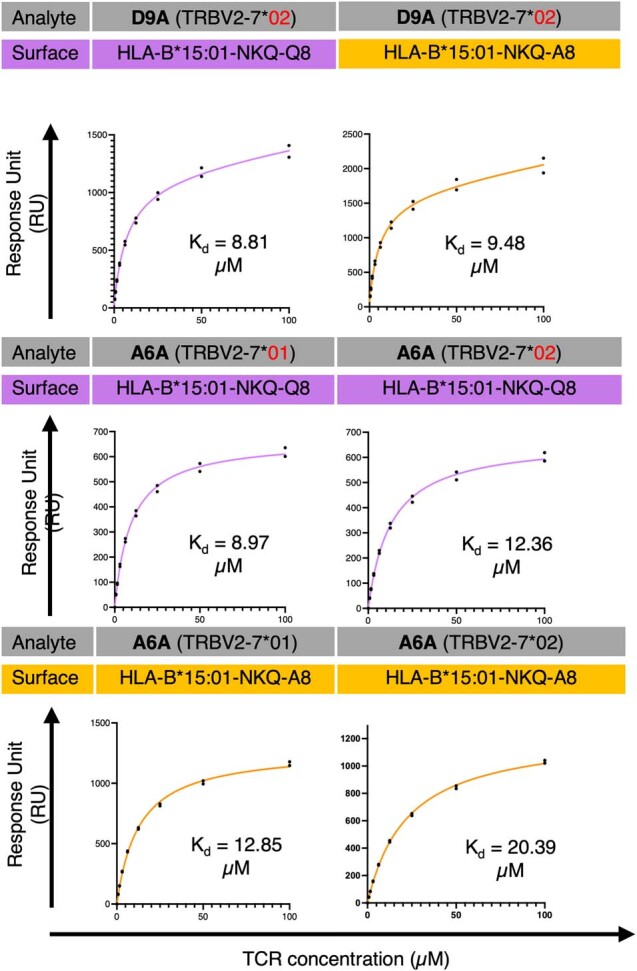
Steady-state binding curves. Steady-state binding curve for the five tested TCRs towards HLA*15:01-NKQ-Q8 or -A8 complex. Analytes (TCRs) flowed over immobilized HLA*15:01-NKQ-Q8 or -A8 with a concentration range of 0 to 100 μM. n = 2 biologically independent experiments performed in duplicate, with the graph showing results from one experiment.

All of the TCRs tested were able to bind with high affinity (*K*_d_ range, 6–20 μM) to both the NQK-A8 and NQK-Q8 peptides presented by the HLA-B*15:01 molecule (Fig. 3c,d, Extended Data Figs. 7 and 8 and Supplementary Table 10). We observed a slower dissociation rate for the two TRAV21^+^ TCRs (A6A and D5A TCRs) compared with the TRAV9^+^ D9A TCR (Extended Data Fig. 7). The TCRs also differ in their CDR3β loops.

In a previous study^28^, the cross-reactivity of two NQK-Q8-specific TCRs (TCR1 and TCR2) transfected into Jurkat cells was shown. The published TCR1 and TCR2^28^ differ by only one residue in each CDR3 loop from A6A and D5A TCRs, respectively (Supplementary Table 8). This is reflected by the similar affinity of the A6A and D5A TCRs for the NQK-A8 and NQK-Q8 peptides observed here (Supplementary Table 10). Overall, our data show that the similar conformation and ability to stabilize the HLA-B*15:01 molecule of the two NQK peptides underpin the similar affinity observed for the public TCRs. Moreover, the high affinity of these TCRs towards the NQK peptides might trigger a rapid expansion of these memory cells after SARS-CoV-2 infection.

## Discussion

Understanding the biological underpinning of asymptomatic infection with SARS-CoV-2 has important implications for public health measures, vaccine design and therapeutic development. Here we provide evidence of a genetic basis and the mechanistic explanation underlying asymptomatic disease. Leveraging a large database and mobile technology in this crowd-sourced study, we reveal important insights into the immunogenetic underpinnings of asymptomatic SARS-CoV-2 infection. Our use of a mobile application and a pre-existing database for medical research enabled us to screen nearly 30,000 individuals who were previously genotyped for *HLA* for viral infection and disease course. We augment our findings of a strong *HLA* association with asymptomatic disease course in this unique cohort with functional and structural studies to support a model of pre-existing immunity to explain the observed *HLA* association.

We show that, among participants reporting a positive test result for SARS-CoV-2, HLA-B*15:01 is significantly associated with asymptomatic infection. We observed that individuals carrying this common allele (approximately 10% in individuals with European ancestry) are more than twice as likely to remain asymptomatic after SARS-CoV-2 infection compared with those who do not, and a notable effect of HLA-B*15:01 homozygosity increasing the chance of remaining asymptomatic by more than eight times. This suggests important features of early infection with SARS-CoV-2. Supporting the role of HLA-B*15:01 in mediating asymptomatic infection, we found a highly similar frequency distribution of this allele in asymptomatic versus symptomatic patients in two independent cohorts.

Despite a growing number of published studies, the role of *HLA* variation in COVID-19 has remained unclear, with no clear consensus in the literature to date and, notably, few studies examining asymptomatic infection as a primary phenotype^18^. Our reanalysis of the primary data underlying a reported association between HLA-DRB1*04:01 and asymptomatic infection^22^ uncovered clear evidence for the role of HLA-B*15:01 in asymptomatic disease, which was not reported in the initial study. Although the data in our discovery cohort did not corroborate the association for HLA-DRB1*04:01 alone, we did find that this allele enhanced the effect of HLA-B*15:01 when the pair were in combination. Note that this is the *HLA-DRB1* allele that is most commonly associated with HLA-B*15:01 in individuals in the United States who self-identify as white^31^ and it is therefore difficult to differentiate a real effect from one related to linkage disequilibrium between these loci unless directly tested. Similarly, another recent paper describing an association of *HLA-DRB1* alleles with asymptomatic infection did not genotype for *HLA-B*^32^. Finally, two other large studies that used patient questionnaires regarding symptoms did not consider the mildest symptoms that are common in SARS-CoV-2 infection (for example, runny nose and scratchy throat), resulting in a much less stringent definition of asymptomatic infection than we considered here^33,34^.

Respiratory tract infections are a major public health concern, representing a substantial burden, particularly for young children and the elderly^35,36^. Four strains of seasonal coronaviruses (229E-CoV, NL63-CoV, OC43-CoV and HKU1-CoV) represent 15% to 30% of all respiratory tract infections every year^37^. Notably, previous studies have shown that T cells can cross-react to SARS-CoV-2 and seasonal coronavirus peptides, indicating that long-lasting T cell protective immunity can potentially limit the severity of COVID-19^25^. Moreover, a recent study^28^ demonstrated T cell cross-reactivity to SARS-CoV-2 and seasonal coronaviruses for an HLA-B*15:01-restricted immunodominant epitope (NQK-Q8) in individuals who received two doses of the Pfizer-BioNTech BNT162b2 mRNA vaccine. To test the hypothesis that HLA-B*15:01 can mediate asymptomatic disease through pre-existing T cell immunity, we analysed immunodominant epitopes in T cells from human PBMCs from pre-pandemic healthy individuals. We observed that T cells from a subset of healthy donors carrying HLA-B*15:01 who were never exposed to SARS-CoV-2 were reactive to the SARS-CoV-2 peptide NQK-Q8, and most of the reactive cells displayed a memory phenotype. The sequence identity between SARS-CoV-2 and seasonal coronaviruses peptides, except for a single amino acid substitution, could explain the T cell cross-reactivity. However, a direct demonstration that peptides from SARS-CoV-2 and the seasonal coronaviruses OC43-CoV and HKU1-CoV are stable in the HLA-B*15:01 cleft was necessary to further corroborate our hypothesis.

Through our examination of the crystal structures of the HLA-B*15:01 molecule in the presence of each peptide, we demonstrated that both NQK-Q8 (SARS-CoV-2) and NQK-A8 (OC43-CoV and HKU1-CoV) spike peptides share a similar ability to stabilize the HLA-B*15:01 molecule, and are presented in a similar conformation by HLA-B*15:01, providing the molecular basis for T cell cross-reactivity and pre-existing immunity. This observation is in accordance with previous research in uninfected HLA-B*07:02^+^ individuals who are able to recognize the N(105–113) peptide derived from SARS-CoV-2 due to the presence of cross-reactive T cells recognizing the homologous N(105–113) peptide from OC43-CoV and HKU1-CoV^25^. Notably, this T cell cross-reactivity has been associated with less severe COVID-19 disease^38^.

Our data show that both seasonal and pandemic coronavirus-derived NQK peptides can lead to a highly polyfunctional T cell response in the context of HLA-B*15:01, with T cells able to exhibit different effector functions (IFNγ, TNF, IL-2, MIP-1β, CD107a). T cell polyfunctionality is critical as it can lead to superior viral-suppressive activity^39,40^; it is linked with high-affinity TCRs that can detect low levels of antigens^39,41^, and it is predictive of protective immunity and vaccine efficacy^42–44^. The high level of polyfunctionality observed for the CD8^+^ T cells towards the NQK-Q8 peptide in unexposed individuals contrasts with the moderate polyfunctionality observed for the HLA-B*07:02-restricted SARS-CoV-2 N(105–113) peptide (SPRWYFYYL) that also shares a high sequence similarity with the OC43/HKU-1-CoV N(105–113) peptide (LPRWYFYYL)^25^. Although there was some biased TCR gene usage observed in HLA-B*07:02-N(105–113)-specific TCR repertoire, we did not observe the presence of public TCRs^25^. By contrast, we observed that HLA-B*15:01–NQK-specific TCR repertoire was characterized by the existence of public TCRs. The memory phenotype of the public TCRs in unexposed donors also strongly suggests that they might provide a protective advantage to HLA-B*15:01^+^ donors infected by SARS-CoV-2.

The presence of a high level of memory, polyfunctional, high-affinity and public cross-reactive T cells in unexposed donors probably underpins the strong association between the allele HLA-B*15:01 and asymptomatic SARS-CoV-2 infection. The presence of cross-reactive public TCRs before infection, observed also after infection or vaccination^28^, could provide a fast and protective immune response in individuals carrying HLA-B*15:01. This characteristic could make HLA-B*15:01 a generally more protective allele than HLA-B*07:02, which has been described as potentially limiting the severity of COVID-19 disease^38^. Our results also show the importance of pre-existing immunity^25,28,45,46^ giving rise to a memory pool of cross-reactive T cells ready to fight infection^23,37^.

Examination of T cells in patients with asymptomatic SARS-CoV-2 infection has suggested robust T cell responses similar to those with symptomatic disease^47^. Recent studies have shown that SARS-CoV-2-specific memory T cells are enriched at the site of infection compared with in the blood^48^. Thus, the low frequencies of memory T cells that we observed in the blood are probably an under-representation of the resident antigen-specific memory T cells in the respiratory tract that rapidly respond to antigen restimulation at the viral entry sites. Presumably, a pre-existing resident memory T cell population at viral entry sites can lead to a rapid viral clearance before the overt onset of symptoms. Furthermore, the finding that T cells in asymptomatic infection secrete higher quantities of IFNγ compared with those in symptomatic patients early in infection^44,47^ supports a role for memory T cells at this stage^49^. Although the current literature is mixed regarding cross-reactive CD8^+^ T cells specific to SARS-CoV-2, this might be explained by HLA specificity^50,51^.

Together, our results strongly support the hypothesis that HLA-B*15:01 mediates asymptomatic COVID-19 disease through pre-existing T cell immunity due to previous exposure to HKU1-CoV and OC43-CoV. Notably, the NQK-Q8 peptide is conserved among SARS-CoV-2 variants; moreover, among all HLA-B15:01-restricted SARS-CoV-2-derived T cell epitopes reported in the Immune Epitope Database (Supplementary Fig. 8), no other epitope exhibits high sequence identity across common coronaviruses, except for the replicase polyprotein 1ab peptide QLYLGGMSY. However, this last epitope has not been reported in the literature as immunodominant in patients positive for HLA-B*15:01 and SARS-CoV-2. On the basis of the limited data available regarding known HLA-B*15:01 epitopes in SARS-CoV-2 patients, NQK-Q8 remains the prime candidate peptide underlying any HLA-B*15:01-mediated T cell cross-immunity with seasonal coronaviruses.

One limitation of this study is that all of the testing results and symptoms in our discovery cohort are self-reported. We recognize that this may result in some margin of error in our results. However, we have previously validated this approach by verifying test results in a subset of the participants^52^. Similarly, we did not query some symptoms, notably those related to rash and simple nasal congestion (as opposed to runny nose, which we consider here), and did not consider individuals with only a single symptom report within our two-week window as symptomatic, which may have resulted in some individuals being categorized as asymptomatic when in fact they experienced mild symptoms. However, we incorporated an additional ‘sanity check’ into our classification of asymptomatic disease, where we considered the response to the survey question regarding their reasons to seek testing for SARS-CoV-2 infection. Thus, although our self-report methodology means that we cannot definitively state that our asymptomatic cohort was entirely free of any symptoms (and in some cases may, rather, be considered mildly symptomatic), we feel confident that our classification of individuals as asymptomatic was generally robust. Importantly, we find a very consistent genetic association across the study and in two independent cohorts where asymptomatic disease was clinician-defined, pointing to a true biological feature.

Another limitation is that our association results are limited to individuals who self-identify as white. While our study cohorts were not well-powered in this regard, we find that our results for HLA-B*15:01 appear to trend similarly in Black individuals, although this result is less clear in Asian and Hispanic individuals (Supplementary Table 11). However, owing to the paucity of individuals combined with the lower frequency of this allele in some populations, it is impossible to conclude whether our results for HLA-B*15:01 association with asymptomatic disease are applicable in these ancestries. A final limitation is that we tested only four SARS-CoV-2 peptides in our ex vivo analysis. The search for additional candidate peptides will be facilitated as more studies analyse T cell reactivity in patients carrying HLA-B*15:01, similarly to a previous study^28^. However, we identified at least one SARS-CoV-2 peptide previously known to be immunodominant in SARS-CoV-2 infection that was reactive to memory T cells from HLA-B*15:01^+^ individuals collected before the pandemic.

In summary, we have demonstrated a strong and significant association of a common *HLA* class I allele, HLA-B*15:01, with asymptomatic infection with SARS-CoV-2. We demonstrated that HLA-B*15:01^+^ T cells from pre-pandemic samples were reactive to an immunodominant SARS-CoV-2 peptide that shares high sequence similarity with peptides from seasonal coronaviruses. We provided the molecular basis of T cell cross-reactivity by showing that HLA-B*15:01 can stabilize and present peptides from HKU1-CoV and OC43-CoV similarly to the immunodominant peptide from SARS-CoV-2. Moreover, we show that public clonotypes were cross-reactive, polyfunctional and able to recognize both NQK peptides with high affinity. Our results have important implications for understanding early infection and the mechanism underlying early viral clearance and may lay the groundwork for refinement of vaccine development and therapeutic options in early disease.

## Methods

### Data collection for the discovery (Citizen Science) cohort

The study participants were volunteer bone marrow donors with valid e-mail addresses on file with the National Marrow Donor Program (NMDP) who were invited to participate in the study through an e-mail outreach campaign that began in July 2020. All individuals had within the NMDP database a pre-existing record for high-resolution *HLA* genotyping, typically for five loci (*HLA-A, -B, -C, -DRB1* and *-DQB1*)^31^. Participants who opt in to the study are required to download a smartphone app and participate in the COVID-19 Citizen Science Study (launched using the Eureka Digital Research Platform; https://eureka.app.link/covid19) or, as of January 2021, participate through the website (https://covid19.eurekaplatform.org/). Once enrolled, the participants are asked to complete an initial 10 to 15 min survey about baseline demographics, their health history and daily habits. Follow-up daily questions specific to symptoms, weekly questions regarding testing and monthly questions regarding hospitalization for COVID-19 are delivered by push notification or text message on an ongoing basis and require 5 to 15 min per week. As of 30 April 2021, we enrolled 29,947 individuals, of whom 21,893 have completed their baseline survey (Supplementary Table 12). Participation in the UCSF Citizen Science study and linking to NMDP *HLA* data were approved by the Institutional Review Board for the University of California, San Francisco (IRB 17-21879 and IRB 20-30850, respectively). All of the participants provided written informed consent agreeing to research and publication of research results.

Within the mobile application, the survey respondents are asked during their initial baseline survey whether they have ever been tested for active infection and report the result (positive, negative, do not know) and the approximate number of weeks since the test. Thereafter, each week respondents are asked whether they were tested in the previous week, and to report the result. We considered anybody reporting a positive test for active infection as having been infected with SARS-CoV-2. Our cohort consisted of individuals reporting a positive test for virus up to 30 April 2021 before the implementation of widespread vaccination for the virus. We restricted the analysis to individuals who had self-identified as ‘white’ only due to insufficient numbers for analysis in the other groups (Supplementary Tables 13 and 14), allowing an analysis of 1,428 individuals. The inclusion criteria are provided in Supplementary Fig. 9.

Symptoms are self-reported at the baseline and in daily surveys. Within the baseline survey, the respondents are asked to report whether they had any of a list of symptoms (Supplementary Table 15) for 3 days or longer at any time since February 2020. These same symptoms are queried in each daily survey, where respondents are asked whether they experienced each symptom within the previous 24 h. Among those individuals, we considered those as asymptomatic who reported having had a positive test for active virus at the baseline, with a time since the test of longer than 2 weeks or who did not specify test dates, and who reported “None of the above” for all symptoms in the baseline survey. We also considered daily symptom reports for the 2 weeks after the baseline for respondents who reported a positive test for active infection at the baseline as having occurred within the previous 2 weeks. In these cases, we considered individuals asymptomatic if, in addition to reporting no symptoms at baseline, they did not report any single symptom two or more times within this time period. For individuals who did not report a positive test for active infection at the baseline, but subsequently reported a positive test on a weekly survey, we used the same criteria considering daily symptom reports for the period 2 weeks before and 2 weeks after the positive test report (Supplementary Fig. 10). To further confirm a lack of symptoms, we also considered the survey question “Why was the test for active COVID-19 infection (virus) performed?” (Supplementary Table 16). Individuals who otherwise did not report symptoms, but responded “I had symptoms concerning for COVID-19 infection” were categorized as symptomatic.

### The CHIRP and LIINC (replication) cohorts

The study participants were enrolled in two UCSF-based prospective longitudinal cohorts: the CHIRP study and the LIINC study. The participants were identified through local clinical systems (UCSF Moffitt Hospital, San Francisco General Hospital, Kaiser, California Pacific Medical Center) as well as the San Francisco Department of Public Health. After confirmation of SARS-CoV-2 test results or exposure to determine eligibility, the participants were asked to sign a consent form, complete a baseline visit and schedule follow-up in-person visits. The CHIRP study included volunteers with positive PCR test documentation and/or symptom onset within the preceding 21 days. Asymptomatic disease was defined as having a confirmed positive PCR test with lack of any symptoms (“Did you have or are you still having any symptoms that you think are because of COVID-19?”) at the baseline and follow-up visits. A total of five longitudinal samples were collected from participants with acute SARS-CoV-2 infection. The first sample was collected <31 days of symptom onset or <31 days from exposure to SARS-CoV-2 as a week 0 baseline visit. The remaining samples were collected at weeks 1, 3, 10 and 24. At each CHIRP visit, blood and nasopharyngeal swabs were collected. Optional sample collection included sputum, saliva, stool and urine. The LIINC study enrolled participants with previous SARS-CoV-2 infection confirmed on clinical nucleic acid amplification testing between 14 and 90 days after initial symptom onset. After written informed consent, clinical data and biospecimens were collected monthly for up to 4 months after initial symptom onset and then every 4 months thereafter. Biospecimens including blood and saliva were collected at each visit. CHIRP and LIINC used a harmonized set of case report forms to collect clinical data about demographics, medical history, the COVID-19 illness and post-acute symptoms. Clinical measurements collected during in-person CHIRP visits included complete blood count with differential, comprehensive metabolic panel, erythrocyte sedimentation rate, high-sensitivity C-reactive protein, d-dimer, lactate dehydrogenase and ferritin. All of the participants provided written informed consent agreeing to research and publication of research results, and the CHIRP and LIINC studies were approved by the Institutional Review Board for the University of California, San Francisco (IRB 20-30588 and 20-30479, respectively).

### *HLA* genotyping in the CHIRP/LIINC cohort

A total of 100 ng of high-quality DNA was fragmented using the Library Preparation Enzymatic Fragmentation Kit 2.0 (Twist Bioscience). Subsequently, the ends of the fragmented DNA were repaired, poly(A) tails were added and ligated through PCR to Illumina-compatible dual index adapters that were uniquely barcoded. After ligation, fragments were purified with a 0.8× ratio AMPure XP magnetic beads (Beckman Coulter), followed by dual-size selection (0.42× and 0.15× ratios) to select libraries of approximately 800 bp. Finally, libraries were amplified and purified with magnetic beads.

After fluorometric quantification, 30 ng of each sample was precisely pooled using ultrasonic acoustic energy, and the targeted capture was performed using the Twist Target Enrichment kit (Twist Bioscience). In brief, the volumes were reduced using magnetic beads, and the DNA libraries were bound to 1,394 biotinylated probes specific to the HLA region, covering all exons, introns and regulatory regions of *HLA-A*, *HLA-B*, *HLA-C*, *HLA-DRB1*, *HLA-DRA*, *HLA-DQB1*, *HLA-DQA1*, *HLA-DPB1* and *HLA-DPA1*. Fragments targeted by the probes were captured using Streptavidin magnetic beads and then amplified and purified. Enriched libraries were analysed using the BioAnalyzer (Agilent) and quantified by droplet digital PCR. Finally, enriched libraries were sequenced using the NovaSeq platform (Illumina) with a paired-end 150 bp sequencing protocol. After sequencing, data were analysed using HLA Explorer v.1.4 (Omixon) and AlloSeq Tx V471 (CareDx).

### UK cohort reanalysis

We reanalysed the primary data from a previous study^22^ that analysed HLA class I and class II genes in 147 individuals of European ancestry with known SARS-CoV-2 infection and a range of symptoms and 69 asymptomatic hospital workers. In the initial publication, HLA-B*15:01 was not directly tested for association with asymptomatic disease course. *HLA* genotyping methods, allele frequencies, demographics and clinical outcomes are as previously described.

### *HLA* association analysis

In our discovery cohort, we examined the association of five *HLA* loci (*HLA-A*, *-B*, *-C*, *-DRB1* and *-DQB1*) with asymptomatic versus symptomatic infection. Data analysis included the first two fields of the allele name as described in the HLA nomenclature, representing the complete molecule at polypeptide sequence resolution.

Initial testing for *HLA* associations was performed using the R package BIGDAWG^53^, which handles multiallelic *HLA* data to test for association at the haplotype, locus, allele and amino acid levels. We next used a generalized linear model using glm in the R (v.4.3) base package to consider the relevant covariates, including any reported comorbidities, sex and age, for alleles that were initially found to be associated with asymptomatic infection after correction for multiple testing. We corrected *P* values using the Bonferroni method^54^ for the number of alleles tested at *HLA-A*, *-B* and *-DRB1*, which accounts for the strong linkage disequilibrium between some of the loci tested. For the replication cohorts, we tested only the allele of interest, using the generalized linear model framework as described. Meta-analysis of the results in our three cohorts was performed in R using the common effect model with the meta package (v.6.2-1)^55^.

### Peptide synthesis

The four SARS-CoV-2 peptides (Fig. 1a–c and Supplementary Table 17) were synthesized by in vitro transcription and translation of oligonucleotides encoding each peptide using the PURExpress in vitro protein synthesis kit (New England BioLabs) as previously described^56^. Peptides NQK-Q8 (NQKLIANQF) and NKQ-A8 (NQKLIANAF) (used in Figs. 1d and 2–4) were synthesized using the fluoren-9-ylmethoxycarbonyl (Fmoc) method of solid-phase peptide synthesis. The process was performed using the Biotage Initiator+ Alstra automated peptide synthesizer and Wang resin (100–200 mesh, 1.24 mmol g^−1^), which was swollen in dimethylformamide (DMF) for 2 h before use. The Fmoc-amino acids and HCTU/HOBt/DIPEA (4 eq.) dissolved in DMF were then added sequentially to the resin and the coupling reactions were carried out with microwave heating at 60 °C for 20 min. The Fmoc protecting groups were removed using piperidine (20% in DMF) at room temperature for 20 min. The peptides were cleaved from the resin using a mixture of 95% trifluoroacetic acid (TFA) and 5% triisopropylsilane (TIPS) for 3 h. After evaporation of the TFA/TIPS mixture and precipitation with diethylether, the crude peptides were purified using reversed-phase high-performance liquid chromatography (HPLC) on the Shimadzu HPLC system fitted with two Shimadzu LC-20AD pumps, a SIL-20AHT autosampler, SPD-M20A photodiode array detector and a FRC-10A fraction collector and an Onyx Monolithic C18, 100 × 10 mm semipreparative column with a solvent system consisting of 0.1% TFA in water and acetonitrile over 30 min. The purified peptides were lyophilized and stored at −20 °C. The purity was confirmed to be >95% in each case by analytical HPLC and the structures were confirmed using high-resolution electrospray ionization mass spectrometry (Supplementary Fig. 11).

### PBMCs

A total of 20 unexposed and 1 triple-vaccinated (VAC62) donors were recruited; all details are provided in Supplementary Table 18. PBMCs were separated from whole blood or buffy coats using density-gradient centrifugation. PBMCs were used fresh or were cryogenically stored until use. HLA-genotyped PBMCs from the USA were stored in the National Marrow Donor Program (NMDP)/Be The Match Research Sample Repository (ClinicalTrials.gov protocol NCT04920474) that had been collected from healthy donors before the start of the COVID-19 pandemic. All individuals consented to research and publication of research results and had been previously genotyped for *HLA* class I and class II. Ethics approval to undertake the research was obtained from the QIMR Berghofer Medical Research Institute Human Research Ethics Committee (P2282) and La Trobe University Human Research Ethics Committee (HEC21097). The *HLA* genotyping was performed by AlloSeq Tx17 (CareDx Pty) using AllType NGS high-resolution getyping on the IonTorrent NGS platform.

### TAME

Each peptide was loaded on biotinylated HLA-B*15:01 (either custom-made or purchased from easYmers immunAware) according to the manufacturer’s instructions.

For the combinatorial tetramer staining that included all four SARS-CoV-2 peptides (Supplementary Table 17) related to Fig. 1a–c, peptide-loaded HLA-B*15:01 tetramers were generated using Streptavidin conjugated to PE, APC, PE-CF594 or BV421 according to the manufacturer’s instructions. d-Biotin (500 µM) was subsequently added to each peptide-loaded tetramer and tetramers were pooled just before cell staining. Combinatorial tetramer staining was used to identify each epitope by unique combinations of dual fluorophores where at least one of the fluorophores contained PE (Supplementary Table 17). The frequencies of antigen-specific T cells were calculated as previously described^57^. In brief, an aliquot of PBMCs was used for cell surface staining and counting using 123count eBeads (Invitrogen). The remaining PBMCs were stained with the indicated tetramer pools and enriched using anti-PE magnetic microbeads (Miltenyi) over a magnetic column, cell-surface stained and counted as for pre-enrichment. CD8^+^ T cells were identified by gating live singlet CD8^+^ lymphocytes that were negative for CD4, CD14, CD16 and CD19 (Supplementary Fig. 2 and Supplementary Table 19). A stringent tetramer gating strategy was used whereby CD8^+^ T cells labelled with only two fluorophores were considered antigen specific. The memory status of tetramer-positive CD8^+^ T cells was determined by lack of CCR7 and CD45RA co-expression. Given the low numbers of cells available from donors, only tetramer^+^CD8^+^ T cells with frequencies of greater than 1 × 10^−4^ per total CD8^+^ T cells were considered to be positive.

For the tetramer staining that included only the NQK-Q8 and NQK-A8 peptides (related to Figs. 1d and 2 and Extended Data Fig. 1), peptide-loaded HLA-B*15:01 tetramers were generated using Streptavidin conjugated to phycoerythrin (PE). Tetramer-stained cells were enriched using anti-PE antibody-coated immunomagnetic beads on LS columns (Miltenyi Biotech) according to manufacturer instructions. After enrichment, cells were stained with an antibody panel including anti-CD3-BV480 (dilution 1:100), anti-CD8-PerCP-Cy5.5 (1:50), anti-CD4-BV650 (1:100), anti-CD14-APCH7 (1:200), anti-CD19-APCH7 (1:100), anti-CD45RA-FITC (1:100), anti-CD27-APC (1:100), anti-CCR7-PE-Cy7 (1:50), anti-CD95-BV421 (1:50), anti-PD1-BV605 (1:100) (all BD Biosciences) and Live/Dead Fixable Near-IR Dead Cell Stain (1:1,000) (Life Technologies) (Supplementary Fig. 4). Cells were resuspended in MACS buffer (PBS, 0.5% BSA, 2 mM EDTA) and were directly single-cell index sorted into PCR plates (Eppendorf) using the BD Aria Fusion system.

### Generation of peptide-specific CD8^+^ T cell lines

CD8^+^ T cell lines were generated as previously described^25,58^. In brief, PBMCs were incubated with 1 μM of individual peptide (NQK-Q8 or NQK-A8) and cultured for 10–14 days in RPMI-1640 supplemented with 2 mM MEM non-essential amino acid solution (Sigma-Aldrich), 100 mM HEPES (Sigma-Aldrich), 2 mM l-glutamine (Sigma-Aldrich), penicillin–streptomycin (Life Technologies), 50 mM 2-ME (Sigma-Aldrich) and 10% heat-inactivated fetal bovine serum (Bovogen). The cultures were supplemented with 10 IU IL-2 2–3 times weekly. CD8^+^ T cell lines were used fresh for subsequent analysis. For the double tetramer staining experiments 0.5 × 10^6^ cells from the CD8^+^ T cell lines were stained with a single PE-conjugated tetramer (HLA-B*15:01-NQK-Q8 or HLA-B*15:01-NQK-A8) or double stained with both tetramers (PE-conjugated NQK-A8 and APC-conjugated NQK-Q8 tetramer) for 1 h at room temperature. Cells were washed and surface-stained with anti-CD3-BV480 (dilution 1:100), anti-CD8-PerCP-Cy5.5 (1:50), anti-CD4-BV650 (1:100), anti-CD14-APCH7 (1:200) and anti-CD19-APCH7 (1:100) antibodies (all BD Biosciences) and Live/Dead Fixable Near-IR Dead Cell Stain (Life Technologies). Cells were single-cell sorted into PCR plates (Eppendorf) using the BD Aria Fusion system.

### Intracellular cytokine assay

CD8^+^ T cell lines were stimulated with 1 μM of the cognate or the homologous peptide and were incubated for 4–5 h in the presence of GolgiPlug, GolgiStop and anti-CD107a-FITC (dilution 1:100) (all BD Biosciences). After stimulation, cells were surface stained for 30 min with anti-CD3-BV480 (1:100), anti-CD8-PerCP-Cy5.5 (1:50) and anti-CD4-BV650 (1:100) antibodies (all BD Biosciences) and Live/Dead Fixable Near-IR Dead Cell Stain (Life Technologies). Cells were fixed and permeabilized using BD Cytofix/Cytoperm solution (BD Biosciences) and then intracellularly stained with anti-IFN-γ-BV421 (1:100), anti-TNF-PE-Cy7 (1:100), anti-IL2-PE (1:100) and anti-MIP-1β-APC (1:100) antibodies (all BD Biosciences) for a further 30 min. Cells were acquired on the BD FACSymphony A3 system using the FACSDiva software (v.9.0.). Post-acquisition analysis was performed using FlowJo software (v.10). Cytokine detection levels identified in the no-peptide control condition were subtracted from the corresponding test conditions in all summary graphs to account for non-specific, spontaneous cytokine production.

### Single-cell multiplex PCR

Single-cell multiplex PCR was performed as previously described^59^. In brief, cDNA was generated using the VILO cDNA synthesis kit (Invitrogen) at 1/20 of the manufacturer’s recommendations with 0.1% Triton X-100. Nested PCR comprising 40 α- and 27 β-chains was subsequently undertaken. PCR products were purified using ExoSAP (GE Healthcare) and were sequenced at AGRF. Sequences were analysed using FinchTV (Geospiza v.1.5.0) and IMGT software^60^. CDR3 sequences shown are all productive (no stop codons)^61^.

### SPR analysis

SPR experiments were conducted at 25 °C on the BIAcore T200 instrument in 10 mM Tris-HCl, pH 8.0 (Fisher Bioreagents), 150 mM NaCl (Merck), 0.005% surfactant P20 (Cytiva) and 0.5% BSA (Sigma-Aldrich). Streptavidin chips were used to bind biotinylated HLA-B*15:01-NQK complexes (coupled at ~4,000 response units). The first flow cell was loaded with H2D^b^-PA (negative control). The experiments were conducted with ten serial dilutions of the TCRs starting at 100 or 150 μM. All of the experiments were conducted in duplicate twice (*n* = 2 independent experiment). BIAevaluation (v.3.1) and GraphPad Prism 9 (v.9.3) were used for data analysis reported in Supplementary Table 10.

### Analysis of the TCR clonotypic repertoire

TRA and TRB sequences were analysed using the software suite from the International ImMunoGeneTics (IMGT) Information System^62^. The V(D)J gene nomenclature used is that of the IMGT database (www.imgt.org). Motifs enriched were identified using the MEME suite motif discovery software (v.5.5.2.)^63^. The MEME software chooses the width and number of occurrences of each motif automatically to minimize the *E*-value of the motif. Motifs were searched in discriminative mode and were represented as sequence logos, where the relative sizes of the letters indicate their frequencies in the sequence set, and the total height of the letters represents the information content of the position, in bits.

### Statistical analysis

GraphPad Prism 9 (v.9.3) was used to perform statistical analysis. Statistical comparisons between groups were determined using one-way or two-way analysis of variance with correction for multiple comparisons. *P* < 0.05 was considered to be significant.

### Protein expression, refold and purification

HLA-B*15:01 heavy chain, β2-microglobulin and TCR α- and β-chains were expressed in *BL21 Escherichia coli* cells as inclusion bodies. HLA-B*15:01 was refolded and purified as described previously^61^. In brief, soluble HLA-B*15:01–NQK-Q8 or -A8 complexes were produced by refolding inclusion bodies at the following amounts: 30 mg of heavy chain, 10 mg of β2-microglobulin and 5 mg of the peptide into 200 ml of buffer (100 mM Tris-HCl pH 8.0 (Fisher Bioreagents), 400 mM l-arginine (Sigma-Aldrich), 500 µM glutathione oxidized (Goldbio), 5 mM glutathione reduced (Goldbio), 20 mM EDTA (Sigma-Aldrich)). The *TRBV7-2* gene has four alleles (TRBV7-2*01 and TRBV7-2*02/03/04), changing the Ser45 to Arg45 upstream of the CDR2β loop and Gly84 to Glu84 of the HV4 loop^64^ that could impact pHLA recognition. From the TCR sequencing results, we could not differentiate the alleles. As TRBV7-2*02/03/04 are similar at the protein level, we produced the TCRs with either TRBV7-2*01 or TRBV7-2*02. The SPR results show that the polymorphism within the β-chain did not impact the pHLA recognition (Extended Data Figs. 7 and 8 and Supplementary Table 10). TCRs were refolded by mixing 100 mg of the α-chain and 50 mg of the β-chain in 400 ml of the same buffer containing 5 M urea (Sigma-Aldrich). The refold mixtures were dialysed into 10 mM Tris-HCl pH 8.0 (Fisher Bioreagents). The HLA-B*15:01–NQK complexes and the TCRs were purified using anion-exchange chromatography (DEAE and HiTrap Q, both GE Heathcare).

### DSF analysis

Thermal stability assays were performed by DSF using the ViiA 7 real-time PCR system (Thermo Fisher Scientific), in which the HLA-B*15:01–YFP complex was heated from 25 to 95 °C at a rate of 1 °C min^−1^ in 0.5 °C steps. The excitation and emission channels were set to the TAMRA reporter (x3m3 filter) with excitation of ~550 nm and detection at ~587 nm. The experiment was performed at two concentrations of pHLA (5 µM and 10 µM) in duplicates. Each sample was dialysed in 10 mM Tris-HCl pH 8.0 (Fisher Bioreagents), 150 mM NaCl (Merck) and contained a final concentration of 10× SYPRO Orange Dye (Invitrogen). Fluorescence intensity data were normalized and plotted using GraphPad Prism 9 (v.9.3). The *T*_m_ value for a pHLA is equal to the temperature at which 50% of maximum fluorescence intensity is reached, which is equal to approximately 50% of unfolded protein and is summarized in Supplementary Table 9.

### Crystallization and structure determination

Crystals of HLA-B*15:01–peptide complexes were grown by hanging-drop vapour diffusion at 20 °C. The protein:reservoir drop ratio was 1:1, at a concentration of 3 mg ml^−1^ in 10 mM Tris-HCl pH 8 (Fisher Bioreagents), 150 mM NaCl (Merck). Crystals of HLA-B*15:01–NQK-Q8 were grown in 0.2 M sodium formate pH 7.0 and 20% (w/v) polyethylene glycol 3350; and, for HLA-B*15:01–NQK-A8, in 20% (w/v) polyethylene glycol 3350 and 2% (v/v) ethylene glycol. Protein crystals were soaked in a cryoprotectant solution containing mother liquor solution with 20% (v/v) ethylene glycol and then flash-frozen in liquid nitrogen. The data were collected on the MX2 beamline at the Australian Synchrotron, part of ANSTO, Australia^65^. The data were processed using XDS^66^ and the structures were determined by molecular replacement using the PHASER program (v.2.8.3)^67^ from the CCP4 suite^68^ with a model of HLA-B*15:01 without the peptide (derived from Protein Data Bank (PDB) 5TXS)^69^. Manual model building was conducted using COOT^70^ followed by refinement with BUSTER^71^ and PHENIX (v.1.20.1-4487)^72^. The final models have been validated and deposited using the wwPDB OneDep System and the final refinement statistics and PDB codes are summarized in Extended Data Table 2. All molecular graphic representations were created using PyMOL (v.2.5).

### Reporting summary

Further information on research design is available in the Nature Portfolio Reporting Summary linked to this article.

## Online content

Any methods, additional references, Nature Portfolio reporting summaries, source data, extended data, supplementary information, acknowledgements, peer review information; details of author contributions and competing interests; and statements of data and code availability are available at 10.1038/s41586-023-06331-x.

### Supplementary information

Supplementary Figures This file contains Supplementary Figs. 1–11.

Reporting Summary

Supplementary Tables This file contains Supplementary Tables 1–19.

## Figures and Tables

**Extended Data Table 1:**
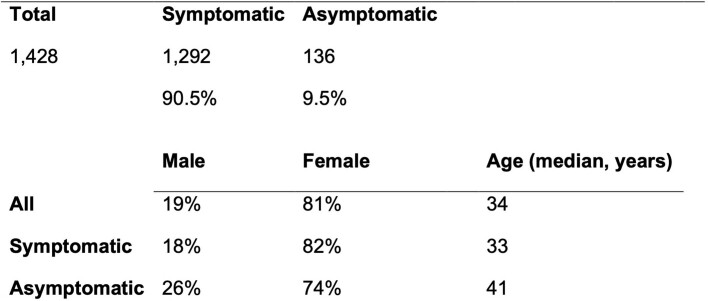
Study population demographics

Among these respondents, 136 (9.5%) reported having remained asymptomatic for at least two weeks before and after a positive test for the virus. The median age was increased in asymptomatic compared to symptomatic individuals (asymptomatic = 41, symptomatic = 33, p < 0.001), and we observed increased reporting for asymptomatic infection in males (p = 0.02). We also collected data on several diseases and conditions that might impact COVID-19 disease course. Among all individuals who reported a positive test for the virus (n = 1,428), 67% reported no known COVID-19-associated comorbidities.

**Extended Data Table 2:**
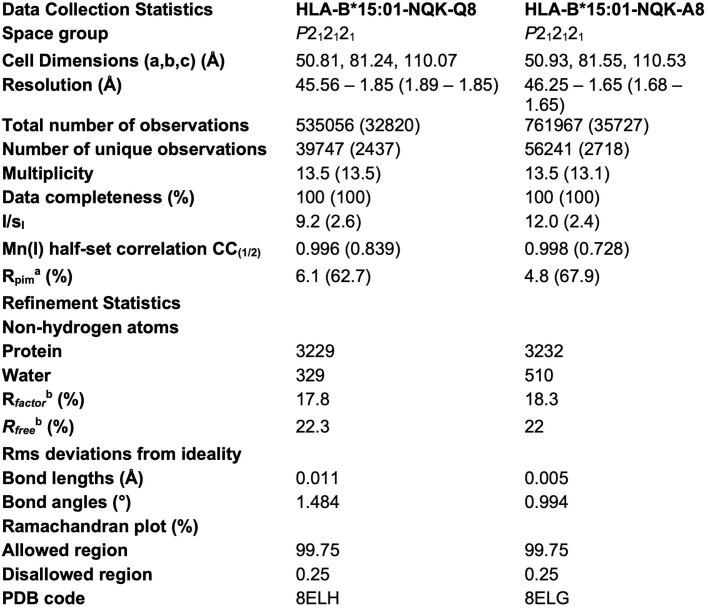
Data Collection and Refinement Statistics

^a^R_p.i.m_ = S_hkl_ [1/(N-1)]^1/2^ S_i_ | I_hkl, i_ - < I_hkl_ > | / S_hkl_ < I_hkl_ >

^b^R_factor_ = S_hkl_ | | F_o_ | - | F_c_ | | / S_hkl_ | F_o_ | for all data except ≈ 5% which were used for R_free_ calculation.

Values in parentheses are for highest-resolution shell.

## Data Availability

All HLA and phenotypic data for the Citizen Science and CHIRP/LIINC and UK cohorts are available in the online public database (http://www.hlacovid19.org/database/; Project 3 Hollenbach and Project 6 Langton). The crystallography structures are available at the PDB under accession codes 8ELG and 8ELH for HLA-B*15:01–NQK-A8 and HLA-B*15:01–Q8, respectively.
