# Supplementary material for: A common allele of HLA is associated with asymptomatic SARS-CoV-2 infection
Source: Nature. 2023 Jul 19;620(7972):128–36. doi: 10.1038/s41586-023-06331-x (PMC10396966; doi:10.1038/s41586-023-06331-x)
Supplement: Supplementary file 1 — This file contains Supplementary Figs. 1–11. [file 41586_2023_6331_MOESM1_ESM.docx]

**Supplementary Figures for**

**A common allele of *HLA* is associated with asymptomatic SARS-CoV-2 infection**

Danillo G. Augusto^1,2,3 *^, Lawton D. Murdolo^4 *^, Demetra S.M. Chatzileontiadou^4,5 *^, Joseph J. Sabatino, Jr.^1^, Tasneem Yusufali^1^, Noah D. Peyser^6^, Xochitl Butcher^6^, Kerry Kizer^1^, Karoline Guthrie^1^, Victoria Murray^7^, Vivian Pae^7^, Sannidhi Sarvadhavabhatla^7^, Fiona Beltran^7^, Gurjot Gill^7^, Kara Lynch^8^, Cassandra Yun^8^, Colin Maguire^9^, Michael J. Peluso^7^, Rebecca Hoh^7^, Timothy J. Henrich^10^, Steven G. Deeks^7^, Michelle Davidson^11^, Scott Lu^12^, Sarah A. Goldberg^12^, J. Daniel Kelly^12,13^, Jeffrey N. Martin^12^, Cynthia A. Viera-Green^14^, Stephen R. Spellman^14^, David J. Langton^15^, Michael J. Dewar-Oldis^4^, Corey Smith^16^, Peter J. Barnard^4^, Sulggi Lee^7^, Gregory M. Marcus^6^, Jeffrey E. Olgin^6^, Mark J. Pletcher^12,17^, Martin Maiers^18^, Stephanie Gras^4,5 **^, Jill A. Hollenbach^1,12 **^

1. Weill Institute for Neurosciences, Department of Neurology, University of California San Francisco, San Francisco, CA, USA

2. Department of Biological Sciences, The University of North Carolina at Charlotte, Charlotte, NC, USA

3. Programa de Pós-Graduação em Genética, Universidade Federal do Paraná, Curitiba, Brazil

4. Department of Biochemistry and Chemistry, La Trobe Institute for Molecular Science, La Trobe University, Bundoora, Victoria 3086, Australia.

5. Department of Biochemistry and Molecular Biology, Biomedicine Discovery Institute, Monash University, Clayton, Victoria 3800, Australia.

6.Division of Cardiology, Department of Medicine, University of California San Francisco, San Francisco, CA, USA

7. Division of HIV, Infectious Diseases, and Global Medicine, Department of Medicine, University of California San Francisco, San Francisco, CA, USA

8. Department of Laboratory Medicine, University of California San Francisco, San Francisco, CA, USA

9. University of Utah, Clinical and Translational Science Institute, Salt Lake City, UT

10. Division of Experimental Medicine, Department of Medicine, University of California San Francisco, San Francisco, CA, USA

11. Department of Medicine, University of California San Francisco, San Francisco, CA, USA

12. Department of Epidemiology and Biostatistics, University of California San Francisco, San Francisco, CA, USA

13. F.I. Proctor Foundation, University of California San Francisco, San Francisco, CA, USA

14. CIBMTR (Center for International Blood and Marrow Transplant Research), National Marrow Donor Program/Be The Match, Minneapolis, Minnesota.

15. ExplantLab, The Biosphere, Newcastle Helix, Newcastle-upon-Tyne, UK

16. QIMR Berghofer Medical Research Institute - QIMR Berghofer Centre for Immunotherapy and Vaccine Development Brisbane, Queensland, Australia

17. Division of General Internal Medicine, University of California San Francisco, San Francisco, CA, USA

18. National Marrow Donor Program, Minneapolis, MN

* These authors contributed equally to this work
 ** These authors contributed equally to this work

**Corresponding Author:**

**Jill A. Hollenbach, PhD, MPH**
 University of California, San Francisco

Department of Neurology and Department of Epidemiology and Biostatistics

675 Nelson Rising Ln, 221A
 San Francisco, CA 94158

[jill.hollenbach@ucsf.edu](mailto:jill.hollenbach@ucsf.edu)

**Contents**

[Supplementary Figure S1. Meta-analysis across discovery and replication cohorts 3](#_Toc136625719)

[Supplementary Figure S2. Gating overview for flow cytometry analysis, related to Figure 1a-c 4](#_Toc136625720)

[Supplementary Figure S3. *Ex vivo* tetramer staining following tetramer magnetic enrichment, related to Figures 1a-c 5](#_Toc136625721)

[Supplementary Figure S4. Gating strategy, related to Figures 1d and 2a-c and Extended Data Fig. 1 6](#_Toc136625722)

[Supplementary Figure S5. T cell cross-reactivity of NQK-Q8 and NQK-A8 specific T cell lines, related to Figure 2b 7](#_Toc136625723)

[Supplementary Figure S6. NQK-Q8-specific TRAV and TRBV usage in unexposed, COVID-19 recovered and vaccinated donors 8](#_Toc136625724)

[Supplementary Figure S7. NQK-specific TCR repertoire CDR3 analysis in COVID-19 recovered and vaccinated donors 9](#_Toc136625725)

[Supplementary Figure S8. Amino acid alignments among coronaviruses proteins 10](#_Toc136625726)

[Supplementary Figure S9. Discovery cohort inclusion criteria 21](#_Toc136625727)

[Supplementary Figure S10. Definition of asymptomatic disease course in the discovery cohort 22](#_Toc136625728)

[Supplementary Figure S11. Peptide characterization 23](#_Toc136625729)

[Reference 24](#_Toc136625730)

# Supplementary Figure S1. Meta-analysis across discovery and replication cohorts

Meta-analysis p-value < 10^-4^. OR = odds ratio; CI – confidence interval. The size of the shaded square corresponds to the weight given in the meta-analysis. Horizontal bars indicate confidence intervals for OR. Citizen Science cohort n=1428; UK cohort n=156; CHIRP/LIINC cohort n=82.

# Supplementary Figure S2. Gating overview for flow cytometry analysis, related to Figure 1a-c


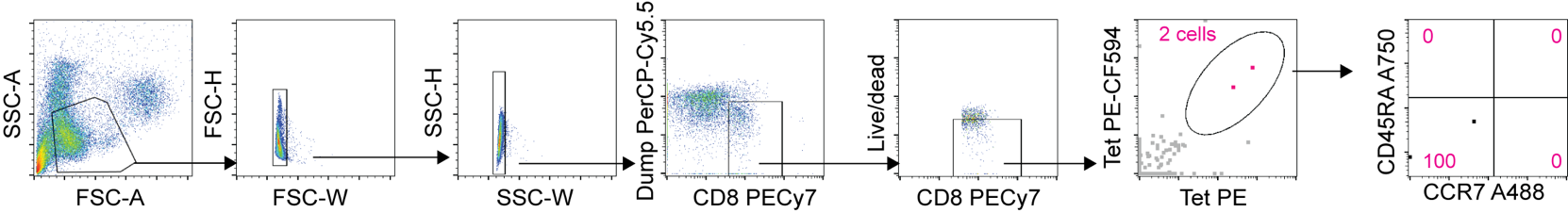


Schematic of the gating strategy following tetramer magnetic enrichment. CD8^+^ T cells were identified by live single cell lymphocytes that were dump antibody negative (CD4/CD14/CD16/CD19). CD8^+^ T cells double tetramer-positive in PE and PE-CF594 (and negative for the remaining two fluorophores APC and BV421) were gated to ensure specificity of tetramer binding. The resulting cells were considered NQKLIANQF-specific and further gated on CCR7 and CD45RA to determine memory status.

# Supplementary Figure S3. *Ex vivo* tetramer staining following tetramer magnetic enrichment, related to Figures 1a-c

CD8^+^ T cells specific for each of the four indicated SARS-CoV-2 peptides were identified through a unique combination of double tetramer-positivity in two fluorophores (and negative in the two remaining fluorophores) for binding specificity. The resulting CD8^+^ T cells were considered antigen-specific (red dots) and further gated on CCR7 and CD45RA to determine memory status. It should be noted that although the specificity for antigen-specificity is considered high, the detection sensitivity was limited in these samples due to cell viability and low amounts of available PBMCs.

# Supplementary Figure S4. Gating strategy, related to Figures 1d and 2a-c and Extended Data Fig. 1

**
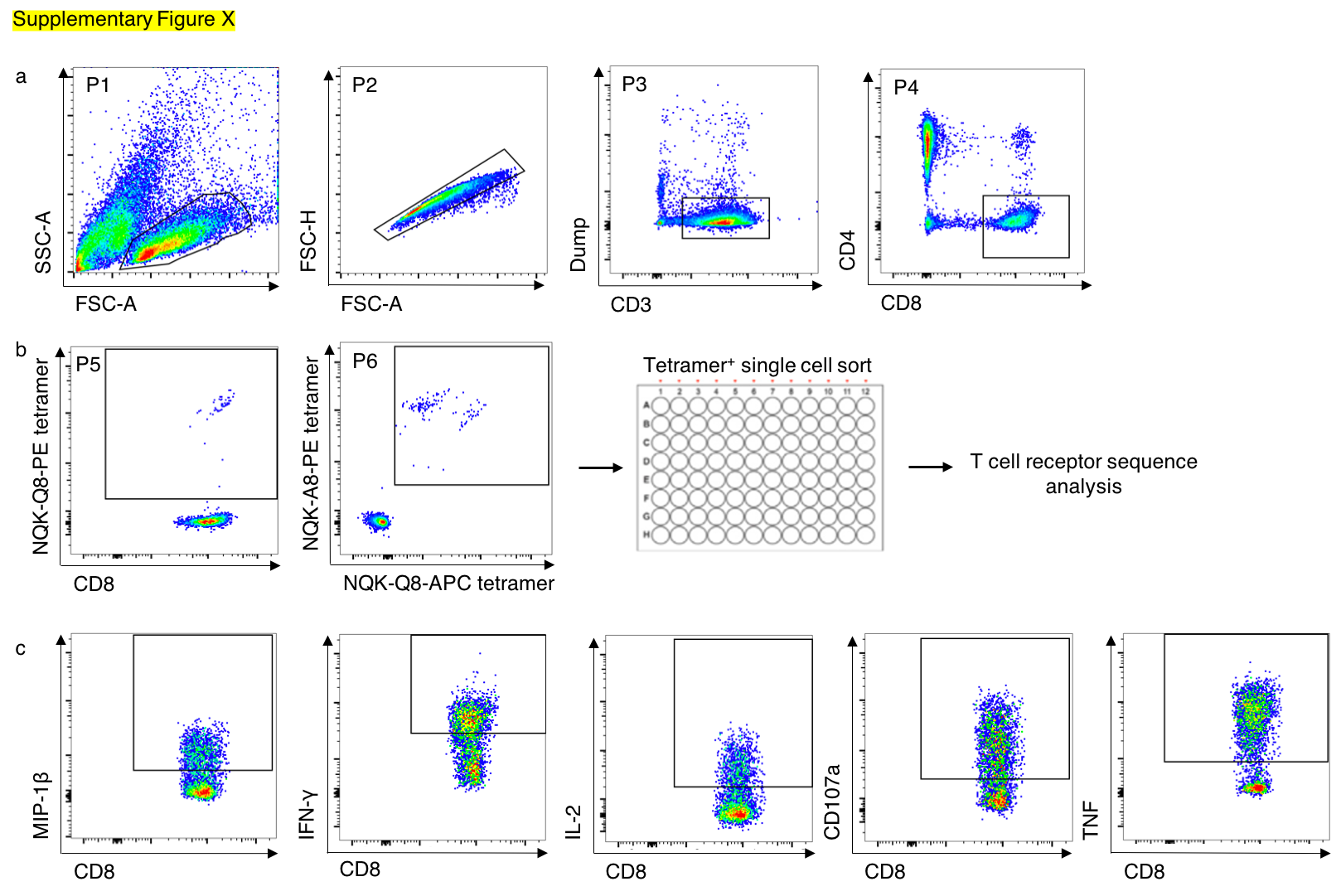
**

(**a**) PBMC or HLA-B*15:01-NQK specific CD8^+^ T cell lines gating strategy. CD8^+^ T cells were single cell sorted based on their tetramer specificity (**b**) or were assessed by function in an ICS assay (**c**). (**b**) Schematic displaying representative tetramer staining for single-cell isolation and TCR sequencing of HLA-B*15:01-NQK-Q8 or -A8 or both tetramer^+^ cells. (**c**) Representative gating for the assessment of polyfunctionality from an ICS assay.

# Supplementary Figure S5. T cell cross-reactivity of NQK-Q8 and NQK-A8 specific T cell lines, related to Figure 2b

*In vitro* tetramer analysis for the NQK-A8- (left panels, in orange) and NQK-Q8- (right panels, in purple) specific T cell lines in 5 donors. FACS plots of each donor that show either single tetramer staining with either the Tet-Q8 and Tet-A8 tetramer on PE conjugate or double tetramer staining with both Tet-Q8 and Tet-A8 tetramers on APC conjugate and PE conjugate, respectively.

# Supplementary Figure S6. NQK-Q8-specific TRAV and TRBV usage in unexposed, COVID-19 recovered and vaccinated donors


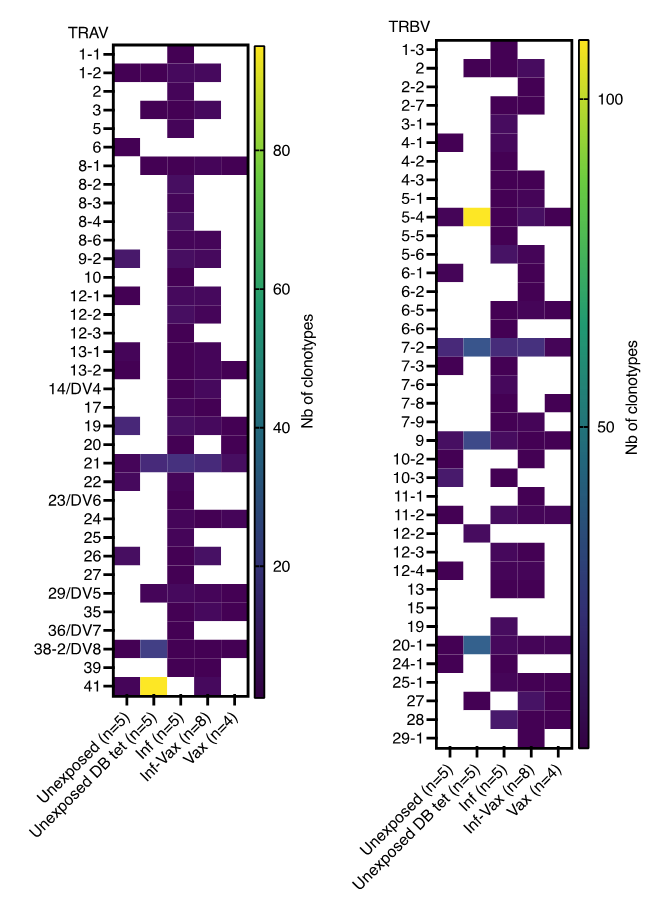


Heatmaps displaying preferred TRAV (left) and TRBV (right) usage of NQK-Q8- or both peptide-specific TCRs in unexposed donors; and NQK-Q8-specific TCRs in COVID-19 recovered (Inf), in COVID-19 recovered and vaccinated (Inf-Vax) and in COVID-19 vaccinated (Vax) (Minervina et al., 2022).^1^

# Supplementary Figure S7. NQK-specific TCR repertoire CDR3 analysis in COVID-19 recovered and vaccinated donors


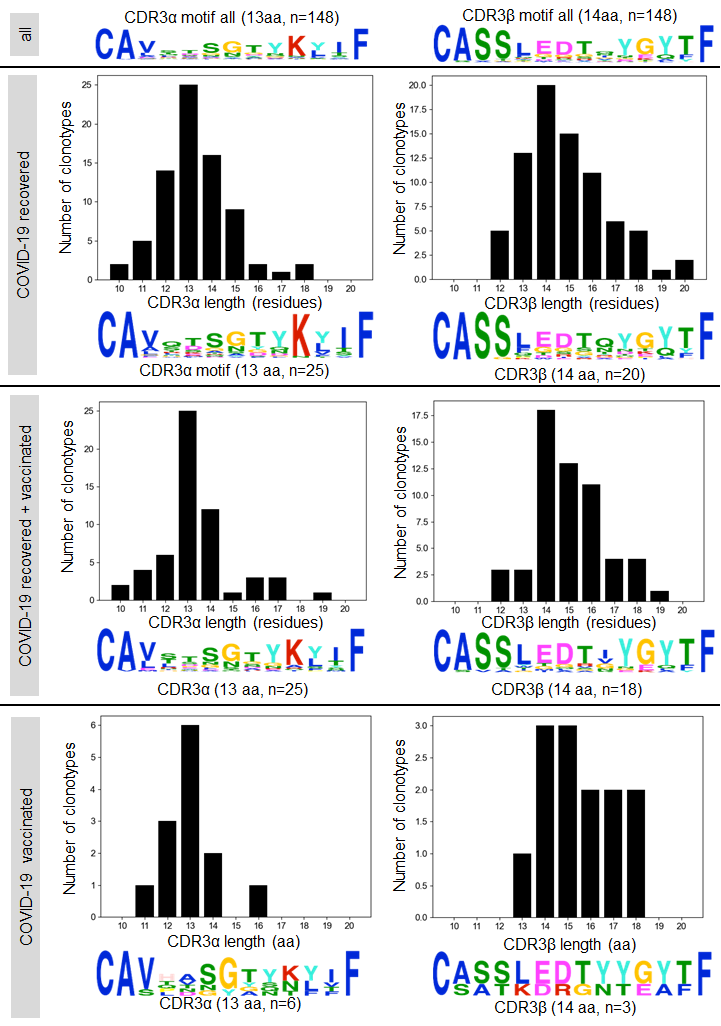


Summary of CDR3α (left) and CDR3β (right) lengths and motif analysis for the NQK-Q8-specific TCR clonotypes in COVID-19 recovered and vaccinated from published data (Minervina et al., 2022)^1^. The MEME motif discovery program was used to identify aa motifs enriched; the relative size of each residue symbol is proportional to its frequency, while the total height of aa symbols indicates the information content of the position in bits. The motif on top left panel is the CDR3α 13 aa long sequence motif derived from all CDR3α sequences obtained (n=148); on the right top panel is the CDR3β 14 aa long sequence motif derived from all CDR3β sequences obtained (n=148).

# Supplementary Figure S8. Amino acid alignments among coronaviruses proteins

**a**

Consensus AMQMAYRFNGIGVTQNVLYE**NQKLIANQF**NSAIGKIQDSLSSTASALGKLQDVVNQNAQALNTLVKQLSSNFGAISSVLNDILSRLDKVEAEVQIDRLIT

Omicron ....................**.........**..........................H..............K...........F....PP...........

Delta ....................**.........**......................N................................................

Alpha ....................**.........**......................................................A...PP...........

Beta ....................**.........**.......................................................................

Gamma ....................**.........**..........................................................PP...........

Lambda ....................**.........**.......................................................................

Mu ....................**.........**.......................................................................

Epsilon ....................**.........**.......................................................................

Zeta ....................**.........**.......................................................................

Eta ....................**.........**.......................................................................

Iota ....................**.........**.......................................................................

Kappa P...................**.........**..............P...........................................PP...........

**b**

**Spike glycoprotein**

10 20 30 40 50 60 70 80 90 100

SARS-CoV-2 --MFVFLVLLPLVSS-----QCVNLT-------TRT-QLPPAY----TNS---------------FTRGVYYP-DKV------------------F---R

HKU1 --..LIIFI..TTLAVIGDFN.T.S-----FINHYNKTI.RISEDVVDV.---------------LGL.T..VLNR.YLNTSLLFTGYFPKSGAN.---.

OC43 -MFLIL.IS..TAFAVIGDLN.PLDPKLKGSFNN.DTGS.SISTDTVDVT---------------NGL.T..VL.R.YLNTTLFLNGYYPTSGSTY---.

229E ----------------------------------------------------------------------------------------------------

NL63 MKL.LI.LV...A.CFF---T.NSNANL----SMLQLGV.DNSSTIV.GLLPTHWFCANQSTSVYSAN.FF.IDVGNHRSAFALHTGYYDANQYYIYVTN

110 120 130 140 150 160 170 180 190 200

SARS-CoV-2 SSVLHSTQDLFLPFFSNVTWFHAIHVSGTN-GTKRFDNPVLPFNDGVYFASTEKSNIIRGWIFGTTLDS-KTQSLLI-------V---------------

HKU1 DLA.KGS------TYLSTL.YKPPFL.DF.N.IFSKVKNTKLYVNNTLYSE--F.T.VI.SV.VN.SYTIVV.PH-------------------------

OC43 NMA.KG.------DLLSTL..KPPFL.DFIN.IFAKVKNTKV.K...MYSE--FPA.TI.ST.VN.SY.VVV.PRT.NSTQDGVN---------------

229E ---------------------------------------------------------------------------------MFVLLVAY-----------

NL63 EIG.NASVT.KICK..RN.T.--DFL.NAS---SS..-----CIVNLL.TEQ------L.APL.I.ISGETVRLH.YNVTRTFY.PAAYKLTKLSVKCYF

210 220 230 240 250 260 270 280 290 300

SARS-CoV-2 ------NNATNVVIKVCEF-Q**FCNDPFLGVY**YH---KNNKSWMESEFRVYSS-----------------ANNCTFEYVSQPFLMDLEGKQGNFKNLREFV

HKU1 --------NGILE.TA.QY-T**M.EY.HTVCK**SK-GSIR.E..H-----ID..-----------------EPL.L.KK---N.TYNVSAD------WLY.H

OC43 ------KLQGLLEVS..QY-N**M.EH.HTICH**PNLGNHFKEL.H-----LDTG-----------------VVS.LYKR---N.TY.VNAT------YLY.H

229E ------A---LLH.AG.QTTN**GT.TSHSVCN**GCVGHSE.VFDV..GGYIP.NFAFNNWFLLTNTSSVVDGVVRS.QPLLLNC.WSVS.S.V-ITGFVY.N

NL63 NYSCVFSVVNAT.TVNVTTHN**GRVVNYTVCD**DCNGYTD.IFSVQQDG.IPNGFPFNNWFLLTNGSTLVDGVSRLYQPLRLTC.WPVP.LKS-STGFVY.N

310 320 330 340 350 360 370 380 390 400

SARS-CoV-2 FKNIDGYFKIYSKHTPINLVRDLPQGFSALEPLVDLPIGINITRFQTLLALHRSYLTPGD---SSSG----------------------W----------

HKU1 .YQER.V.YA.YADVG--M----.T-----TF.FS.YL.TILSH---------Y.VM.LTCNAI.PK----------------------I----------

OC43 .YQEG.T.YA.FTD.G--F----VT-----KF.FNVYL.MALSH---------Y.VM.LTCIS--RR----------------------D----------

229E GTGRG-AC.GFYSNASSDVI.Y-NIN.EE-----N.RR.TILFK--.-----SYGAVVFYCTNNTLVSGDAHIPSGTVLGNFYCFVNTTIGNETTSAFVG

NL63 ATGS.VNCNG.QHNSVVDVM.Y-NLN...-NS.DN.KS.VIVFK--.-----LQ.DVLFYCSN....VLDTTIPFGPSSQPYYCFINSTINTTHVSTFVG

410 420 430 440 450 460 470 480 490 500

SARS-CoV-2 --------------------------------------TAGAAAYYVGYLQPRTFLLKYNENGTITDAVDCALDPLSETKCTLKSFTVEKGIYQTSNFRV

HKU1 --------------------------------------DNETLE.W.TP.SR.QY..NFD.H.V..N....SSSF...IQ.KTQ..APNT.V.DL.G.T.

OC43 --------------------------------------IGFTLE.W.TP.T..QY..AF.QD.I.FN....MS.FM..I..KTQ.IAPPT.V.ELNGYT.

229E ALPKTVREFVISRTGHFYINGYRYFSLGDVEAVNFNVTN.ATTDFCTVA.ASYADV.VNVSQTA.ANIIY.N-SVINRLR.DQL..D.PDVF.S..PIQP

NL63 ILPPTVREIVVARTGQFYINGFKYFDLGFIEAVNFNVT..S.TDFWTVAFATFVDV.VNVSATN.QNLLY.D-S.FEKLQ.EHLQ.GLQD.F.SANFLDD

510 520 530 540 550 560 570 580 590 600

SARS-CoV-2 QPTESIVRFPNITNLCPFGEVFNATRFASVYAWNRKRISN**CVADYSVLY**NSASFSTFKCYGVSPTKLNDLCFTNVYADSFVIRGDEVRQIAPGQTGKIAD

HKU1 K.VATVY.RIPNLPD.DIDNWL.NVSVP.PLN.E.RIF..**.NFNL.T.L**RLVHVDS.S.NNLDKS.IFGS..NSITV.K.A.PNRRRDDLQL.SS.FLQS

OC43 ..IADVY.RKPDLPN.NIEAWL.DKSVP.PLN.E..TF..**.NFNM.S.M**SFIQADS.T.NNIDAA.IYGM..SSITI.K.A.PNRRKVDLQL.NL.YLQS

229E VELPESIVSLPVY-------------------HK-HTFIV**LH----.KF**E-HGPGPG...NCR.AVI.------ITLAN.NET---KGPLCVDTSHFTTK

NL63 NVLPETYVALP.Y-------------------YQ-HTDI.**FT----ATA**----SFGGS..VCK.HQV.------ISLNG-------NTSVCVRTSHFSIR

610 620 630 640 650 660 670 680 690 700

SARS-CoV-2 YNYKLPDDFTGCVIAWNSNNLDSKVG----GNYNYLYRLFRKSNLKP**FERD--------ISTEIY**QAGS--TPC---------NG-----------VEGF

HKU1 S...IDISSSS.QLYYSLPLPNVTINNFNPSSW.RR.GFGSFNV---**------SSYDVVY.DHCF**SVN.DFC..ADPSVVNSCDKSKPLSAICPTGTKYR

OC43 S..RIDTTA.S.QLYY.LPAANVS.SRFNPSTW.KRFGFIED.VFV.**QPTGVFTNHSVVYAQHCF**K.PKNFC..SS------CP.KNNGIGTCPAGTNSL

229E .VAV-----------------YA-----NV.RWSA------------**------------------**------------------------------SINTG

NL63 .I.N-----------------RV.S.SPGDSSWHI------------**------------------**------------------------------YLKSG

710 720 730 740 750 760 770 780 790 800

SARS-CoV-2 NCYF-----------------PLQSY-------------------------------------------G**FQPTNGVGY**QPY------RVVVLSFELLHA

HKU1 H.DLDTTLYVKNWCRCSCLPD.IST.--SPNTCPQKKVVVGIGEHCPGLGINEEKCGTQLNH----SSCS**CS.DAFL.W**SFDSCISNN.CNIF.NFIFNG

OC43 T.DN------------LCTLD.ITFKAPDTYKCPQTKSLVGIGEHCSGLAVKSDYCGN--------NSCT**C..QAFL.W**SADSCLQGDKCNIFANFI..D

229E ..P.-------------------S--------FGKVNNFVKFGSVCFSLKAIPGGCAMPIMANLVNYKSH**NIGSLY.SW**SDGDVITG-------------

NL63 T.P.-------------------S--------FSKLNNFQKFKTICFSTVEVPGSCNFPLEATWHYTSYT**IVGALY.TW**SEGNSITG-------------

810 820 830 840 850 860 870 880 890 900

SARS-CoV-2 --PATVCG---PKKSTN**LVKNKCVNF**NFNGLTGTG**VLTESNKKF**LP-FQQFGRDIADTTDAVRDPQTLEILDITPCSFGGVSVITPGTNTSNQVAVLYQD

HKU1 INSG.T.SNDLLYSN.E**VSTGV...Y**DLY.I..Q.**IFK.VSAAY**YNNW.NLLY.SNGNIIGFK.FL.NKTYT.L..YS.R..AAFY--QN.SSP.L..RN

OC43 VNNGLT.STDLQ.AN.E**IELGV...Y**DLY.IS.Q.**IFV.V.ATY**YNSW.NLLY.SNGNLYGF..YI.NRTFM.HS.YS.R..AAYH--AN.PEP.L.FRN

229E ----VPKPVEGVSSFM.**VTL...TKY**.IYDVS.V.**.IRI..DT.**.NGITY--TSTSGNLLGFK.VTNGT.YS....NPPDQL.VYQQAVVG---.M.SEN

NL63 ----VPYPVSGIREFS.**..L.N.TKY**.IYDYV...**IIRS..QSL**AGGITY--VSNSGNLLGFKNVS.GN.FIV...NQPDQVAVYQQSIIG---.MTAVN

910 920 930 940 950 960 970 980 990 1000

SARS-CoV-2 VNCTEVPVAIHADQLTPTW**RVYSTGSNVF**QTRAGCLIGAEHVN--NSYECDI----PIGAGICASYQTQTNSPRRARS**VASQSIIA---Y**TMSLG-AENS

HKU1 LK.SY.LNN.S---FISQ-**------PFY.**DSYL..VLN.VNLTSYSVSS..L----RM.S.F.ID.ALPSSRRK.RGI**SSPYRFVTFEPF**NV.FVNDSVE

OC43 IK.NY.FNNSLTR..Q.I-**------NYS.**DSYL..VVN.YNSTAISVQT..L----TV.S.Y.VD.SK--.RRS.RAI**TTGYRFTNFEPF**.VNSVNDSLE

229E -FTSYGFSNVV---EM.--**------KFFY**ASNG-------------T.N.TDAVLTYSSF.V..DGSIIAVQ..N---**------------**-V.YDSV---

NL63 -ESRYGLQNLL---QL.--**------NFYY**VSNG-------------GNN.TTAVMTYSNF....DGSLIPVR..N---**------------**-S.DNGI---

1010 1020 1030 1040 1050 1060 1070 1080 1090 1100

SARS-CoV-2 VAYSNNSIAIPTNFTISVTTEILPVSMTKTSVDCTMYICGDSTECSNLLLQYGSFCTQLNRALTGIAVEQDKNTQEVFAQVK-Q-----IYKTPPIKDFG

HKU1 TVGGLFE.Q.......AGHE.FIQT.SP.VTI..SAFV.SNYAA.HD..SE..T..DNI.NI.NEVNDLL.ITQLQ.ANALMQGVTLSSNVN.NLHS.VD

OC43 PVGGLYE.Q..SE...GNME.FIQT.SP.VTI..AAFV...YAA.KLQ.VE.....DNI.AI..EVNELL.TTQLQ.ANSLMNGVTLSTKL.DGVNFNVD

229E S.IVTANLS..S.W.T..QV.Y.QITS.PIV...ST.V.NGNVR.VE..K..T.A.KTIED..RNS.MLESADVS.MLTFD.------KAFTLANVSS..

NL63 S.IITANLS..S.W.T..QV.Y.QITS.PIV...AT.V.NGNPR.K...K..T.A.KTIED..RLS.HLETNDVSSMLTFDS------NAFSLANVTS..

1110 1120 1130 1140 1150 1160 1170 1180 1190 1200

SARS-CoV-2 GFNFSQIL----PDPSKPSKRSFIEDLLFNKVTLADAGFIK-QYGDCLGDIAARDLICAQKFNGLTVLPPLLTDEMIAQYTSALLAGTITSGWTFGAGAA

HKU1 NID.KSL.GCLGSQCGS-.S..LL........K.S.V..VE-A.NN.T.GSEI...L.V.S...IK....I.SETQ.SG..T.ATVAAMFPP.SAA..--

OC43 DI...PV.GCLGSEC..A.S..A......D..K.S.V..VE-A.NN.T.GAEI.....V.SYK.IK......SENQ.SG..L.ATSASLFPP..AA..--

229E DY.L.SVIPSLPRSG.RVAG..A...I..S.LVTSGL.TVDAD.KK.TKGLSIA..A...YY..IM...GVADA.RM.M..GS.IG.IALG.L.SA.S--

NL63 DY.L.SV.PQRNIRS.RIAG..AL.....S..VTSGL.TVDVD.KS.TKGLSIA..A...YY..IM...GVADA.RM.M..GS.IG.MVLG.L.SA.A--

1210 1220 1230 1240 1250 1260 1270 1280 1290 1300

SARS-CoV-2 LQIPFAMQMAYRFNGIGVTQNVLYE**NQKLIANQF**NSAIGKIQDSLSSTA--------------SALGKLQDVVNQNAQALNTLVKQLSSNFGAISSVLND

HKU1 --V..SLNVQ..I..L...MD..NK**.......A.**.K.LLS..NGFTA.N--------------...A.I.S...A......S.LQ..FNK......S.QE

OC43 --V..YLNVQ..I..L...MD..SQ**.......A.**.N.LHA..QGFDA.N--------------...V.I.A...A.SE...N.LQ...NR.....AS.QE

229E --...SLAIQS.L.YVALQTD..Q.**..RIL.AS.**.K.MTN.V.AFTGVNDAITQTSQALQTVAT..N.I......QGNS.SH.TS..RQ..Q....SIQA

NL63 --...SLALQA.L.YVALQTD..Q.**...IL.AS.**.K..NN.VA.F..VNDAITQTAEAIHTVTI..N.I......QGS...H.TS..RH..Q...NSIQA

1310 1320 1330 1340 1350 1360 1370 1380 1390 1400

SARS-CoV-2 ILSRLDKVEAEVQIDRLITG**RLQSLQTY**VTQQLIRAAEIRASANLAATKMSECVLGQSKRVDFCGKGYHLMSFPQSAPHGVVFLHVTYVPAQEKNFTTAP

HKU1 ......NL..Q.......N.**..TA.NA.**.S...SDITL.K.G.SR.IE.VN...KS..P.IN...N.N.IL.LV.N..Y.LL.I.FS.K.TSF.TVLVS.

OC43 ......AL...A......N.**..TA.NA.**.S...SDSTLVKF..AQ.ME.VN...KS..S.IN...N.N.II.LV.N..Y.LY.I.FN...TKYVTAKVS.

229E .YD...IIQ.DQ.V......**..AA.NVF**.SHT.TKYT.V...RQ..QQ.VN...KS....YG...N.T.IF.LVNA..E.L....TVLL.T.Y.DVEAWS

NL63 .YD...SIQ.DQ.V......**..AA.NAF**.S.V.NKYT.V.G.RR..QQ.IN...KS..N.YG...N.T.IF.IVN...D.LL...TVLL.TDY..VKAWS

1410 1420 1430 1440 1450 1460 1470 1480 1490 1500

SARS-CoV-2 AICHDGKA-HFPREGVF--VSNGTHWFVTQRNFYEPQIITTDNTFVSGNCDVVIGIVNNTVYDPLQPELDSFKEELDKYFKNHTS-PDVDLGDISGINAS

HKU1 GL.LS.DRGIA.KQ.Y.--IKQNDS.MF.GSSY.Y.EP.SDK.VVFMNS.S.NFTKAPFIYLNNSI.N.SD.EA.FSLW......-IAPN.TFN.H...T

OC43 GL.IA.NRGIA.KS.Y.--.NVNNT.MY.GSGY.Y.EP..EN.VV.MST.A.NYTKAPYVMLNTSI.N.PD.....GQW...Q..-VAP..SLD-Y..VT

229E GL.V..INGYVL.QPNLALYKE.NYYRI.S.IMF..R.P.IADFVQIE..N.TFVNISRSELQTIV..YIDVNKT.QELSYKLPNYTVP..VV-EQY.QT

NL63 G..V..IYGYVL.QPNLVLY.DNGVFR..S.VMFQ.RLPVLSDFVQIY..N.TFVNISRVELHTVI.DYVDVNKT.QEFAQ.LPKYVKPNFDL-TPF.LT

1510 1520 1530 1540 1550 1560 1570 1580 1590 1600

SARS-CoV-2 VVNI--------------QKEIDRLNEVAKNLNESLIDLQELGKYEQYIKWPWYIWLGFIAGLIAIVMVTIMLCCMTSCCS-C------LKGCCSCGSCC

HKU1 FLDL--------------YY.MNVIQ.SI.S..S.F.N.K.I.T..M.V........LIVILF.IFL.ILFFI..C.G.G.A.------FS---K.HN..

OC43 FLDL--------------.V.MN..Q.AI.V..H.Y.N.KDI.T..Y.V.....V..LICLAGV.MLVLLFFI..C.G.GTS.------F.---K..G..

229E IL.LTSEISTLENKSAELNYTVQK.QTLID.I.ST.V..KW.NRV.T......WV..CISVV..FV.SMLLLC..S.G..GFFSCFASSIR...E--.TK

NL63 YL.LSSELKQLEAKTASLFQTTVE.QGLIDQI.STYV..KL.NRF.N......WV..IISVVFVVLLSLLVFC.LS.G..GC.NCLTSSMR...D...TK

1610

SARS-CoV-2 KFDEDDSEPVLKGVKLHYT

HKU1 DEYGGHNDF.I.ASHDD--

OC43 DDYTGYQ.L.I.TSHDD--

229E LPYY.VEKIHIQ-------

NL63 LPYYEFEKVHVQ-------

**Membrane protein**

10 20 30 40 50 60 70 80 90 100

SARS-CoV-2 -----MADSNGTITVEELKKLLEQWNLVIGFLFLTWICLLQ**FAYANRNRF**LYIIKLIFLWLLWPVTLACFVLAAVY--RINWITGGIAIAMACLVGLMWL

HKU1 ----MNKSFFPQF.SDQAVTF.KE..FSL.VIL.FITII..**.G.TS.SM.**V.F..M.I...M..L.ITLTIFNCF.--AL.NAFLAFS.VFTIISIVI.I

OC43 MSSKPTPAPVYIW.AD.AI.F.KE..FSL.IIL.FITII..**YG.TS.SM.**V.V..M.I...M..I.IILTTFNC..--AL.NVYL.LS.VFTIVAII..I

229E -----.HHPD.AEPD--------------------------**---------**---------------RFDKPIFE.LLTPYRSLGRT.F.VL.GA.I.CWMF

NL63 -------M..SSVPLL.VYVH.RN..FSWNLILTLF.VV..**YGHYKYS.L**..GL.MSV..C...LV..LSIFDCFVNFNVD.VFF.FS.L.SIITLCL.V

110 120 130 140 150 160 170 180 190 200

SARS-CoV-2 S-YFIASFR------------LFARTRSMWSFNPETNI-LLNVPLHGTILTRPLLESELVIGAVILRGHLRIAGHHL---GR-CDIKDLPKEITVATSRT

HKU1 L-..VN.I.------------..I..G.W........N-.MCIDMK.KMFV..VI.DYHTLT.TVI....Y.Q.VK.---.TGYTLS...VYV...KVQV

OC43 V-..VN.I.------------..I..G.F........N-.MCIDMK..MYV..II.DYHTLTVT.I....Y.Q.IK.---.IGYSLA...AYMS..KVTH

229E VGILFW.IGAWPIFGFFGLDV.LIYLAFR.NYRAARAREEISISR--SA---------.H.RKYAAS.K--VTA.RFNPFWTRFKVARK.DIG-------

NL63 M-..VN...------------.WR.VKTF.A......A-IISLQVY.HNYYL.VMAAPTGVTLTL.S.V.LVD..KI---ATRVQVGQ...YVI...PS.

210 220 230 240 250

SARS-CoV-2 L-SYYKLGA**SQRVAGDSGFAAY**---SRYRIGNYKLNTDHSSSSDNIALLVQ----

HKU1 .-CT..RAF**LDKLDVN....VF**---VKSKV...R.PSS-KP.GMDT...RA----

OC43 .-CT..R.F**LD.ISDT....V.**---VKFKV...R.PSTQKG.GMDT...RNNI--

229E ---------**------IT.MSVE**SREASVAV.KFLNPD.RE.FATAFGAALAEARR

NL63 TIVCDRV.R**.VNETSQT.W.F.**---V.AKH.DFSGVASQEGVLSEREK.LHLI--

**Nucleoprotein**

SARS-CoV-2 MSDN-G--------PQNQR-NAPRITFGGPSDSTGSNQ-NGE---RSGARSKQRRPQGLPNNTASWFTALTQHGK-EDLKFPRGQGVPINTNSSPDDQIG

AGW27885.1 ..YTP.HYAGSRSSSG.RSGILKKTSWADQ.ERNYQTFNR.RKTQPKFTV.T--Q...NTIPHY...SGI..FQ.GR.F..SD......AFGVP.SEAK.

AGT51804.1 ..FTP.KQSSSRASSG.RS-VNGILKWADQ..QFRNV.TR.RRAQPKQTATS.QPSG.NVVPYY...SGI..FQ.GKEFE.AE......APGVPATEAK.

ABB90505.1 ---------------------MATVKWADA.EPQRG-----R----------------QGRIPY.LYSP.LVDS-EQPW.VIPRNL....KKD-KNKL..

AVL25612.1 ---------------------MASVNWADDRAA--------R----------------KKFPPP.FYMP.LVSSDKAPYRVIPRNL...GKGN-K.E...

110 120 130 140 150 160 170 180 190 200

SARS-CoV-2 YYRRA-TRRIRGGDGKMKDLSPRWYFYYLGTGPEAGLPYGANKDGIIWVATEGALNTPKDHIGTRNPANNAAIVLQLPQGTTLPKGFYAEGSRGGS----

AGW27885.1 .WY.HSR.SFKTA..QQ.Q.L............Y.NAS..ESLE.VF...NHQ.DTSTPSDVSS.D.TTQE..PTRF.P..I..Q.Y.V...GRSA----

AGT51804.1 .WY.HNR.SFKTA..NQRQ.L............H.KDQ..TDI..VY...SNQ.DVNTPAD.VD.D.SSDE..PTRF.P..V..Q.Y.I...GRSA----

ABB90505.1 .WNVQ--K.F.TRK..RV....KLH........HKAAKFRERVE.VV...VD..KTE.TG-Y.V.RKNSEPE.PH-F--NQK..N.VTVVEEPDSRA---

AVL25612.1 .WNVQ--E.W.MRR.QRV..P.KVH........HKD.KFRQRS..VV...K...KTVNTS-L.N.KRNQKPLEPK-F--SIA..PELSVVEFEDR.NNSS

210 220 230 240 250 260 270 280 290 300

SARS-CoV-2 QASSRSSSRSRNSSRNSTPGS-SRGTSPAR----MAGNG----GDAALALLLLDRLNQLESKMSGKG---QQQQG---------------QTVTKKSAAE

AGW27885.1 SN-..PG..--SQ..GPNTR.L..SN.NF.----HSDSIVKPDMADEI.N.V.AK.GKDS-----.----P..VT---------------KQNA.EIRHK

AGT51804.1 PN-...T..--T...A.SA..R..AN.GN.----TPTS.VTPDMADQI.S.V.AK.GKDAT----.----PK.VT---------------KHTA.EVRQK

ABB90505.1 --P...Q..--SQ..GRGE-.K.QSRN.SSDRNHNSQDDIMKAVA...KS.GF.KPQEKDK.SAKT.TPKPSRNQSPSSFQSAAKILARS.SSET.EQKH

AVL25612.1 R......T.--.N..D.SR-.T..QQ.RT.SDSNQSSSDLVAAVTL..KN.GF.NQSKSP.SS---.TSTPKKPNKPL-------------SQPRADKPS

310 320 330 340 350 360 370 380 390 400

SARS-CoV-2 ASKKPRQKRTATKA--YNVTQAFGRRGPEQTQGNFGDQELIRQGTDYKHWPQI**AQFAPSASAF**FGMSRIGMEVTP-----------SGTWLTYTGAIKLD

AGW27885.1 ILT.......PN.H--C..Q.C..K...S.---...NA.MLKL..NDPQF.IL**.EL..TPG..**.FG.KLELVKRE---SEADSPVKDVFE.R.S.S.RF.

AGT51804.1 ILN......SPN.Q--CT.Q.C..K...N.---...GG.MLKL..SDPQF.IL**.EL..T.G..**.FG..LELAKVQNLSGNPDEPQKDVYE.R.N...RF.

ABB90505.1 EMQ...W..QPNDDVTS....C..P.DLDH---...SAGVVAN.VKA.GY..F**.ELV..TA.M**LFD.H.VSKESG-----------NTVV..F.TRVTVP

AVL25612.1 QL....W..VP.RE--E..I.C..P.DFNH---.M..SD.VQN.V.A.GF..L**.ELI.NQA.L**.FD.EVSTDEVG-----------DNVQI...YKMLVA

410 420 430 440 450 460 470 480 490

SARS-CoV-2 DKDPNFKDQVI**LLNKHIDAY**KTFPPTE------PKKDKKKKADETQ--------ALPQRQKKQQTVTLLPAADLDDFSKQLQQSMSSADSTQA

AGW27885.1 STL.G.ETIMK**V.KENL...**VNSNQNTVSGSLS..PQR.RGVKQSPELFDSLNLSA----DT.HISNDFTPE.HSL-LAT.DDPYVE-..VA-

AGT51804.1 STLSG.ETIMK**V.SENLN..**QQQDG---MMNMS..PQRQRGHKNG.GENDNISV.V.KSRVQ.NKSIE.T.E.ISL-L.KMDEPYTE-.TSEI

ABB90505.1 KDH.HLGKF--**--LEELN.F**TREMQQQPLLNPSALE------FNP--SQTSPATVE.V.-DEVSIE.DII-----------DEVN--------

AVL25612.1 KDNK.LPKF--**--IEQ.S.F**TKPSSIKEMQSQSSHV------AQNTVLNASIPESK.LA-DDDSAIIEIV-----------NEVLH-------

**Replicase polyprotein 1ab**

10 20 30 40 50 60 70 80 90 100

SARS-CoV-2 ------------------------------------------------------------------MESLVPGFNEKTHVQLSLPVLQVRDVLVRGFGDS

HKU1 MIKTSKYGLGFKWAPEFRWLLPDAAEELASPMKSDEGGLCPSTGQAMESVGFVYDNHVKIDCRCILGQEWHVQS.LIRDIFVHEDLHV.EVLTKTAVKSG

OC43 MSKINKYGLELHWAPEFPWMFEDAEEKLDNPSSSEVDMICSTTAQKLETDGICPENHVMVDCRRLLKQECCVQSSLIREIVMNASPYHLEVL.QDALQSR

229E ----------------------------------------------------------------------------------------------------

NL63 ----------------------------------------------------------------------------------------------------

110 120 130 140 150 160 170 180 190 200

SARS-CoV-2 VEEVLSEARQHLKDGTCGLVEVEKGVLPQLEQPYVFI---KRSDARTAPHGHVMVELVAELEGIQYGRSGETLGVLVP**HVGEIPVAY**RKVLLRKNGNKGA

HKU1 TAILIKSPL-------HS.GGFP..YVMG.FRS.--------KTK.YVV.HLS.T--TSTTNFGEDFLGWIVPFGFM.**SYVHKWFQF**CRLYIEES-----

OC43 EAVLVTTPLGMSLEACYVRGCNP..WTMG.FRRRSVCNTGRCTVNKHVAYQLY.IDPAGVCL.AGQFMGWVIPLAFM.**VQSRKFIVP**WVMY...R.E...

229E ------------------------------------------------------------------------------**---------**-------------

NL63 ------------------------------------------------------------------------------**---------**-------------

210 220 230 240 250 260 270 280 290 300

SARS-CoV-2 GGHSYGADLKSFDLGDELGTDPYEDFQENWNTKHSSGVTRELMRELNGGAYTRYVDNNFCGPDGYPLECIK---DLLARAGKASCTLSEQLDFIDTKRGV

HKU1 ---DLIISNFK..DY.FSVE.V.AEVHAEPKG.Y.Q-KAYA.L.QYR.IKPVLF..QYG.DYS.KLAD.LQAYGHYSLQDMRQKQSVWLANCDF.IVVAW

OC43 YNKDH.CGGFG-HVY.FKVE.A.DQVHDEPKG.F.K-KAYA.I.GYR.VKPLL...QYG.DYT.SLADGLEAYA.RTLQEM..LFPTWS.ELPF.VIVAW

229E ----------------------------------------------------------------------------------------------------

NL63 ----------------------------------------------------------------------------------------------------

310 320 330 340 350 360 370 380 390 400

SARS-CoV-2 YCCREHEHEIAWYTERSEKSYELQTPFEIKLAKKFDTFNGECPNFVFPLNSIIKTIQPRVEKKKLDGFMGRIRSVYPVASP--NECNQMCLSTLMKC--D

HKU1 HVV.DSRFVMRLQ.IATICGIKYVAQPTEDVVDGDVVIREPVHLLSADAIVLKLPSLMK.MTH---MDDFS.K.I.N.DL---CD.GFVMQYGYVD.FN.

OC43 HVV.DPRYVMRLQSAATIC.VAYVANPTED.CDGSVVIKEPVHVYADDSIILRQYNLFDIMSHFYMEADTVVNAF.G..LK---D.GFVMQFGYID.EQ.

229E --------------------------------------------MACNRVTLAVASDSEISANGCSTIAQAV.RYSEA..NGFRA.RFVS-LD.QD.IVG

NL63 --------------------------------------------MFYNQVTLAVASDSEISGFGFAIPSVAV.TYSEA.AQGFQA.RFVA-FG.QD.VTG

410 420 430 440 450 460 470 480 490 500

SARS-CoV-2 HCGETSWQTGDFVKA-TCEFCGT-ENLTKEGATTCGYL---PQNAVVKIYCPACHNSEVGPEHSLAEYHNESGLKTILRKG--GRTIAFGGCV-FSYVGC

HKU1 N.DFYG.VS.NMMDGFS.PL.C.VYDSSEVK.QSS.VI---.E.P.LFTNST-----DTVNHD.FNL.G--------------YSVTP..S.-----IYW

OC43 S.DFKG.IP.NMIDGFA.TS..HVYEVGDLI.QSS.V.---.V.P.LHTKSA.-GYGGF.CKD.FTL.G--------------QTVVY....-----.YW

229E IADD.YVMG---LHGNQTL..-NIMKFSDRPFMLH.W.VFSNS.YLLEEFDVVFGKRGG.NVTYTDQ.LCGADG.PVMSEDLWQFVDH..ENEE-IIING

NL63 INDDDYVIA---LTG-.NQL.AKILPFSDRPLNLR.W.IFPNS.Y.LQDFDVVFGH-GA.SVVFVDK.MCGFDG.PV.P.NMWEFRDY.NNNTDSIVI.G

510 520 530 540 550 560 570 580 590 600

SARS-CoV-2 HNKCAYWVPRASANIGCNHTGVVGEGSEGLNDNLLEI-LQKEKVNINIVGDFKLNEEIAIILASFSASTSAFVETVKGLDYKAFKQIVESCGNFKVTKGK

HKU1 SPRPGL.I.IIKSSVK-SYDDL.YS.VV.CKSIVK.TA.ITHALYLDY.QCKCG.L.QNH..GVNNSWCRQLL-----.NRGDYNMLLKNIDL.VKRRAD

OC43 SPARNI.I.ILKSSVK-SYDSL.YT.VL.CKAIVK.TN.ICKALYLDY.QHKCG.LHQREL.GVSDVWHKQLL-----INRGVY.PLL.NIDY.NMRRA.

229E .TYVCA.LTKRKPLDYKRQNNLAI.EI.YVHGDA.HT--LRNGSVLEMAKEV.TSSKV-VLSDALDKLYKV.GSP.MTNGSNILEAFTKPVFISALVQCT

NL63 VTYQLA.DVIRKDLSYEQQNVLAI.SIHY.-GTTGHT--L.SGCKLTNAKPP.YSSKV-VLSGEWN.VYR..GSPFITNGMSLLDI..KPVFFNAFV.CN

610 620 630 640 650 660 670 680 690 700

SARS-CoV-2 AKKGAWNIGEQKSILSPLYAFASEAARVVRSIFSRTLETAQNSVRVLQKAA**ITILDGISQY**SLRLIDAMMFTSDLATNNLVVMAYITGGVVQLTSQWLTN

HKU1 FACK-FAVCGDGFVPFL.DGLIPRSYYLIQ.G-----IFFTSLMSQFSQEV**SDMCLKMCIL**FMDRVSVAT.YIEHYV.R..TQFKLL.TTL---VNKMV.

OC43 FSLETFTVCADGFMPFL.DDLVPR.YYLAV.G-----QAFCDYADK.CH.V**VSKSKELLDV**..DSLG.AIHYLNSKIVD.AQHFSDF.TSF---VSKIVH

229E CGTKS.SV.DWTGFK.SCCNVI.NKLC..PGN-----VKPGDA.ITT.Q.G**AG.KY-FCGM**T.KFVANIEGV.VWRVIA.QSVDCFVASSTFV-EEEHV.

NL63 CGSES.SV.AWDGY..SCCGTPAKKLC..PGN-----VVPGDVIITSTS.G**CGVKY-YAGL**VVKH.TNITGV.LWRVTAVHSDGMFVASSSY-DALLHR.

710 720 730 740 750 760 770 780 790 800

SARS-CoV-2 IFGTVYEKLKPVLDWLEEKFKEGVEFLRDGWEIVKFISTCACEIVGGQIVTCAK**EIKESVQTF**FKLVNKFLALCADSIIIGGAKLKALNLGETFVTHSKG

HKU1 W.N.MLDASA.ATG..LYQLLN.LFVVSQANFNFVALIPDYAK.LVNKFY.FF.**LLL.C.TVD**VLKDMPV.KTINGLVC.V.N.FYNVST.L--------

OC43 F.K.FTTSTALAFA.VLFHVLH.AYIVVESDIYFVKNIPRYASA.AQAFQSV..**VVLD.LRVT**.IDGLSCFKIGRRR.CLS.R.IYEVER.LLHS-----

229E RMD.FCFNVRNSVTDECRLAML.A.MTSNVRRQ.ASGVID---.ST.WFDVYDD**IFA..KPW.**VRKAEDIFGP.WSALASALKQ..VTTGELVRFVK.IC

NL63 SLDPFCFDVNTL.SNQLRLAFL.ASVTE.VKFAASTGVID---.SA.MFGLYDD**ILTNNKPW.**VRKASGLFDAIW.AFVAAIKLVPTTTGVLVRFVK.IA

810 820 830 840 850 860 870 880 890 900

SARS-CoV-2 LYRKCVKSREETGLLMPLKAPKEII--FLEGETLPTEVLTEEVVLKTGDLQPL-EQPTSEAVEAPLVGTPVCINGLMLLEIKDTEKYCALAP---NMMVT

HKU1 -----IPGFVLPCNAQEQQIY------.F..VAESVI.EDDVIENVKSS.SSY------.YCQP.KSVEKI..IDN.YMG-.CGD.FFPIVMNDK.ICLL

OC43 -SQLPLDVYDL.MPSQVQ..KQKP.--Y.K.SGSDFSLADSV.EVV.TS.T.C------GYS.P.K.ADKI..VDNVYMA-.AGD..YPVVVDD-HVGLL

229E NSAVA.VGGTIQI.ASVPEKFLNAFDV.VTAIQTVFDCAV.TCTIAGKAFDKVFDYVLLDNALVK..T.KLKGVRERG.NKVKYATVVVGSTEEVKSSRV

NL63 STVLT.SNGVIIMCADVPD.FQSVYRT.TQAICAAFDFSLDVFKIGDVKFKR.GDYVLT.NALVR.TTEV.RGVRDARIKKAMFT.VVVGPTTEVKFS.I

910 920 930 940 950 960 970 980 990 1000

SARS-CoV-2 NNTFTLKGGAPTKVTFGDD-TVIEVQGYKSVNI--TFELDERIDK**VLNEKCSAY**TVELGTEVNEFACVVADAVIKTLQPVSEL---LTPLGIDLDEWSMA

HKU1 DQAWRFP-C.GR..N.NEKPV.M.IPSLMT.KV--M.D..STF.D**I.GKV..EF**E..K.VT.DD.VA..C..IENA.NSCK.HPVVGYQVRAF.NKLNDN

OC43 DQAWRVP-C.GRR...KEQP..K.IISMPKIIKV-FY...NDFNT**I..TA.GVF**E.DDTVDME..YA..I..IEEK.S.CK..EGVGAKVSAF.QKLEDN

229E ERSTAVLTI.NNYSKLF.EGYTVVIGDVAYFVSDGY.R.MASPNS**..TTA--V.**KPLFAFN..-VMGTRPEKFPT.V-TCEN.ESAVLFVNDKIT.FQLD

NL63 ELATVNLRLVDCAPVVCPKGKIVVIA.QAFFYSGGFYRFMVDPTT**...DP--VF**.GD.FYTIK-.SGFKL.GFNHQFVTA.SATDAIIAVELL.LDFKT.

1010 1020 1030 1040 1050 1060 1070 1080 1090 1100

SARS-CoV-2 TY**YLFDESGEF**KLASHMYCSF-------------------------YPPDEDEEEGDCEEE-----E---F-----------------------------

HKU1 VV**.....A.DE**AM..R...T.AIEDVEDVISSEAVEDTIDGIVEDTIND...VVT..NDD.DVVTGDNDDEDVVTGDNDDEDVVTGDNDDEDVVTGDNDD

OC43 PL**F....A..E**VF.PKL..A.------------------------TA.E.D.F-------------------------L---------------------

229E --**.SI.VIDNE**IIVKPNISLC---------V--------PLYVRDYVDKWD.FCRQYSN.SWFE----------D------------------------D

NL63 VF**VYTCVVDGC**SVIVRRDAT.A---------T-------HVCFKDC.NVW.QFCIDN.G.PWFLT----------D------------------------

1110 1120 1130 1140 1150 1160 1170 1180 1190 1200

SARS-CoV-2 ----EPSTQYEYGTEDDYQGKPLEFGATSAALQPEEEQEEDWL--------------DDDSQQTVGQQD--GSEDNQTTTIQTIV------EVQPQLEME

HKU1 EDVVTGDNDD.DVVTG.NDDEDVVT.DNDDEDVVTGDNDDEDVVTGDNDDEDVVTGDN..EDVVT.DN.DEDVVTGDNDDEDVVTGDNDDED.VTGDNDD

OC43 -----EESDV.EDDVEGEETDLTITS.GQPC--VAS....S----------------SEVLED.LDDGP--SV.TSDSQVEE---------D.EMSDFVD

229E YRAFISVLDITDAAVKAAES.AFVDTIVPPCPSILKVIDGGKIWNGVIKNVNSVRDWLKSLKLNLT..G--LLGTCAKRFKRWLG------ILLEAYNAF

NL63 YNAILQ.NNPQCAIVQASES.V.LERFLPKCPEILLSIDDGH.WNLFVEKFTFVTDWLKTLKL.LTSNGL--LGNCAKRFRRVL.------KLLDVYNGF

1210 1220 1230 1240 1250 1260 1270 1280 1290 1300

SARS-CoV-2 LTPVVQTIEVNSF-----------------------------------------SG**YLKLTDNVY**IKNADIVEEAKKVKP----TVVVNAANVYLKHGGG

HKU1 EDV.TGDNDDEDVVTGDNDDEDNNDEEIVTGDNDDQIVVTGDDVDDIESIYDFDTY**KAL.VF.DV**YND.LF.SYGSS.ETETYFK.NGLWSPTITHTNCW

OC43 .ES.I.DY.NV------------C-------------------------------F**EFYT.EPEF**V------------------K.LGLYVPKATRNNCW

229E .DT..S.VKIGGL------T-----------------------------------F**KTYAF.KP.**.VIR...CKVENKTEAEWIEL-FPHNDRIKSFSTF

NL63 .ET.CSVAYTAGV-------C----------------------------I------**KYYAVNVP.**VVISGF.SRVIRRER---CDMTFPCVSCVTFFYEF

1310 1320 1330 1340 1350 1360 1370 1380 1390 1400

SARS-CoV-2 VAGALNKATNNAMQVESDDYIATNGPL-KVGGSCVLSGHNLAKHCLHVVGPNVNKGEDIQ--LLKSAYENFNQHEVLLAPLLSAGIFGADPIHSL-RVCV

HKU1 LRSV.LVMQKLPFKFKDLAIENM-WLSY...YNQSFVDYL.TTIPKAI.L.QGGFVA.FAYWF.NQFDI.AYANWCC.KCGF.FDLN.L.ALFFYGDIVS

OC43 LRSV.AVMQKLPC.FKDKNLQDL-WV.Y.QQY.QLFVDTLVN.IPANI.L.QGGYVA.FAYWF.TLCDWQCVAYWKCIKCD.ALKLK.L.AMFFYGD.VS

229E ESAYMPI.DPTHFDI.EVELLDAEFVEPGC..ILAVIDEHVFYKKDG.YY.SNGTNI-LPVAFT.A.GGKVSFSDDVEVKDIEPVYRVKLCFEFEDEKL.

NL63 LDTCFGVSKP..ID..HLELKE.VFVEP.D..QFFV..DY.WYVVDDIYY.ASCN.V-LPVAFT.L.GGKISFSDDVIVHDVEPTHKVKLIFEFEDD.VT

1410 1420 1430 1440 1450 1460 1470 1480 1490 1500

SARS-CoV-2 DTVRTNVYLAVFDKNLYD**KLVSSFLEM**KSEKQVEQKIAEIPKEEVKPFITESKPSVEQRKQDDKKIKACVEEVTTTLEETKFLTENLLLYIDINGNLHPD

HKU1 HVCKCGHNMTLIAAD.PC**T.HF.LFDD**NFCAFCTP.KIF.AACA.DVNVCH.VAVIGDEQI.G.FV------TKFSGDKFD.IVGYGMSFSMSSFE--LA

OC43 HICKCGESMVLI.VDVPF**TAHFALKDK**LFCAFITKR.VYKAACV.DVNDSH.MAV.DGKQI..HR.------TSI.SDKFD.IIGHGMSFSMTTFE--IA

229E .VCEKAIGKKIKHEGDW.**SFCKTIQSA**L.VVSCYVNLPTYYIYDEEGGNDL.L.VMISEWPLSVQQ----AQQEA..PDIAEDVVDQVEEVNSIFDIETV

NL63 SLCKKSFGKSIIYTGDWE**G.HEVLTSA**MNVIGQHI.LPQFYIYDEEGGYDV...VMISQWPISNDSNG..V.AS.DFHQLECIVDDSVREEVDIIEQPFE

1510 1520 1530 1540 1550 1560 1570 1580 1590 1600

SARS-CoV-2 SATLVSD---ID-ITFLKKDAPYIVGDVVQEGVLTAVVIPTKKAGGTTEMLAK**ALRKVPTDNY**ITTYPGQGLNGYTVEEAKTVLKKCKSAFYILPSIISN

HKU1 QLYGLCI---TPNVC.V.G.IINVARL.KADVIVNPANGHMLHG..VAKAI.V**.AG.KFSKET**AAMVKSK.VCQVGDCYVS.GG.L..TILN.VGPDARQ

OC43 QLYGSCI---TPNVC.V.G.IIKVSKL.KA.V.VNPANGHMVHG..VAKAI.V**.AGQQFVKET**TNMVKSK.VCATGDCYVS.GG.L..TVLNVVGPDART

229E DVKHDVS---PFEMP.EELNGLK.LKQLDNNCWVNS.MLQIQLT.ILDGDY.M**QFF.MGRVAK**MIERCYTAEQCIRGAMGDVG.CMYRLLKDLHTGFMVM

NL63 EVEH.LSIKQPFSFS.RDELGVRVLDQSDNNCWISTTLVQLQLTKLLDDSIEM**Q.F..GKVDS**.VQKCYELSHLISGSLGDSGKLLSELLKEKYTCS.TF

1610 1620 1630 1640 1650 1660 1670 1680 1690 1700

SARS-CoV-2 EKQEILGTVSWNLREMLAHAEETRKLMPVCVETKAIVSTIQRKYKGIKIQEGVVDYGARFYFYTSKTTVASLINTLNDLNETLVTMPLGYVTHGLNLEEA

HKU1 DGRQSYVLLARAYKHLNNYDCCLST.ISAGIFSVPADVSLTYLLGVVDK.VIL.SNNKEDFDIIQ.CQIT.VVG.K--ALAVRL.ANV.R.IKFETDAYK

OC43 QGKQSYVLLERVYKHFNNYDCVVTT.ISAGIFSVPSDVSLTYLLGTA.K.VVL.SNNQEDFDLI..CQITAVEG.K--KLAARLSFNV.RSIVYETDANK

229E DYKCSCTSGRLEESGAVLFCTP.K.AF.YGT-CLNCNAPRMCTIRQLQGTIIF.QQKPEPVNPV.-FV.KPVCSSI--FRGAVSCGHYQTNIYSQ..CVD

NL63 .MSCDC.KKFDDQVGC.FWIMPYT..FQKGECCI-CHKMQTY.LVSM.GTGVF.Q-DPAPID-IDAFP.KPICSSV--YLGVKGSGHYQTNLYSF.KAID

1710 1720 1730 1740 1750 1760 1770 1780 1790 1800

SARS-CoV-2 ARYMRSLKVPATVSVSSPDAVTAYNGYLTSSSKTPEEHFIETISLAGSYKDWSYSGQSTQLGIEFLKRGD--KSVYYTSNPTTFHLDGEVITFDNLKTLL

HKU1 LFLSGDDCFVSNS..IQEVLLLRHDIQ.NNDVRDYLLSKMTSLPK----DWRLINKFDVIN.VKTV.YFECPN.I.IC.QGKD.GYVCDGSFYKATVNQV

OC43 LILINDVAFVS.FN.LQDVLSLRHDIA.DDDAR.FVQSNVDVLPE----GWRVVNKFYQIN.VRTV.YFECTGGIDIC.QDKV.GYVQQG.FNKATVAQI

229E GFGVNKIQPWTNDALNTICIKD.DYNAKVEI.V..IKNTVD.TPKEEFVVKEKLNAFLVHDNVA.YQ-..VDTV.NGVDFDFIVNAAN.NLAHGGGLAKA

NL63 GFGVFDI.---NS..NTVCF.DVDFHSVEIEAGE-------------------VKPFAVYKNVK.YL-..ISHL.NCV.FDFVVNAAN.NLLHGGGVARA

1810 1820 1830 1840 1850 1860 1870 1880 1890 1900

SARS-CoV-2 SLREVRTIKVFTTVDNINLHTQV-VDMSMTYGQQFGPTYLDGADVTKIKPHNSHEGKTFYVLPNDDTLRVEAFEYYHTTDPSFLGRYMSALNHTKKWKYP

HKU1 CVLLAKK.D.LL...GV.FKS-ISLTVGEVF.KIL.NVFC..I....L.CSDFYAD.IL.QYE.LSLADIS.VQSSFGF.QQQ.LA.YNF.TVC-..SVV

OC43 KALFLDKVDILL...GV.FTNRF-.PVGESF.KSL.NVFC..VN...H.CDINYK..V.FQFD.LSSEDLK.VRSSFNF.QKE.LA.YNM.VNCF..QVV

229E LDVYTKGKLQRLSKEH.G.AGK.K.GTGVMVECDSLRIF-NVVGPR.G.HERDLLI.AYNTIN.EQGTPLTPILSCGIFGIK-.ETSLEV.LDVCNT.EV

NL63 IDILTEGQLQSLSK.Y.SSNGPLK.GAGVMLECEKFNVF-NVVGPRTG.HEH.LLVEAYNSILFENGIPLMPLLSCGIFGVR-IENSLK..FSCDIN.PL

1910 1920 1930 1940 1950 1960 1970 1980 1990 2000

SARS-CoV-2 QVNGLTSIKWADNNCYLATALLT---LQQ---------IELKFNPPALQDAYYRARAGEAA--NFCALIL-AYCNKTVGELGDVRET-MSYLFQHANLDS

HKU1 VNGPFF.FEQSH....VNV.C.ML---.H---------.N....KWQW.E.W.EF...RPHRL--V..V.-.KGHFKFD.PS.ATDF-IRVVLKQ.D.SG

OC43 VNGKYFTF.Q.N...FVNVSC.M---..S---------LH.T.KIVQW.E.WLEF.S.RP.R--.V..V.-.KGGFKF.DPA.S.DF-LRVV.SQVD.TG

229E K.FVY.DTEVCKVKDFVSGLVNVQKVE.PKIEPKPVSV.KVAPK.YRVDGKFSYFTEDLLCVADDKPIV.FTDSML.LDDR.LALDNAL.GVLSA.IK.C

NL63 ..FVYS.NEEQAVLKF.DGLD..PVIDD----------VDVV-K.FRVEGNFSFFDC.VN.LDGDIY.LF-TNSILMLDKQ.QLLD.KLNGIL.Q.A..Y

2010 2020 2030 2040 2050 2060 2070 2080 2090 2100

SARS-CoV-2 CKRVLNVVCKTCGQQQTTLKGVEAVMYMGTLSYEQFKKGVQIP-CTCGKQATKYLVQQESPFVMMSAPPAQYELKHGTFTCASEYTGNYQCGHYKHITSK

HKU1 AICE.ELI.D-..IK.ESRV..D...HF...AKTDLFN.YK.G-.N.A-GRIVHCTKLNV..LIC.NT.LSKD.PDD--VV.ANMFMGVGV...T.LKCG

OC43 AICDFEIA..-..VK.EQRT.LD...HF....R.DLEI.YTVD-.S..-KKLIHC.RFDV..LIC.NT.VSVK.PK.--VGSANIFIGDNV...V.VKCE

229E VDINKAIPS---.NLIKFDI.SVV.YMCVVP.EKDKHLDNNVQR..RKLNRLMCDIVCTI.ADYILPLVLSSL------..NVSFV.ELKAAEA.V..I.

NL63 LAT.KT.PA---.NLVKLVVESCTIYMCVVP.INDLSFDKNLGR.VRKLNRL.TC.IANV.AIDVLKKLLSSLTLTVK.VVE.NVMDVND.FKNDNVVL.

2110 2120 2130 2140 2150 2160 2170 2180 2190 2200

SARS-CoV-2 ETLYCIDGALLTKSSEYKGPITDVFYKEN-SYTTTIKPVTYKLDGVVCTEIDPKLDNYYKKDNSYFTEQPIDLVPNQPY--PNASFDNFKFVCDNIKF--

HKU1 SPYQHY.ACSVK.YTGVS.CL..CL.LK.LTQ.F.SMLTN.F..D.EMVAYN.D.SQ..CDNGK.Y.K-..IKAQFK.FAKVDGVYT...L.GHD.C---

OC43 QSYQLY.ASNVK.VTDVT.KLS.CL.LK.LKQ.FKSVLT..Y..D.KKI.YK.D.SQ..CDGGK.Y.Q-R.IKAQFKTFEKVDGVYT...LIGHTVC---

229E V.EDGVNVHDV.VTTDKSFEQQVGVIADKDKDLSGAV.SDLNTSELLTKA..VDWVEF.----GFKDAVTFAT.DHSAFAYES.VVNGIRVLKTSDNNCW

NL63 I.EDG.NVKDVVVE.SKSLGKQLGVVSDGVDSFEGVL.IN--T.T.LSVAPEVDWVAF.----GFEKAALFASLDVK..GY..DFVGG.RVLGTTDNNCW

2210 2220 2230 2240 2250 2260 2270 2280 2290 2300

SARS-CoV-2 ADDLNQLTGYKKPA--SRELKVTFFPDLNGD**V-VAIDYKHY**TPSFKKGAKLLHKPIVWHVNNATNKATYKPNTWCIRCLWSTKPVETSNSFDVLKSEDAQ

HKU1 -AQ..DKL.FNVDLPFVEYKVTVW-.VAT..**.-.LASDDL.**VKRYF..CETFG..VI.FCHDEASLNSLTYFNKPSF--------KSE.RYS..SVDSV-

OC43 -.S..AKWVFDSSKEFVEYKITEW-.TAT..**.-.LANDDL.**VKRYER.CITFG..VI.LSHEKASLNSLTYFNRPLLV--------DD.K.....VD.V-

229E VNAVCIALQ.S..HFI.QG.DAAWNKFVL..**.EIFVAFVY.**VARLM..D.GDAEDTLTKLSKYLANEAQVQLEHYSS.--VECDAKFK..VASIN.AIVC

NL63 VNATCIILQ.L..TFK.KG.N.LWNKFVT..**.GPFVSFIYF**ITMSS..Q.GDAEEALSKLSEYLISDSIVTLEQYST.------DICKSTVVEV..AIVC

2310 2320 2330 2340 2350 2360 2370 2380 2390 2400

SARS-CoV-2 GMDNLACEDLKPVSEEVVENPTIQKDVLECNVKTTEVVGDIILKPAN--NSLKITEEVGHTDL--MAAYVDNSSLTIKKPNELSRVLGLKTLAT---HGL

HKU1 -SEESQGNVVT..M.SQISTKEVK---.KGVR..VKIEDA..VNDE.--S.I.VVKSLSLV.V--WDM.LTGCDYVVWVA.....LVKSP.VREYIRY.I

OC43 ---DDSGDSSESGAK.TK.INI.K---.SGVK.PFK.EDSV.VNDDT--SDT.YVKSLSIV.V--YDMWLTGCKYVVRTA.A...AVNVP.IRKFIKF.M

229E ASVKRDGVQVGYCVHGIKYYSRVRS--VRGRAIIVS.EQLEPCAQSRLLSGVAY.AFS.PV.KGHYTV.DTAKKSMYDGDRFVKHD.S.LSVTSVVMV.G

NL63 ASVLKDGC.VGFCPHRHKLRSRVKF--VNGR.VI.N.GEP..SQ.SKLL.GIAY.TFS.SF.NGHYVV.DAANNAVYDGARLF.SD.STLAVTAIVVV.G

2410 2420 2430 2440 2450 2460 2470 2480 2490 2500

SARS-CoV-2 AAVNSVPWDTIANYAKPFLNKVVSTTTNIVTRCLNRVCTNYMPYFFTLLL---QLCTFTRSTNSRIKASMPTTIAKNTVKSVG-----KFCLEASFNYLK

HKU1 KPI-TI.I.LLCLRDDNQTLL.PKIFKARAIEFYGFLKWLFIYV.SL.HFTNDKTIFY.TEIA.KFTFNLFCLAL..AFQTFRWSIFI.GF.VVATVF.F

OC43 TL.-.I.I.LLNLREIKPAVN..KAVR.KTSA.F.FIKWLFVLL.GWIKISADNKVIY.TEIA.KLTCKLVALAF..AFLTFKWSMVARGACIIATIF.L

229E YVA---.VN.V---KPKPVINQLDEKAQKFFDFGDFLIH.FVIF.TW..S----MF.LCKTAVTTGDVKIMAKAPQR.GVVLKRSLKYNLKAS.AVLKS.

NL63 CVTSN..TIVS---E.ISVMDKLD.GAQKFFQFGDF.MN.IVLFLTW..S----MFSLL.TSIMKHDIKVIAKAP.R.GVILTRSFKYNIRSALFVIKQ.

2510 2520 2530 2540 2550 2560 2570 2580 2590 2600

SARS-CoV-2 SPNFSKLINIIIWFLLLSVCLGSLIYSTAALGVL**MSNLGMPSY**CTGYREGYLNSTNVTIATYCTGSIPCSVCLSGLDSLDTYPSLETIQITISS-FKWDL

HKU1 WF..LYINV.FSD.Y.PNISVFPIFVGRIVMWI-**KATF.LVTI**.DF.SKLGVGF.S----HF.N..FI.EL.H..F.M....AAIDFV.YEVDRRVLF.Y

OC43 WF..IYANV.FSD.Y.PKIGFLPTFVGKI.QWI-**KNTFSLVTI**.DL.SIQDVGFK.----Q..N...A.QF..A.F.M..N.KAIDVV.YEADRRAFV.Y

229E W----W.LAKFTKL...IYT.Y.VVL-LCVRFGP**FN--FCSET**VN..AKSNFVKDD-----..D..LG.KM..F.YQE.SQFSH.DVVWKH.TDPLFSNM

NL63 W----CV.VTLFK....LYAIYA.VF-MIVQFSP**FNS.LCGDI**VS..EKSTF.KDI-----..GN.MV.KM..FSYQEFNDLDHTSLVWKH.RDPILIS.

2610 2620 2630 2640 2650 2660 2670 2680 2690 2700

SARS-CoV-2 TAF-GLVAEWFLAYILFTRFFYVLGLAAIMQLFFSYFA--VHFISNSWLMWLIIN**LVQMAPISAM**VRMYIFFASFYYVWKSYVHVVDGCNSSTCMMCYKR

HKU1 VSLVK.IV.LVIG.S.Y.VW..P.FCLIGL...TTWLPDLFMLETMH..IRF.VF**VAN.L.AFVL**L.F..VVTAM.K.VGFIR.I.Y...KAG.LF....

OC43 .GVLKI.I.LIVS.A.Y.AW..P.FALISI.ILTTWLPELFMLSTLH.SFR.LVA**.AN.L.AHVF**M.F..II...IKLFSLFK..AY..SK.G.LF....

229E QP.IVM.LLLIFGDN-YL.C.LLYFV.QMISTVGVFLG----YKETN.FLHF.--**-----.FDVI**CDELLVTVIVIK.ISFVR..LF..ENPD.IA.S.S

NL63 QP.VI..ILLIFG-NMYL..GLLYFV.QFIST.G.FLG----.HQKQ.FLHFV--**-----.FDVL**CNEFLATFIVCKIVLFVR.IIV...NAD.VA.S.S

2710 2720 2730 2740 2750 2760 2770 2780 2790 2800

SARS-CoV-2 NRATRVECTTIVNGVRRSF**YVYANGGKGF**CKLHNWNCVNCDTFCAGSTFISDEVARDLSLQFKRPINPTDQSSYIVDSVTVKNGSIHLYFDKAGQKTYER

HKU1 .CSV..K.S...G..I.YY**DIT....T..**.VK.Q...F..HS.KP.N...TV.A.IE..KEL...V....A.H.V.TDIKQVGCMMR.FY.RD..RV.DD

OC43 ..SL..K.S...G.MI.YY**D.M....T..**.SK.Q...ID..SYKP.N...TV.A.L...KEL....Q...VAYHT.TD.KQVGC.MR.FY.RD..R..DD

229E A.LK.FPVN......Q...**..N....SK.**..K.RFF..D..SYGY.....TP..S.E.GNIT.TNVQ..GPAYVMI.K.EFE..FYR..SCETFWRYNFD

NL63 A.LK..PLQ..I..MHK..**..N....TC.**.NK..FF.....S.GP.N...NGDI..E.GNVV.TAVQ..APAYV.I.K.DFV..FYR..SGDTFWRYDFD

2810 2820 2830 2840 2850 2860 2870 2880 2890 2900

SARS-CoV-2 HSLSHFVNLDNLRANNTKGSLPINVIVFDGKSKCEE**SSAKSASVY**YSQLMCQPILLLDQALVSDVGDSAEVAVKMFDAYVNTFSSTFNVPMEKLKTLVAT

HKU1 VDA.L..DIN..LHSKV.VVPNLY.V.VE--.DADR**ANFLN.V.F**.A.SLYR....V.KK.ITTACNGIS.TQT...V..D..M.H.D.DRKSFNNF.NI

OC43 VNA.L..DYS..LHSKV.SVPNMH.V.VEN--DADK**ANFLN.A.F**.A.SLFR...MV.KN.ITTANTGTS.TET...V..D..L.M.D.DKKS.NA.I..

229E ITE.KYSCKEVFKNC.V-----LDDFIVFNNNGTNV**TQV.N....**F...L.R..K.V.SE.L.TL--.VDFNGVLHK..IDVLRNS.G---KD.NANMSL

NL63 ITE.KYSCKEV.KNC.V-----LENFIVYNN.GSNI**TQI.N.C..**F...L.E..K.VNSE.L.TL--.VDFNGVLHK...DVLCNS.F---KE.TANMSM

2910 2920 2930 2940 2950 2960 2970 2980 2990 3000

SARS-CoV-2 AEAELAKNVSLDNVLSTFISAARQGF-VDSDVETKDVVECLKLSHQSDIEVTGDSCNNYMLTYNK-VENMTPRDLGACIDCSARHINAQVAKSHNIALIW

HKU1 .H.S.REG.Q.EK..D..VGCV.KCCSI......RFITKSMISAVAAGL.F.DENY..LVP..L.-SD.IVAA...VL.QNG.K.VQGN...AA..SC..

OC43 .HSSIKQGTQIYK..D..L.C..KSCSI....D..CLADSVMSAVSAGL.L.DE....LVP..L.-SD.IVAA...VL.QN..K.VQGN...IAGVSC..

229E ..CKR.LGL.I------------------..H.---FTSAISNA.RC.VLLSDL.F..FVSS.A.PE.KLSAY..AC.MRAG.KVV..N.LTKDQTPIV.

NL63 ..CKATLGLTV------------------..DD---F.SAVANA.RY.VLLSDL.F..FFIS.A.PEDKLSVY.IAC.MRAGSKVV.HN.LIKES.PIV.

3010 3020 3030 3040 3050 3060 3070 3080 3090 3100

SARS-CoV-2 NVKDFMSLSEQLRKQIRSAAKKNNLPFKLTCATTRQVVNVVTTKIALKGGKI-VNNWL**KQLIKVTLVF**LFVAAIF**YLITPVHVM**SKHTDFSSEIIGYKAI

HKU1 FIDA.NQ.TAD.QHKLKK.CV.TG.KL...FNKQEAS.PIL..PFS....V--.LSN.**LYILFFVSLI**C.I--LL**WALL.TYSV**Y.SDIHLPAYASF.V.

OC43 S.DA.NQF.SDFQHKLKK.CC.TG.KL...YNKQMAN.S.L..PFS....A--.FSYF**VYVCF.LSLV**C.IG--L**WCLM.TYTV**H.SDFQLPVYAS..VL

229E HA...N...AEG..Y.VKTS.AKG.T.L..INENQA.TQIPA.S.VA.Q.AGDAGHS.**TW.WLLCGLV**CLIQFYL**CFFM.YFMY**--DIVS.F.GYDF.Y.

NL63 G....NT..QEGK.YLVKTT.AKG.T.L..FNDNQAITQ.PA.S.VA.Q.AG-FKRTY**NF.WY.C.FV**VAL----**-F.GVSFID**YTT.VT.FHGYDF.Y.

3110 3120 3130 3140 3150 3160 3170 3180 3190 3200

SARS-CoV-2 DGGVTRDIASTDTCFANKHADFDTWFSQRGGS--YTNDKACPLIAAVITREVGFVVPGLPGTILRTTNGDFLHFLPRVFSAVGNICYTPSKLIEYTDFAT

HKU1 .N..V...SVN.L.....FFQ..Q.YESTF..VY.H.SMD..IVV..MDEDI.STMFNV.TKV..H-GFHV....TYA.ASDSVQ....HIQ.S.N..YA

OC43 .N..I..VSVE.V.....FEQ..Q.YESTF.LSY.S.SM...IVV...DQDF.ST.FNV.TKV..Y-GYHV...ITHAL..D.VQ....HSQ.S.SN.YA

229E EN.QLKNFEAPLK.VR.VFEN.ED.HYAKF.FT-PL.KQS..IVVG.--S.IVNT.A.I.SNVYLV-GKTLIFT.QAA.GNA.V-..DIFGVTT-----P

NL63 EN.QLKVFEAPLH.VR.VFDN.NQ.HEAKF.VV-T..SDK..IVVG.--S.RIN....V.TNVYLV-GKTLVFT.QAA.GNT.V-..DFDGVTT-----S

3210 3220 3230 3240 3250 3260 3270 3280 3290 3300

SARS-CoV-2 SAC**VLAAECTIF**KDASGKPVPYCYDTNVLEGSVAYESLRPDTRYVLMD-GSIIQFPNTYLEGS-VRVVTTFDSEYCRHGTCERSEAGVCVSTSGRWVLNN

HKU1 .G.**..SSL..M.**.RGD.T.H....SDG.MKNASL.T..V.H...S.ANSNGF.R..DVIS..I-..I.R.RSMT...V.A..YA.E.I.FNFNSS.....

OC43 .G.**..SSA..M.**TM.D.S.Q....TDGLMQNAFL.S..V.HV..N.ANAKGF.R..EVLR..L-....R.RSMS...V.L..EADE.I.FNFN.S.....

229E EK.**IFTSA..RL**E-GL.GNNV...N.ALM...LP.S.IQANAY.KY-.N.NF.KL.EVIAQ.FGF.T.R.IATK...V.E.VE.N....FGFD-K.FV.D

NL63 DK.**IFNSA..RL**E-GL.GDNV...N.GLI...KP.ST.Q.NAY.KY-.AKNYVR..EILAR.FGL.TIR.LATR...V.E.RD.HK...FGFD-K.YV.D

3310 3320 3330 3340 3350 3360 3370 3380 3390 3400

SARS-CoV-2 DYYRSLPGVFCGVDAVNLLTNMFTPLIQPIGALDISASIVAGGIVAIVVTCLAYYFMRFRRAFGEYSHVVAFNTLLFLMSFTVLCLTPVYSFLPGVYSVI

HKU1 .....M..T...R.LFD.FYQF.SS..R..DFFSLT..SIF.A.L....VLVF..LIKLK....D.TS..VI.VVVWCIN.LM.FVFQ..PICAC..ACF

OC43 ...K....T...R.VFD.IYQL.KG.A..VDF.ALT..SI..A.L.VI.VLVF..LIKLK....D.TS..FV.VIVWCVN.MM.FVFQ..PT.SC..AIC

229E G--.VAN.YV..TGLW..VF.ILSMFSSSFSVAAM.GQ.LLNCALGAFAIFCCFLVTK...M..DL.VG.CTVVVAV.LNNVSYIV.QNLVTMI-A.AIL

NL63 G--.VDD.YI..DGLID..V.VLSIFSSSFSVVAM.GHMLFNFLF.AFI.F.CFLVTK.K.V..DL.YG.FTVVCAT.INNISYVV.QNLF.ML-L.AIL

3410 3420 3430 3440 3450 3460 3470 3480 3490 3500

SARS-CoV-2 YLYLTFYLTNDVSFLAHIQWMVMFTPLVPFWITIAYIICISTKHFYWFFSNYLKRRVVFNGVSFSTFEEAALCTFLLNKEMYLKLRSDVLLPLTQYNRYL

HKU1 .F.V.L.FPSEI.VIM.L..I..YGAIM...FCVT.VAMVIAN.VL.L..--YC.KIGV.VC.D.....TS.T..MIT.DS.CR.KNS.--SDVA.....

OC43 .F.A.L.FPSEI.VIM.L..L..YGTIM.L.FCLL..AVVVSN.AF.V..--YC.KLGTSVR.DG....M..T..MIT.DS.C..KNSL--SDVAF....

229E .FFA.RS.--RYAWIWCAAYLIAYISFA.W.LCAW.FLAML.GLLPSLLKLKVSTNLFEGDKFVG...S..AG..VIDMRS.E..ANSI--SPEKLKS.A

NL63 .FVF.RTV--RYAWIW..AYI.AYFL.I.W.LLTWFSFAAFLELLPNV.KLKISTQLFEGDKFIG...S..AG..V.DMRS.ER.INTI--SPEKLKN.A

3510 3520 3530 3540 3550 3560 3570 3580 3590 3600

SARS-CoV-2 ALYNKYKYFSGAMDTTSYREAACCHLAKALNDFS-NSGSDVLYQPPQTSITSAVLQSGFRKMAFPSGKVEGCMVQVTCGTTTLNGLWLDDVVYCPRHVIC

HKU1 S.....R.Y..K...AA......SQ....MET.NH.N.N.......TA.VSTSF....IV..VS.TS.I.P.I.S..Y.SM.........K.........

OC43 S.....R.Y..K...AA......SQ....MDT.TN.N.........TA.VSTSF....IV..VN.TS...P.V.S..Y.NM.........K.........

229E .S..R...Y..NANEAD..C.CYAY....ML...RDH-N.I..T..TV.YG.T-..A.L....Q...F..K.V.R.CY.N.V......G.I........A

NL63 .S......Y..SASEAD..C.CYA.....ML.YAKDH-N.M..S..TI.YN.T-....LK...Q...C..R.V.R.CY.S.V...V..G.T.T......A

3610 3620 3630 3640 3650 3660 3670 3680 3690 3700

SARS-CoV-2 TSEDMLNPNYEDLLIRKSNHNFLVQAGNVQLRVIGHSMQNCVLKLKVDTANPKTPKYKFVRIQPGQTFSVLACYNGSPSGVYQCAMRPNFTIKGSFLNGS

HKU1 S.SN.NE.D.SA..C.VTLGD.TIMS.RMS.T.VSYQ..G.Q.V.T.SLQ..Y....T.GNVK..E..T...A...R.Q.AFHVT..SSY.......C..

OC43 SAS..T..D.TN..C.VTSSD.T.LFDRLS.T.MSYQ.RG.M.V.T.TLQ.SR....T.GVVK..E..T...A...K.Q.AFHVT..SSY.......C..

229E SNTTSAI-D.DHEYSIMRL...SIIS.TAF.G.V.AT.HGVT..I..SQT.MH..RHS.RTLKS.EG.NI....D.CAQ..FGVN..T.W..R...I..A

NL63 P.TTV.I-D.DHAYSTMRL...S.SHNG.F.G.V.VT.HGS..RI..SQS.VH...HV.KTLK..DS.NI....E.IA...FGVNL.T........I..A

3710 3720 3730 3740 3750 3760 3770 3780 3790 3800

SARS-CoV-2 CGSVGFNIDYD-CVSFCYMHHMELPTGVHAGTDLEGNFYGPFVDRQTAQAAGTDTTITVNVLAWLYAAVINGDRWFLNRFTTTLNDFNLVAMKYNYEPLT

HKU1 .....YVLTG.-S.K.V...QL..S..C.T...FT......YR.A.VV.LPVK.YVQ....I......IL.NCA..VQNDVCSTE...VW..ANGFSQVK

OC43 .....YV.MG.-..K.V...QL..S..C.T...FN.D....YK.A.VV.LPIQ.YIQS..F.......IL.NCN..IQSDKCSVE...VW.LSNGFSQVK

229E ...P.Y.LKNG-E.E.V...QI..GS.S.V.SSFD.VM..G.E.QPNL.VESANQML....V.F....IL..CT.W.KGEKLFVEHY.EW.QANGFTAMN

NL63 ...P.Y.VRN.GT.E...L.QI..GS.A.V.S.FT.SV..N.D.QPSL.VESANLMLSD..V.F....LL..C..W.CSTRVNVDG..EW..ANG.TSVS

3810 3820 3830 3840 3850 3860 3870 3880 3890 3900

SARS-CoV-2 QDHVDILGPLSAQTGIAVLDMCASLKELLQNGMNGRTILGSALLEDEFTPFDVVRQCSGVTFQSAVKRTIKGTHHWLLLTILTSLLVLVQSTQWSLFFFL

HKU1 A.L--V.DA.ASM..VSIETLL.AI.R-.YM.FQ..Q....CTF...LA.S..YQ.LA..KL..KT..F..E.IY.I.ISTFLFSCIISAFVK.TI.MYI

OC43 S.L--VIDA.ASM..VSLETLL.AI.R-.K..FQ..Q.M..CSF...L..S..YQ.LA.IKL..KRT.LF...VC.IMASTFLFSCIITAFVK.TM.MYV

229E GE--.AFSI.A.K..VC.ERLLHAIQV-.N..FG.KQ...YSS.N...SINE..K.MF..NL..GKTTSMFKSIS--.FAGFFVMFWAELFV-YTTTIWV

NL63 SV--ECYSI.A.K..VS.EQLL..IQH-.HE.FG.KN...YSS.C....LAE..K.MY..NL..GKVIFGLK.MF--.FSVFFTMFWAELFI-YTNTIWI

3910 3920 3930 3940 3950 3960 3970 3980 3990 4000

SARS-CoV-2 YENAFLPFAMGIIAMSAFAMMFVKHKHAFLCLFLLPSLATVAYFNMVYMPA-SWVMRIMTWLDMVDTSLSGFKLKDCVMYASAVVLL**ILMTARTVY**DDGA

HKU1 NTHMI-GVTLCVLCFVS.M.LL.....FY.TMYII.V.C.LF.V.YLVVYKEGFRGFTYV..SYFVPAVNFTYVYEVFYGCILC.FA**.FI.MHSIN**H.IF

OC43 TT.M.S-ITFCALCVISL..LL.....LY.TMYIT.V.F.LL.N.YLVVYKHTFRGYVYA..SYYVP.VEYTYTDEVIYGMLLL.GM**VFV.L.SIN**H.LF

229E NPGFLT..MILLV.L.LCLTFV....VL..QV.....IIVA.IQ.CAWDY---H.TKVLAEKFDYNV.VMQMDIQGF.NIFICLFVA**L.H.W.FAK**ERCT

NL63 NPVILT.IFCLLLFL.LVLT..L...FL..QV....TVIAT.LY.C.LDY---YIVKFLADHFNYNV.VLQMDVQGL.NVLVCLFVV**F.H.W.FSK**ERFT

4010 4020 4030 4040 4050 4060 4070 4080 4090 4100

SARS-CoV-2 RRVWTLMNVLTLVYKVYYGNALDQAISMWALIISVTSNYSGVVTTVMFLARGIVFMCVEYCPIFFITGNTLQCIMLVYCFLGYFCTCYFGLFCLLNRYFR

HKU1 SLMFLVGRIV..ISMW.F.SN.EEDVLL--F.TAFLGT.TWTTILSLAI.K-..ANWLSVNIFY.TDVPYIKL.L.S.L.I..ILS..W.F.S...SV..

OC43 SFIMFVGRLISVFSLW.K.SN.EEE.LL--MLA.LFGT.TWTTVLS.AV.K-VIAKW.AVNVLY.TDIPQIKIVL.C.L.I..VIS..W...S.M.SL..

229E HWCTY.FSLIAVL.TAL.SYDYVSLLV.--.LCAISNEW-YIGAIIFRIC.FG.AFLPVEYVSY.DG---VKTVL.F.ML..FVSCM.Y..LYWI..FCK

NL63 HWFTYVCSLIAVA.TYF.SGDFLSLLV.--FLCAIS.DW-YIGAI.FR.S.L...FSP.SVFSV.GD---VKLTLV..LIC..LVCT.W.ILYWF..F.K

4110 4120 4130 4140 4150 4160 4170 4180 4190 4200

SARS-CoV-2 LTLGVYDYLVSTQEFRYMNSQGLLPPKNSIDAFKLNIKLLGVGGKPCIKVATVQSKMSDVKCTSVVLLSVLQQLRVESSSKLWAQCVQLHNDILLAKDTT

HKU1 MPM...N.KI.V..L....AN..R..R..FE.IL..L....I..V.V.E.SQI...LT....AN....NC..H.H.A.N....QY.SV...E..STS.LS

OC43 MP....N.KI.V..L....AN..R.....FE.LM..F....I..V.I.E.SQF...LT....AN....NC..H.H.A.N....HY.ST...E..ATS.LS

229E C......FC..PA..K..VAN..NA.NGPF..LF.SF..M.I..PRT...S.....LT.L...N...MGI.SNMNIA.N..E..Y..EM..K.N.CD.PE

NL63 C.M....FK..AA..K..VAN..HA.HGPF..LW.SF....I..DR...IS.....LT.L...N....GC.SSMNIAAN.SE..Y..D...K.N.CD.PE

4210 4220 4230 4240 4250 4260 4270 4280 4290 4300

SARS-CoV-2 EAFEKMVSLLSVLLSMQGAVD------INKLCEEMLDNRATLQAIASEFSSLPSYAAFATAQEAYEQAVANGD-SEVVLKKLKKSLNVAKSEFDRDAAMQ

HKU1 V..D.LAQ..I..FANPA...TKCLAS.DEVSDDYVQDSTV...LQ...VNMA.FVEYEV.KKNLAD.KNS.SVNQQQI.Q.E.AC.I...VYE..K.VA

OC43 V....LAQ..I..FANPA...SKCLTS.EEV.DDYAKDNTV...LQ...VNMA.FVEYEV.KKNLDE.RFS.SANQQQ..Q.E.AC.I...AYE..R.VA

229E T.Q.LLLA..AFF..KHSDFGL------GD.VDSYFE.DSI..SV..S.VGM..FV.YE..RQE..N.....S-.PQII.Q...AM....A....ESSV.

NL63 K.QGMLLA..AFF..KHSDFG------LDG.IDSYF..SS...SV..S.V.M...I.YEN.RQ...D.I...S-.SQLI.Q..RAM.I......HEISV.

4310 4320 4330 4340 4350 4360 4370 4380 4390 4400

SARS-CoV-2 RKLE**KMADQAMTQMY**KQARSEDKRAKVTSAMQTMLFTMLRKLDNDALNNIINNARDGCVPLNIIPLTTAAK**LMVVIPDYNTY**KNTCDGTTFTYASALWEI

HKU1 ....**R...L.L.N..**.E..IN..KS..V..L.....S.V.....Q...S.LD..VK.....SA..ALA.NT**.TII...KQVF**DKVV.NVYV...GSV.H.

OC43 K...**R...L.L.N..**.E..IN..KS..V..L.....S.V.....Q...S.LD..VK......A..SLA.NT**.NIIV..KSV.**DQIV.NVYV...GNV.Q.

229E K.IN**R..E..AAA..**.E..AVNRKS..V...HSL..G...R..MSSVDT.L.M..N.V...SV..A.S..R**.V..V..HDSF**VKMMVDGFVH..GVV.TL

NL63 K.IN**R..E..A....**.E...VNRKS..I...HSL..G...R..MSSVETVL.L....V...SV..A.S.S.**.TI.S..LES.**SKIVCDGSVH..GVV.TL

4410 4420 4430 4440 4450 4460 4470 4480 4490 4500

SARS-CoV-2 QQVVDADSKIVQLSEISMDNSPNLAWPLIVTALRAN--SAVKLQNNELSPVALRQMSCAAGTTQTACTDDNALAYYNTTKGGRFVLALLSDLQDLKWARF

HKU1 .T.Q...GINK..TD..V.SN----...VII.N.Y.EVANAVM.....M.HK.KIQVVNS.SDMNCNIPTQ--C...NGSS..I.Y.V...VDG..YTKI

OC43 .TIQ.S.GTNK..N...D.CN----...VII.N.Y.EV..TV......M.AK.KIQVVNS.PD..CN.PTQ--C...NSNN.KI.Y.I...VDG..YTKI

229E .E.K.N.G.N.H.KDVTKE.QEI.V....L.CE.V-----.......IM.GKMKVKATKGEGDGGITSEG.--.L..NEG.RA.MY.YVTTKPGM.YVKW

NL63 ND.K.N.GRP.HVK..TKE.VET.T....LNCE.V-----.......IM.GK.K.KPMK.EGDGGVLG.G.--.L...EG.KT.MY.YI.NKA...FVKW

4510 4520 4530 4540 4550 4560 4570 4580 4590 4600

SARS-CoV-2 PKSDGTGTIYTELEPPCRFVTDTPKGPKVKYLYFIKGLNNLNRGMVLGSLAATVRLQAGNATEVPANSTVLSFCAFAVDAAKAYKDYLASGGQPITNCVK

HKU1 M.D..-NCVVL..D...K.SIQDV..L.I........C.T.A..W.V.T.SS.I.....V...YA...SI..L...S..PK.T.L..IQQ..V..I....

OC43 L.D..-NFVVL..D...K.TVQDA..L.I.....V..C.T.A..W.V.TISS.......T...YAS..SI..L...S..PK.T.L.FIQQ..T..A....

229E EHDS.--VVTV.........I...T..QI.....V.N....R..A...YIG........KQ..FVS..HL.TH.S....P.A..L.AVKQ.AK.VG....

NL63 EYEG.CN--TI..DS....MVE..N..Q......V.N..T.R..A...FIG..I.....KQ..LAV..GL.TA...S..P.TT.LEAVKH.AK.VS..I.

4610 4620 4630 4640 4650 4660 4670 4680 4690 4700

SARS-CoV-2 MLCTHTGTGQAITVTPEANMDQESFGGASCCLYCRCHIDHPNPKGFCDLKGKYVQIPTTCANDPVGFTLKNTVCTVCGMWKGYGCSCDQLREPMLQSADA

HKU1 ...D.A...M...IK...TIN.D.Y....V.I...ARVE..DVD.I.K.R..F..V.LGI-K..ILYV.THD..Q...F.RDGS...VGSS-VAV..K.L

OC43 ...D.A...M....K.D.TTS.D.Y....V.I...ARVE..DVD.L.K.R..F..V.VGI-K...SYV.THD..R...F.RDGS...VSTD-TTV..K.T

229E ..TNGS.S.....C.IDS.TT.DTY....V.I...A.VA..TMD...QY...W..V.IGT-...IR.C.E....K...C.LNH..T..R---TAI..F.N

NL63 ..SNGA.N.....TSVD..TN.D.Y....I.....A.VP..SMD.Y.KF...C..V.IG.-L..IR.C.E.N..N...C.L.H..A..R---TTI..V.I

4710 4720 4730 4740 4750 4760 4770 4780 4790 4800

SARS-CoV-2 QSFLNRVCGVSA-ARLTPCGTGTSTD**VVYRAFDIY**NDKVAGFAKFLKTNCCRFQEKDEDDNLIDSYFVVKRHTFSNYQHEETIYNLLKDCPAVAKHDFFK

HKU1 -N.....R.T.VN...V..AS.L...**.QL.....C**.TNR..IGLYY.V......RI.D.G.KL.KF.....TNLEV.NK.K.Y.E.T.S.GV..E....T

OC43 -N.....R.A.VD...V..AS.L...**.QL......**.AS...IGLH..V......RV..NGDKL.QF.....TDLTI.NR.MKC.ERV...KF..E....T

229E -.Y....R.S..-...E..-N..DI.**YCV....V.**.KDASFIG.N..S..V..KNV.K.----.AFYI...CIK.VMD..QSM.....G.N........T

NL63 -.Y...AR.S..-...E..-N..DI.**KCV......**.KN.SFLG.C..M..V..KNA.LK----.G...I..C.K.VME..QSM....NFSG.L.E....T

4810 4820 4830 4840 4850 4860 4870 4880 4890 4900

SARS-CoV-2 FRIDGDMVPHISRQRLTKYTMADLVYALRHFDEGNCDTLKEILVTYNCCDDDYFNKKDWYDFVENPDILRVYANLGERVRQALLKTVQFCDAMRNAGIVG

HKU1 .D...SR....V.RN.S....L..C.......RND.SI.C...CE.AD.KES..S.............INI.KK..PIFNR...N..I.A.TLVEV.L..

OC43 .DVE.SR....V.KD......L..C.......RND.ML.CD..SI.AG.EQS..T.............IN..KK..PIFNR..VSATE.A.KLVEV.L..

229E WHEGRTIYGNV...D......M..CF...N...KD.EVF.....LTG..ST...EM.N.F.PI..E..H....A..KV.AN.M..C.A...E.VLK.V..

NL63 WKDGRVIYGNV..HN......M.....M.N...Q...V...V..LTG...NS..DS.G...P...E..H....S..KI.AR.M..C.AL....VAK.V..

4910 4920 4930 4940 4950 4960 4970 4980 4990 5000

SARS-CoV-2 VLTLDNQDLNGNWYDFGDFIQTTPGSGVPVVDSYYSLLMPILTLTRALTAESHVDTDLT-KPYIKWDLLKYDFTEERLKLFDRYFKYWDQTYHPNCVNCL

HKU1 .........Y.Q..........A..F..A.A.....YM..M..MCHV.DC.LF.NDS-----.RQF..VQ....DYK.E..NK.....GMK....T.D.D

OC43 ...........K......YVIAA..C..AIA.....YI..M..MCH..DC.LY.NNA-----.RLF..VQ....DYK.E..NK...H.SMP....T.D.Q

229E ............F......VLCP..M.I.YCT....YM..VMGM.NC.AS.CFMKS.IFGQDFKTF.........HKEV..NK.....G.D...D..D.H

NL63 ............F......VVSL.NM...CCT....YM...MG..NC.AS.CF.KS.IFGSDFKTF.........HKEN..NK...H.SFD.....SD.Y

5010 5020 5030 5040 5050 5060 5070 5080 5090 5100

SARS-CoV-2 DDRCILHCANFNVLFSTVFPPTSFGPLVRKIFVDGVPFVVSTGYHFRELGVVHNQDVNLHSSRLSFKELLVYAADPAMHAASGNLLLDKRTTCFSVAALT

HKU1 N....I......I...M.L.N.C......Q...........I...YK.....M.L..DT.RY...L.D..L........V..ASA...L..C......I.

OC43 .....I......I...M.L.N.C......Q...........I...YK...I.M.M..DT.RY...L.D..L......L.V..ASA.Y.L..C......I.

229E .EM.....S...T..A.TI.N.A....C..V.I....V.ATA....KQ..L.W.K...T..T..TIT...QFVT..TLIV..SPA.V....V.......S

NL63 ..M.VI......T..A.TI.G.A....C..V.I....L.TTA....KQ..L.W.K...T..V..TIT...QFVT..SLII..SPA.V.Q..I.......S

5110 5120 5130 5140 5150 5160 5170 5180 5190 5200

SARS-CoV-2 NNVAFQTVKPGNFNKDFYDFAVSKGFFKEGSSVELKHFFFAQDGNAAISDYDYYRYNLPTMCDIRQLL**FVVEVVDKY**FDCYDGGCINANQVIVNNLDKSA

HKU1 SGIK..........Q...E.VK...L.....T.D......T.......T..N..K......V..K...**..L...Y..**.EI......P.S......Y....

OC43 SG.K..........Q.....VL...LL......D......T.......T..N..K......V..K...**..L...Y..**.EI......P.S......Y....

229E TGLTS......H...E....LR.Q...D...ELT......T.K.D...K.F......R...L..G.AR**VAYQ.AAR.**....E....TSRE.V.T..N...

NL63 TGLTN.V....H..EE..N.LRLR...D...ELT........N.D..VK.F.F....K..IL..C.AR**VTYKI.SR.**..I.E....K.CE.V.T..N...

5210 5220 5230 5240 5250 5260 5270 5280 5290 5300

SARS-CoV-2 GFPFNKWGKA**RLYYDSMSY**EDQDALFAYTKRNVIPTITQMNLKYAISAKNRARTVAGVSICSTMTNRQFHQKLLKSIAATRGATVVIGTSKFYGGWHNML

HKU1 .Y....F...**....EAL.F**.E.NEIY.......L..L.......................L....G.M....C.........VP.....T......DD..

OC43 .Y....F...**....EAL.F**.E..EIY.......L..L.......................L....G.M....C.........VP.....T......DD..

229E .W.L..F...**G...E.I..**.E...I.SL....IL..M..L.......G.E.....G...LLA...T......C....V...N.......T......D...

NL63 .W.L..F...**S...E.I..**.E......L.....L..M..L.......G.E.....G...LL....T..Y...H....VN..N.......T......N...

5310 5320 5330 5340 5350 5360 5370 5380 5390 5400

SARS-CoV-2 KTVYSDVENPHLMGWDYPKCDRAMPNMLRIMASLVLARKHTTCCSLSHRFYRLANECAQVLSEMVMCGGSLYVKPGGTSSGDATTAYANSVFNICQAVTA

HKU1 RHLIK..D..V...............I...VS........EF...HGD...............I.....CY...............F.............

OC43 RRLIK..D..V...............I...VS........E....Q.D...............I.....CY...............F...........S.

229E .NLMA..DD.K..............S.I.MLSAMI.GS..V...TA.DK....S..L....T.V.YSN.GF.F.....T...............F...SS

NL63 R.LIDG....M............L...I.MISAM..GS..VN..TATD.....G..L....T.V.YSN.GF.F.....T....S......I...F...SS

5410 5420 5430 5440 5450 5460 5470 5480 5490 5500

SARS-CoV-2 NVNALLSTDGNKIADKYVRNLQHRLYECLYRNRDVDTDFVNEFYAYLRKHFSMMILSDDAVVCFNSTYASQGLVASIKNFKSVLYYQNNVFMSEAKCWTE

HKU1 ..CS.MACN.H..E.LSI....K...SNV..TDY..YT....Y.EF.C...........G...Y..D...K.YI.N.SV.QQ............S...V.

OC43 ..C..M.CN....E.LSI.A..K...SHV..SDK..ST..T.Y.EF.N...........G...Y..D...K.YI.N.SA.QQ............S...V.

229E .I.CV..VNSSNCNNFN.KK..RQ..DNC...SN..ES..DD..G..Q...........S...Y.K...GL.YI.D.SA..AT.....G....T......

NL63 .I.R...VPSDSCNNVN..D..R...DNC..LTS.EES.IDDY.G..............G...Y.KD..EL.YI.D.SA..AT..........TS...V.

5510 5520 5530 5540 5550 5560 5570 5580 5590 5600

SARS-CoV-2 TDLTKGPHEFCSQHTM**LVKQGDDYVY**LPYPDPSRILGAGCFVDDIVKTDGTLMIERFVSLAIDAYPLTKHPNQEYADVFHLYLQYIRKLHDELTGHMLDM

HKU1 N.I.N...........**...IDG....**..................LL...SV.L..............VY.E.E..QK..RV..E..K..YND.GTQI..S

OC43 H.INN...........**...MDG.D..**....N.............LL...SV.L..............VY.E.E..QK..RV..A..K..YND.GNQI..S

229E E..SI...........**QIVDENGKY.**.........IS..V.....T...AVILL..Y..........S...KP..RK..YAL.DWVKH.NKT.NEGV.ES

NL63 E...............**QIVDK.GTY.**..........S..V....V....AVVLL..Y..........S....S..RK..YVL.DWVKH.NKN.NEGV.ES

5610 5620 5630 5640 5650 5660 5670 5680 5690 5700

SARS-CoV-2 YSVMLTNDNTSRYWEPEFYEAMYTPHTVLQAVGACVLCNSQTSLRCGACIRRPFLCCKCCYDHVISTSHKLVLSVNPYVCNAPGCDVTDVT**QLYLGGMSY**

HKU1 ...I.STCDGLKFT.ES..KN..LKSA.M.S.....V.S........S...K.L..........MA.N..Y....S.......N...S...**K........**

OC43 ...I.STCDGQKFTDES..KN..LRSA.M.S.....V.S........S...K.L..........MA.D..Y....S...........N...**K........**

229E F..T.LDEHE.KF.DES..AS..EKS.....A.L..V.G...V....D.L...M..T..A....FG.D..FI.AIT.....TS..N.N...**K.....LN.**

NL63 F..T.LDNQEDKF.CED..AS..ENS.I...A.L..V.G...V....D.L.K.M..T..A....FG.D..FI.AIT......S..G.S..K**K.....LN.**

5710 5720 5730 5740 5750 5760 5770 5780 5790 5800

SARS-CoV-2 **Y**CKSHKPPISFPLCANGQVFGLYKNTCVGSDNVTDFNAIATCDWTNAGDYILANTCTERLKLFAAETLKATEETFKLSYGIATVREVLSDRELHLSWEVG

HKU1  **.**.EN...HY..K.VM..M......QS.T..PYID...K..S.K..EVD..V...E.I..........Q.....A..Q..AS..IQ.IV....VI.C..T.

OC43  **.**.ED...QY..K.VM..L......QS.T..PYID...R..S.K..DVD......E............Q.....A..Q..AS..IQ.IV.E...I....I.

229E  **.**.VD...HL.....SA.N......SSAL..MDIDV..KLS.S..SDIR..K...DAK.S.R......V..K..SV.S..AY..LK.IVGPK..L.L..S.

NL63  **.**.TN...QL.....SA.NI......SAT..LD.EV..RL..S...DVR..K...DVKDT.R......I..K..SV.S..AF..LK..VGPK..L....S.

5810 5820 5830 5840 5850 5860 5870 5880 5890 5900

SARS-CoV-2 KPRPPLNRNYVFTGYRVTKNSKVQIGEYTFEKGDYG-DAVVYRGTTTYKLNVGDYFVLTSHTVMPLSAPTLVPQEHYVRITGLYPTLNISDEFSSNVANY

HKU1 .VK....K.......HF.STG.TVL...V.D.SEL-TNG.Y..A......SI..V......S.AS..........N.AS.R-FSSVYSVPLV.QN.....

OC43 .VK....K.......HF...G.TVL...V.D.SEL-TNG.Y..A......S...V......S.AN..........N.SS.R-FASVYSVLET.QN..V..

229E .AK......S...CFQI..D..F.V..FV...V...S.T.T.KS.A.T..VP.ML.I....N.A..R...MAN..K.ST.YK.H.SF.V..AYANL.PY.

NL63 .VK......S...CFQIS.D..F....FI...VE..S.T.T.KS.V.T..VP.MI......N.Q..R...IAN..K.SS.YK.H.AF.V..AYANL.PY.

5910 5920 5930 5940 5950 5960 5970 5980 5990 6000

SARS-CoV-2 QKVGMQKYSTLQGPPGTGKSHFAIGLALYYPSARIVYTACSHAAVDALCEKALKYLPIDKCSRIIPARARVECFDKFKVNSTLEQYVFCTVNALPETTAD

HKU1 .HI..KR.C.V..........L.....V..YT..V....A............Y.F.N.ND.T.....KV..D.Y....I.D.TCK...T.I.....LVT.

OC43 .HI..KR.C.V..........L.....VF.CT..V....A............Y.F.N.ND.T..V..KV....Y....I.D.TRK...T.I.....MVT.

229E .LI.K.RIT.I.....S....CS..IGV...G....F.........S..A..VTAYSV...T...........YSG..P.NNSA....S.......VN..

NL63 .LI.K..IT.I.....S....CS...G....G....FV..A.....S..A..MTVYS....T...........YSG..P.N.SA..I.S.......CN..

6010 6020 6030 6040 6050 6060 6070 6080 6090 6100

SARS-CoV-2 IVVFDEISMATNYDLSVVNARLRAKHYVYIGDPAQLPAPRTLLTKGTLEPEYFNSVCRLMKTIGPDMFLGTCRRCPAEIVDTVSALVYDNKLKAHKDKSA

HKU1 ...V..V..L...E...I...IK.................V..S..S...RH...ITKI.CCL...I...N.Y...K...E.............KN.N.S

OC43 ...V..V..L...E...I...I..................V..S......K...T.TK..CCL...I.....Y...K...........E.....KNES.S

229E ...V..V..C.......I.Q.ISY..I..V...Q......V.IS..VM..IDY.V.TQR.CA....V..HK.Y.......N...E...E..FVPV.EA.K

NL63 ...V..V..C.......I.Q..SY..I..V...Q......VMI...VM..VDY.V.TQR.CA....V..HK.Y.......N...E...E..FVPV.PA.K

6110 6120 6130 6140 6150 6160 6170 6180 6190 6200

SARS-CoV-2 QCFKMFYKGVITHDVSSAIN**RPQIGVVREF**LTRNPAWRKAVFISPYNSQNAVASKILGLPTQTVDSSQGSEYDYVIFTQTTETAHSCNVNRFNVAITRAK

HKU1 L...VYF..QT..ES...V.**IQ..YLISK.**.KA..V.NS...........Y..KRV..VQ......A.........YS..A.....V.............

OC43 L...VY....T..ES...V.**MQ..YLINK.**.KA..L.H............FA.KRV...Q......A.........YS..A.....V.............

229E ....I.ER.SVQV.NG.S..**.R.LD..KR.**IHK.ST.S............Y..ARL...Q......A..........A..SD...A..A...........

NL63 ....V.F..NVQV.NG.S..**.K.LEI.KL.**.VK..S.S............Y...RF...QI...............YA..SD...A..............

6210 6220 6230 6240 6250 6260 6270 6280 6290 6300

SARS-CoV-2 VGILCIMSDRDLYDKLQFTSLEIPR-RNVATLQAENVTGLFKDCSKVITGLHPTQAPTHLSVDTKFKTEG-LCVDIPGIPKDMTYRRLISMMGFKMNYQV

HKU1 K..F.V..NMQ.FES.N.IT.PLDKIQ.QTLPRLHCT.N.......SCL.Y..AH..SF.A..D.Y.VNEN.A.NLNICEPVL..S....L....LDLTL

OC43 K....V..NMQ.FEA....T.TLDKVPQAVETKVQCS.N.......SYS.Y..AH..SF.A..D.Y.AT.D.A.CLGIGDSAV..S....L....LDVTL

229E K..F......T.F.A.K.FEITM------TD..S.SSC......ARNPID.P.SH.T.Y..LSDR...S.D.A.Q.G-NNNVC..EHV..Y...RFDVSM

NL63 K..F.V.C.KT.F.S.K.FEIK------H.D.HSSQ.C....N.TRTPLN.P..H.H.F..LSDQ...T.D.A.Q.G-SNNVC..EHV..F...RFDISI

6310 6320 6330 6340 6350 6360 6370 6380 6390 6400

SARS-CoV-2 NGYPNMFITREEAIRHVRAWIGFDVEGCHATREAVGTNLPLQLGFSTGVNLVAVPTGYVDTPNNTDFSRVSAKPPPGDQFKHLIPLMYKGLPWNVVRIKI

HKU1 D..SKL...KD...KR..G.V......A.....NI...F...I......DF.VEA..LFAERDCYT.KKTV..A...EK........S..QK.DI...R.

OC43 D..CKL...K...VKR....V...A..A...LDSI...F.........IDF.VEA..LFADRDGYS.KKAV..A...E.........TR.HR.D...PR.

229E P.SHSL.C..DF.M....G.L.M....A.V.GDN....V...V...N..DF..Q.E.C.L.NTGSVVKP.R.RA...E..T.IV..LR..Q..S.L.KR.

NL63 P.SHSL.C..DF...N..G.L.M...SA.VCGDNI...V...V...N...F.VQTE.C.S.NFGDVIKP.C..S...E..R.....LR..Q..LI..RR.

6410 6420 6430 6440 6450 6460 6470 6480 6490 6500

SARS-CoV-2 VQMLSDTLKNLSDRVVFVLWAHGFELTSMKYFVKIGPERTCCLCDRRATCFSTASDTYACWHHSIGFDYVYNPFMIDVQQWGFTGNLQSNHDLYCQVHGN

HKU1 ......Y.LD...S...IT.SAS....CLR..A.L.R.LN.NV.SN....YNSRTGY.G..R..YTC......LIV.I....Y..S.T....II.N..KG

OC43 ...FA.H.ID...C..L.T..AN....CLR..A.V.R.IS.NV.TK...VYNSRTGY.G..R..VTC..L...LIV.I....YI.S.S.......S..KG

229E ...IA.F.AGS..VL......G.L...T.R......AVKH.Q-.GTV...YNSV.ND.C.FK.AL.C......YV..I....YV.S.ST..HAI.N..R.

NL63 ...I..Y.S....IL......GSL...T.R.......IKY.Y-.GNS...YNSV.NE.C.FK.AL.C......YAF.I....YV.S.SQ..HTF.NI.R.

6510 6520 6530 6540 6550 6560 6570 6580 6590 6600

SARS-CoV-2 AHVASCDAIMTRCLAVHECFVKRVDWTIEYPIIGDELKINAACRKVQHMVVKAALLADKFPVLHDIGNPKAIKCVPQADVEWKFYDAQPCSDKAY**KIEEL**

HKU1 .....A.........IYD..C.S.N.NL.....SN.VS..TS..LL.RVML...M.CNRYNLCY......GLA..K--.Y.F.....F.VAK---**FVKQ.**

OC43 .....S..........YD..CNNIN.NV.....SN..S..TS..VL.RVIL...M.CNRYTLCY......G.A..K--.FDF.......IVK---**SVKT.**

229E E....G..........YD....N...S.T..M.AN.NA..KGG.T..SHIMR..IKLYNPKAI.......G.R.AVT-.AK.YC..KN.INS---**NVKT.**

NL63 E.D..G..V........D....N....VT..F.AN.KF..GCG.N..GH..R...KLY.PS.I.......GVR.AVT-.AK.YC..K..VNS---**NVKL.**

6610 6620 6630 6640 6650 6660 6670 6680 6690 6700

SARS-CoV-2 **FYSY**ATHSDKFTDGVCLFWNCNVDRYPANSIVCRFDTRVLSNLNLPGCDGGSLYVNKHAFHTPAFDKSAFVNLKQLPFFYYSDSPCESHGKQVVSDIDYV

HKU1 **..V.**DV.K.N.K..L.M.......K..S............NK......N.............NP.TRTV.E...PM.......T..VYVDGLESKQV...

OC43 **L..F**EA.K.S.K..L.M.......K..P.AV.........N.......N.............KP.ARA..EH..PM.......T..VYMDGMDAKQV...

229E **E.D.**M..GQ--M..L.........M..EF.........TR.T...E.VN.......N.......Y..R.MAK..PA.....D.GS..VVHD----QVN..

NL63 **D.D.**...GQ--L..L.........M..EF.........TR.VF..E.VN...............Y..R...K..PM....FD..D.DVVQE----QVN..

6710 6720 6730 6740 6750 6760 6770 6780 6790 6800

SARS-CoV-2 PLKSATCITRCNLGGAVCRHHANEYRLYLDAYNMMISAGFSLWVY**KQFDTYNLW**NTF--TRLQSLENVAFNVVNKGHFDGQQGEVPVSIINNTVYTKVDG

HKU1 ..R...............SK..E..CN..ES..IVTT...TF...**.N..F....**...--.T.......IY.L..V..Y..RT..L.CA...DK.VV.INN

OC43 ..................LK..E...E..ES..TATT...TF...**.T..F....**...--.K.......VY.L.KT..YT..A..M.CA...DK.V..I.K

229E ..RATN...K..I.....SK...L..A.VES..IFTQ...NI..P**TT..C....**Q..TEVN..G...I........S.V.AD..L..A.SGDK.FVRDGN

NL63 ..RASS.V....I.....SK...L.QK.VE...TFTQ...NI..P**HS..V....**QI.IE.N......I.....K..C.T.VD..L..AVV.DK.FVRYGD

6810 6820 6830 6840 6850 6860 6870 6880 6890 6900

SARS-CoV-2 VDVELFENKTTLPVNVAFELWAKRNIKPVPEVKILNNLGVDIAANTVIWDYKRDAPAHISTIGVCSMTDIAKKPTE**TICAPLTVF**FDGRVDGQVDLFRNA

HKU1 ..TVI.K.N.SF.T.I.V..FT..S.RHH..L...R..NI..CWKH.L...VK.SLFCS..Y...KY..LKF----**--IEN.NIL**....DT.ALEA..K.

OC43 E..VI.I.N..Y.T...V..F...SVRHH..L.LFR..NI.VCWKH.....A.ESIFCSN.Y...MY..LK-----**-FIDK.N.L**....DN.AFEA.KRS

229E T.NLV.V...S..T.I....F...KVGLT.PLS..K....VATYKF.L...EAER.LTSF.KS..GY..-------**-FAEDVCTC**Y.NSIQ.SYER.TLS

NL63 ..NLV.T......T......F...KMGLT.PLS..K....VATYKF.L...EAER.FTSY.KS..KY..-------**-FNEDVC.C**..NSIQ.SYER.TLT

6910 6920 6930 6940 6950 6960 6970 6980 6990 7000

SARS-CoV-2 RNGVLITEGSVKGLQPSVGPKQASLNGVTLIGEA---VKTQFNYYKKVDG**VVQQLPE--------------------------------TY**FTQSRNLQE

HKU1 ....F.STEKLSR.SMIK..QR.D....IVDKVGELK.EFW.AMR.DG.D**.IFSRTDSLCSSHYWSPQGNLGGN-CAGNVIGNDALTRF.I**.....V.SS

OC43 N...Y.STTK..S.SMIR..PR.E....VVDKVGDTDCVFY.AVR.EGQD**.IFSQFDSLGVSSNQSPQGNLGSNGKPGNVGGNDALSIS.I**.....VISS

229E T.A..FSATA..TGGK.L--PAIK..FGM.N.N.IAT..SEDGNI.NINW**F.YVRKDGKPVDHYDG------------------------F**Y..G....D

NL63 T.A..FSTVVI.N.T.I------K..FGM.N.MPVSSI.GDKGVE.L.NW**YIYVRKNGQFQDHYDG------------------------F**Y..G...SD

7010 7020 7030 7040 7050 7060 7070 7080 7090 7100

SARS-CoV-2 FKPRSQMEIDFLELAMDEFIERYKLEGYAFEHIVYGDFSHSQLGGLHLLIGLAKRFKESPFELEDFIP-MDSTVKNYFITDAQTGSSKCVCSVIDLLLDD

HKU1 .E...DL.R..IDMDDNL..AK.G..D...D.....S.N.KVI.........FR.L.K.NLLIQE.LQ-Y..SIHS.....QEC....S..T........

OC43 .TC.TD..K..IA.DQ.V..QK.G..D.........N.NQKII.........YR.QQT.NLVVQE.VS-Y..SIHS.....EKS.G..S..T...I....

229E .L...T..E...NMDIGV..QK.G..DFN...V....V.KTT........SQVRLS.MGILKA.E.VAAS.I.L.CCTV.YLNDP...T..TYM......

NL63 .T...D..Y...NMD.GV..NK.G..DFN...V....V.KTT........SQFRLS.MGVLKAD..VTAS.T.LRCCTV.YLNEL...V..TYM......

7110 7120 7130 7140 7150 7160 7170 7180 7190 7200

SARS-CoV-2 FVEIIKSQDLSVVSKVVKVTIDYTEISFMLWCKDGHVETFYPKLQSSQAWQPGVAMPNLYKMQRMLLEKCDLQNYGDSATLPKGIMMNVAKYTQLCQYLN

HKU1 ..S.V..LN..C.....NINV.FKDFQ.....N.NKIM.....M.ATND.K..YS..V...YLNVP..RVS.W...KPIN..T.C...............

OC43 ..TLV..LN.NC.....N.NV.FKDFQ.....N.EK.M....R..AASD.K..YS..V...YLNSPM.RVS.W...KPV...T.C...............

229E ..SVL..L..T.....HE.I..NKPWRW......NA.A....Q...A-E.KC.YS..GI..T...C..P.N.Y...AGLK..S...F..V........F.

NL63 ..T.L..L..G.I...HE.I..NKPYRW......N.LS....Q...A-E.KC.Y...QI..L...C..P.N.Y...AGIK..S...L..V..........

7210 7220 7230 7240 7250 7260 7270 7280 7290 7300

SARS-CoV-2 TLTLAVPYNMRVIHFGAGSDKGVAPGTAVLRQWLPTGTLLVDSDLNDFVSDADSTLIGDCATVHTANKWDLIISDMYDPKTKNVTKENDSKEGFFTYICG

HKU1 .T.....V....L.L......E....S........S.SI...N...P....SLV.YF...M.LPFDCH...........L...IGDY.V..D.......H

OC43 .T.....V....L.L....E......S........A..I...N..YP....SVA.YF...I.LPFDCQ...........I...IGEY.V..D.......H

229E ST..C..H....L.L.....Y.........KR...HDAIV..N.VV.Y.....FSVT......YLED.F..L......GR..AIDG..V.........N.

NL63 ST.MC..H....L.Y............T..KR...PDAIII.N.I..Y.....FSIT......YLED.F..L......GRI.FCDG..V..D.....LN.

7310 7320 7330 7340 7350 7360 7370 7380 7390 7400

SARS-CoV-2 FIQQKLALGGSVAIKITEHSWNADLYKLMGHFAWWTAFVTNVNASSSEAFLIGCNYLGK--PREQIDGYVMHANYIFWRNTNPIQLSSYSLFDMSKFPLK

HKU1 L.RD..S...........F..........SC..F..V.C.........G....I.....--SSFE...N......L....STTWNGGA......T..S..

OC43 M.RD..............F....E......Y..F..V.C..A......G....I...C.--.KVE...N......L....STVWNGGA......A.....

229E ..CE...I...I...V..Y...KK..E.VQR.SF..M.C.S..T......VV.I....DFAQGPF...NII....V....STVMS..YN.VL.L...NC.

NL63 V.RE...I..........Y...KY..E.IQR..F..L.C.S..T.........I....DFIQGPF.A.NTV.........STIMS..YN.VL.L...EC.

7410 7420 7430 7440

SARS-CoV-2 LRGTAVMSLKEGQINDMILSLLSKGRLIIRENNRVVISSDVLVNN-

HKU1 .A....VN.RPD.L..LVY..IER.K.LV.DTRKEIFVG.S...TC

OC43 .A....IN.RAD.....VY...E..K.L..DT.KE.FVG.S...VI

229E HKA.V.VQ..DSD..E.V...VRS.K.LV.G.GKCLSF.NH..STK

NL63 HKA.V.VT..DSDV...V...IKS...LL.N.G.FGGF.NH..STK

(**a**) Alignment of the NQKLIANQF across SARS-CoV-2 variants. The yellow shade highlights the SARS-CoV-2 peptide NQKLIANQF as the reference sequence. (**b**) Alignment of proteins across SARS-CoV-2 and seasonal coronaviruses. In bold are the 45 HLA-B15:01-restricted SARS-CoV-2-derived T-cell epitopes according to the Immune Epitope Database (<https://www.iedb.org/>, accessed on Nov 11, 2022). All peptides exhibited two or more mismatches compared to the common seasonal coronaviruses (yellow), except the spike peptide NQKLIANQF and the replicase polyprotein 1ab peptide QLYLGGMSY (green).

# Supplementary Figure S9. Discovery cohort inclusion criteria

**
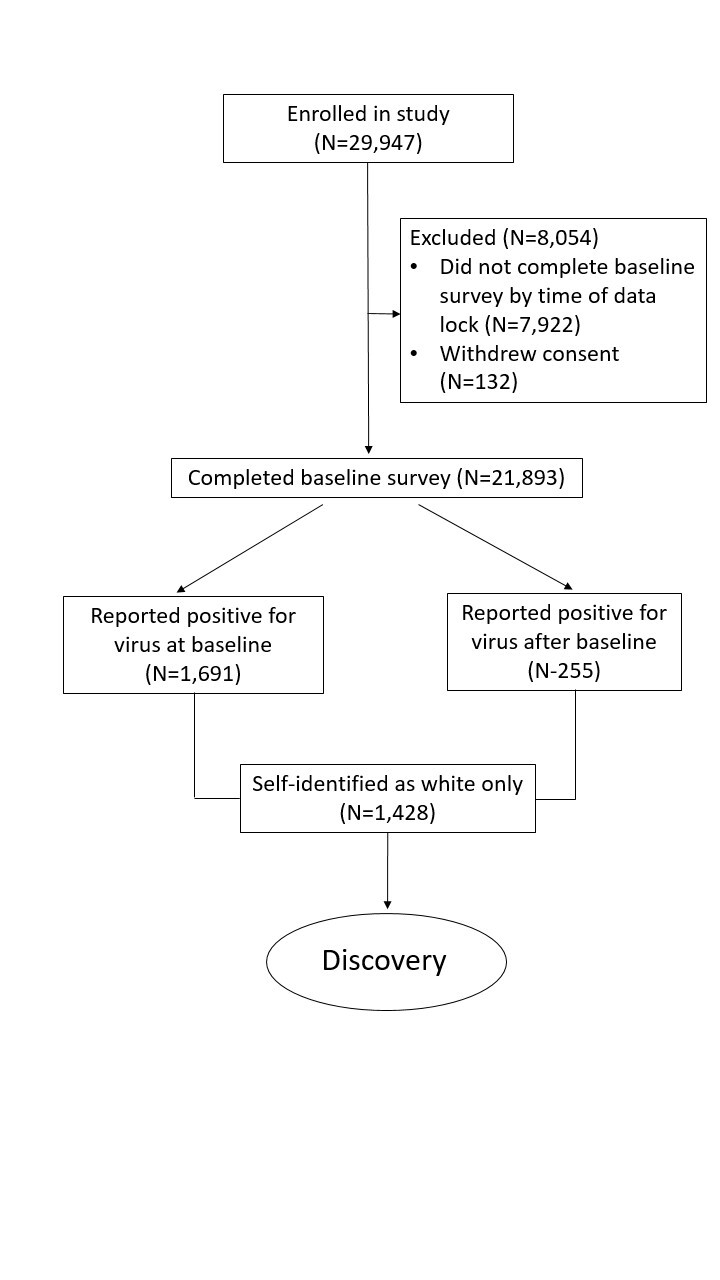
**

# Supplementary Figure S10. Definition of asymptomatic disease course in the discovery cohort

# Supplementary Figure S11. Peptide characterization


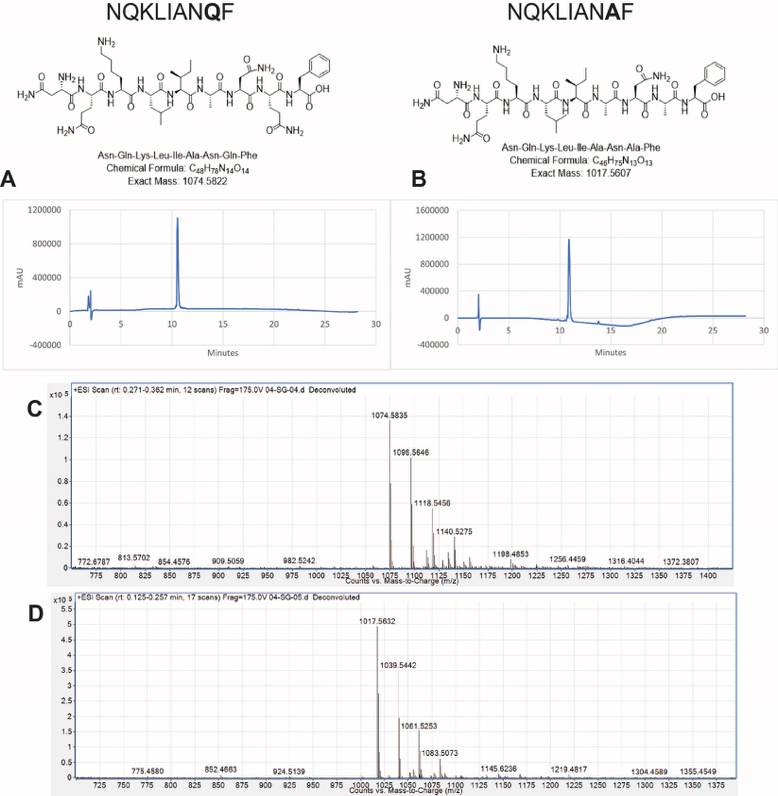

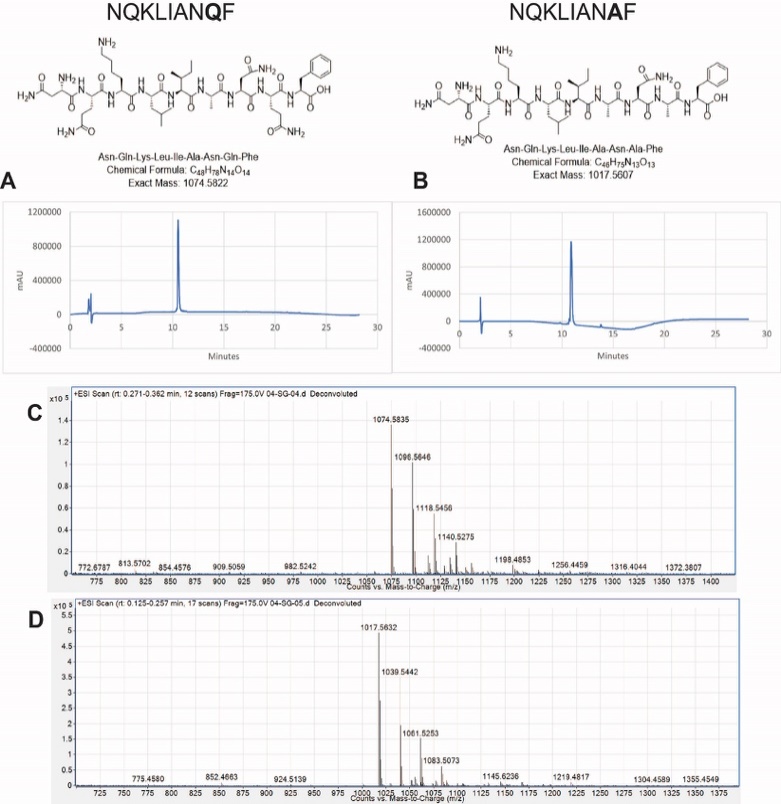

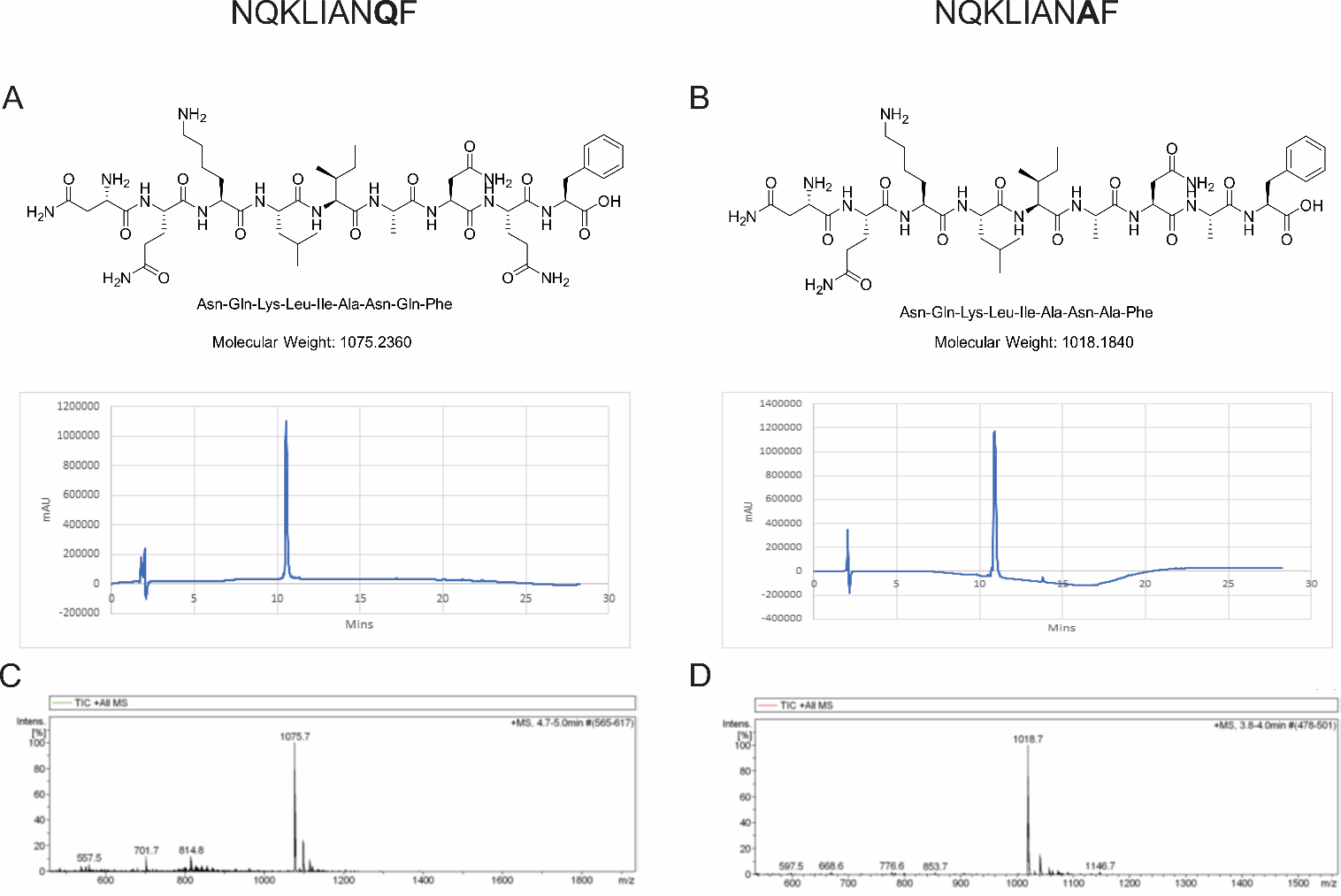


(**A**) Analytical HPLC chromatogram (254 nm) obtained for a purified sample of NQKLIAN**Q**F. The peptide elutes at 10.54 minutes (**B**) Analytical HPLC chromatogram (254 nm) obtained for a purified sample of NQKLIAN**A**F. The peptide elutes at 10.89 minutes. (**C**) Positive-ion ESI-mass spectrum for a purified sample of NQKLIAN**Q**F. [M] found=1075.7, calcd.=1075.6. (**D**) Positive-ion ESI-mass spectrum for a purified sample of NQKLIAN**A**F. [M] found=1018.7, calcd.=1018.6.

# Reference

1. Minervina, A. A. *et al.* SARS-CoV-2 antigen exposure history shapes phenotypes and specificity of memory CD8^+^ T cells. *Nat Immunol* 23, 781–790 (2022).
