# Supplementary material for: A common allele of HLA is associated with asymptomatic SARS-CoV-2 infection
Source: Nature. 2023 Jul 19;620(7972):128–36. doi: 10.1038/s41586-023-06331-x (PMC10396966; doi:10.1038/s41586-023-06331-x)
Supplement: Supplementary file 2 — Reporting Summary [file 41586_2023_6331_MOESM2_ESM.pdf]

nature portfolio

Corresponding author(s): Jill A. Hollenbach

Last updated by author(s): May 31, 2023

Reporting Summary

Nature Portfolio wishes to improve the reproducibility of the work that we publish. This form provides structure for consistency and transparency in reporting. For further information on Nature Portfolio policies, see our Editorial Policies and the Editorial Policy Checklist.

Statistics

For all statistical analyses, confirm that the following items are present in the figure legend, table legend, main text, or Methods section.

n/a	Confirmed	
☐	☒	The exact sample size (*n*) for each experimental group/condition, given as a discrete number and unit of measurement
☒	☐	A statement on whether measurements were taken from distinct samples or whether the same sample was measured repeatedly
☐	☒	The statistical test(s) used AND whether they are one- or two-sided<br>*Only common tests should be described solely by name; describe more complex techniques in the Methods section.*
☐	☒	A description of all covariates tested
☐	☒	A description of any assumptions or corrections, such as tests of normality and adjustment for multiple comparisons
☐	☒	A full description of the statistical parameters including central tendency (e.g. means) or other basic estimates (e.g. regression coefficient) AND variation (e.g. standard deviation) or associated estimates of uncertainty (e.g. confidence intervals)
☐	☒	For null hypothesis testing, the test statistic (e.g. *F*, *t*, *r*) with confidence intervals, effect sizes, degrees of freedom and *P* value noted<br>*Give P values as exact values whenever suitable.*
☒	☐	For Bayesian analysis, information on the choice of priors and Markov chain Monte Carlo settings
☒	☐	For hierarchical and complex designs, identification of the appropriate level for tests and full reporting of outcomes
☒	☐	Estimates of effect sizes (e.g. Cohen's *d*, Pearson's *r*), indicating how they were calculated

Our web collection on statistics for biologists contains articles on many of the points above.

Software and code

Policy information about availability of computer code

Data collection	Data for COVID-19 testing and symptoms in the discovery cohort was collected through a smartphone app https://eureka.app.link/covid19/. HLA genotyping from NGS data in the CHIRP/LIINC cohort was performed with commercial software packages: HLA Explorer V1.4 (Omixon, Budapest, Hungary) and AlloSeq Tx V471 (CareDx, Brisbane, USA). The crystallographic data were collected on the MX2 beamline at the Australian Synchrotron, part of ANSTO, Australia. The data were processed using XDS (version January 10, 2022) and the structures were determined by molecular replacement using the PHASER program (v2.8.3) from the CCP4 suite (v8.0.005) with a model of HLA-B*15:01 without the peptide (derived from PDB ID: 5TXS). Manual model building was conducted using COOT (v0.9.8.4) followed by refinement with BUSTER (version 1.2.14) and PHENIX (1.20.1-4487). The final models have been validated and deposited using the wwPDB OneDep System. Single-cell index sorting was performed on a BD FACSAria Fusion with BD FACSDiva Software v9.0. BD FACSymphony A3 was also used for cell acquisition with BD FACSDiva Software v9.0.
Data analysis	Initial testing for HLA associations was performed using the R package BIGDAWG V3.0.3 All additional association analysis was conducted in R using the base stats package as described in Methods, with the exception of the meta-analysis which used the R 'meta' package V 6.2-1. All molecular graphics representations were created using PyMOL V2.5. Post-acquisition analysis of T cells was performed using FlowJo software (v10).TRA and TRB sequences were analyzed with the software suite from the International ImMunoGeneTics (IMGT) Information System. The V(D)J gene nomenclature used is that of the IMGT database (www.imgt.org). Motifs enriched were identified with the MEME suite motif discovery software (v5.5.2.). TCR sequences were analyzed using FinchTV (Geospiza v1.5.0) and IMGT software. Additional data analysis for TCR was conducted using GraphPad Prism 9 (version 9.3).

For manuscripts utilizing custom algorithms or software that are central to the research but not yet described in published literature, software must be made available to editors and reviewers. We strongly encourage code deposition in a community repository (e.g. GitHub). See the Nature Portfolio guidelines for submitting code & software for further information.

Data

Policy information about availability of data

All manuscripts must include a data availability statement. This statement should provide the following information, where applicable:

- Accession codes, unique identifiers, or web links for publicly available datasets
- A description of any restrictions on data availability
- For clinical datasets or third party data, please ensure that the statement adheres to our policy

All HLA and phenotypic data for the Citizen Science and CHIRP/LIINC and UK cohorts are available at http://www.hlacovid19.org/database/ public database, Project 3 Hollenbach and Project 6 Langton. The crystallographic structures are available on the Protein Data Bank (PDB) server (https://www.rcsb.org/) under the accession codes: 8ELG and 8ELH, for HLA-B*15:01-NQK-A8 and HLA-B*15:01-Q8, respectively. PDB validation reports are attached.

Human research participants

Policy information about studies involving human research participants and Sex and Gender in Research.

Reporting on sex and gender	We clearly describe data for sex and include it as a variable in our association models.
Population characteristics	We include sex and age as covariates in our association models. Our cohort waqs 81% female with a median age of 34.In the discovery cohort, we tested models including comorbidities known to influence risk for severe disease in COVID-19, but none were associated with asymptomatic infection, our phenotype of interest, and thus are not included in the models presented in the manuscript.
Recruitment	Discovery cohorts: Subjects were volunteer bone marrow donors with valid email addresses on file with the National Marrow Donor Program (NMDP) who were invited to participate in the study through an email outreach campaign that began in July 2020. Because the donor registry has substantially greater numbers of registrants who self-identify as White compared to other population groups, we only accrued sufficient numbers of subjects in that group for a well-powered analysis. Some self-selection bias may exist both with regard to the fact that participants who tested positive for the virus may have been more likely to respond to the survey. Likewise, the requirement for a smartphone may have proved a limitation for some potential participants.CHIRP/LIINC cohort: Participants were identified through local clinical systems (UCSF Moffitt Hospital, San Francisco General Hospital, Kaiser, California Pacific Medical Center, etc.) as well as the San Francisco Department of Public Health. After confirmation of SARS CoV-2 test results or exposure to determine eligibility, participants were asked to sign a consent form, complete a baseline visit, and schedule follow-up in-person visits.
Ethics oversight	Participation in the UCSF Citizen Science study and linking to NMDP HLA data were approved by the Institutional Review Board for the University of California, San Francisco (IRB# 17-21879 and IRB# 20-30850, respectively). All participants provided written informed consent agreeing to research and publication of research results. The CHIRP and LIINC studies were approved by the Institutional Review Board for the University of California, San Francisco, (IRB# 20-30588 and 20-30479, respectively). All participants provided written informed consent agreeing to research and publication of research results. HLA-genotyped PBMCs from the USA were stored in the National Marrow Donor Program® (NMDP)/Be The Match® Research Sample Repository (ClinicalTrials.gov protocol # NCT04920474). All individuals consented to research and publication of research results and had been previously genotyped for HLA class I and class II. Ethics approval to undertake the research for the Australian PBMCs was obtained from the QIMR Berghofer Medical Research Institute Human Research Ethics Committee (P2282) and La Trobe University Human Research Ethics Committee (HEC21097).

Note that full information on the approval of the study protocol must also be provided in the manuscript.

Field-specific reporting

Please select the one below that is the best fit for your research. If you are not sure, read the appropriate sections before making your selection.

☒ Life sciences ☐ Behavioural & social sciences ☐ Ecological, evolutionary & environmental sciences

For a reference copy of the document with all sections, see nature.com/documents/nr-reporting-summary-flat.pdf

Life sciences study design

All studies must disclose on these points even when the disclosure is negative.

Sample size	Our study was designed to be prospective, sample size was based on the number of subjects reporting a positive test for SARS-CoV-2 prior to the end of April 2021.
Data exclusions	We excluded subjects who self-identified as other than 'White' within the registry database, owing to insufficient numbers in these groups.
Replication	We replicated our findings in two independent patient cohorts

Randomization	Randomization was not applicable as this was a retrospective case-control study design.
Blinding	Blinding was not relevant to this study, as this was a retrospective case-control study design.

Reporting for specific materials, systems and methods

We require information from authors about some types of materials, experimental systems and methods used in many studies. Here, indicate whether each material, system or method listed is relevant to your study. If you are not sure if a list item applies to your research, read the appropriate section before selecting a response.

Materials & experimental systems

n/a	Involved in the study
☐	☒ Antibodies
☐	☒ Eukaryotic cell lines
☒	☐ Palaeontology and archaeology
☒	☐ Animals and other organisms
☒	☐ Clinical data
☒	☐ Dual use research of concern

Methods

n/a	Involved in the study
☒	☐ ChIP-seq
☐	☒ Flow cytometry
☒	☐ MRI-based neuroimaging

Antibodies

Antibodies used

Antibody Clone Fluorophore Vendor Catalog #
CD8 SKI PE-Cy7 eBioscience 25-0087-42
CD4 RPA-T4 PerCP-Cy5.5 BioLegend 300530
CD14 HCD14 PerCP-Cy5.5 BioLegend 325622
CD16 B73.1 PerCP-Cy5.5 BioLegend 360712
CD19 HIB19 PerCP-Cy5.5 BioLegend 302230
CCR7 G043H7 Alexa 488 BioLegend 353206
CD45RA HI100 APC/Fire 750 BioLegend 304152
Viability N/A eFluor 506 Invitrogen 65-0866-14
Streptavidin N/A PE Invitrogen S866
Streptavidin N/A APC Invitrogen S868
Streptavidin N/A BV421 BioLegend 405225
Streptavidin N/A PE-CF594 BioLegend 405247
CD3 BV480 BD Biosciences 566105
CD8 PerCP-Cy5.5 BD Biosciences 565310
CD4 BV650 BD Biosciences 563875
CD107 Alexafluor488 Invitrogen 53-1079-42
IFN-γ BV421 BD Biosciences 562988
TNF PE-Cy7 BD Biosciences 557647
IL2 PE BD Biosciences 559334
MIP1β APC BD Biosciences 560686
CD14 APCH7 BD Biosciences 560180
CD19 APCH7 BD Biosciences 560727
CD45RA FITC BD Biosciences 555488
CD27 APC BD Biosciences 558664
CCR7 PE-Cy7 BD Biosciences 557648
CD95 BV421 BD Biosciences 562616
PD1 BV605 BD Biosciences 563245

Validation

All antibodies used were commercial antibodies validated by the manufacturer, as described in the links provided in the Report Summary.
https://www.thermofisher.com/antibody/product/CD8a-Antibody-clone-SK1-Monoclonal/25-0087-42
https://www.biolegend.com/fr-ch/products/percp-cyanine5-5-anti-human-cd4-antibody-4216
https://www.biolegend.com/fr-ch/products/percp-cyanine5-5-anti-human-cd14-antibody-4253
https://www.biolegend.com/fr-ch/products/percp-cyanine5-5-anti-human-cd16-antibody-9135
https://www.biolegend.com/fr-ch/products/percp-cyanine5-5-anti-human-cd19-antibody-4226
https://www.biolegend.com/fr-ch/products/alexa-fluor-488-anti-human-cd197-ccr7-antibody-7496
https://www.biolegend.com/fr-ch/products/apc-fire-750-anti-human-cd45ra-antibody-13200
https://www.bdbiosciences.com/content/bdb/paths/generate-tds-document.nz.566105.pdf
https://www.bdbiosciences.com/content/bdb/paths/generate-tds-document.ca.565310.pdf
https://www.bdbiosciences.com/content/bdb/paths/generate-tds-document.ca.563875.pdf
https://www.thermofisher.com/document-connect/document-connect.html?url=https://assets.thermofisher.com/TFS-Assets%2FLSG%2Fcertificate%2FCertificates-of-Analysis%2F53-1079-42_2076068.PDF
https://www.bdbiosciences.com/content/bdb/paths/generate-tds-document.ca.562988.pdf
https://www.bdbiosciences.com/content/bdb/paths/generate-tds-document.ca.557647.pdf
https://www.bdbiosciences.com/content/bdb/paths/generate-tds-document.ca.559334.pdf

https://www.bdbiosciences.com/content/bdb/paths/generate-tds-document.ca.560686.pdf
https://www.bdbiosciences.com/content/bdb/paths/generate-tds-document.ca.560180.pdf
https://www.bdbiosciences.com/content/bdb/paths/generate-tds-document.ca.555488.pdf
https://www.bdbiosciences.com/content/bdb/paths/generate-tds-document.ca.558664.pdf
https://www.bdbiosciences.com/content/bdb/paths/generate-tds-document.ca.557648.pdf
https://www.bdbiosciences.com/content/bdb/paths/generate-tds-document.ca.562616.pdf
https://www.bdbiosciences.com/content/bdb/paths/generate-tds-document.ca.563245.pdf

Eukaryotic cell lines

Policy information about cell lines and Sex and Gender in Research

Cell line source(s)	The CD8+ T cell lines were generated from Peripheral blood mononuclear cells (PBMCs) that were separated from whole blood or buffy coats from donors using density gradient centrifugation.
Authentication	Cell lines were not authenticated
Mycoplasma contamination	The cell lines were not tested for mycoplasma contamination
Commonly misidentified lines (See ICLAC register)	N/A

Flow Cytometry

Plots

Confirm that:

☒ The axis labels state the marker and fluorochrome used (e.g. CD4-FITC).

☒ The axis scales are clearly visible. Include numbers along axes only for bottom left plot of group (a 'group' is an analysis of identical markers).

☒ All plots are contour plots with outliers or pseudocolor plots.

☒ A numerical value for number of cells or percentage (with statistics) is provided.

Methodology

Sample preparation	A total of 20 unexposed and 1 triple vaccinated (VAC62) donors were recruited with all the details listed in Supplementary Table S19. PBMC were separated from whole blood or buffy coats using density gradient centrifugation. PBMC were used fresh or were cryogenically stored until use. HLA-genotyped PBMCs from the USA were stored in the National Marrow Donor Program® (NMDP)/Be The Match® Research Sample Repository (ClinicalTrials.gov protocol # NCT04920474) that had been collected from healthy donors prior to the start of the COVID-19 pandemic.
Instrument	BD LSR Fortessa and BD FACSymphony A3
Software	BD FACSDiva was used for sample collection. Cells were single-cell index sorted using a BD Aria Fusion with FACSDiva software.<br>FlowJo 10.7.1 and 10.8.1 were used for sample analysis.
Cell population abundance	An aliquot of PBMCs was used for cell surface staining and counting with 123count eBeads (Invitrogen). The remaining PBMCs were stained with the indicated tetramer pools and enriched using anti-PE magnetic microbeads (Miltenyi) over a magnetic column, cell-surface stained, and counted as for pre-enrichment.
Gating strategy	CD8+ T cells were identified by gating of live singlet CD8+ lymphocytes that were negative for CD4/CD14/CD16/CD19. A stringent tetramer gating strategy was employed whereby CD8+ T cells labeled with only two fluorophores were considered antigen-specific.<br>TAME:CD8+ T cells were identified by gating of live singlet CD8+ lymphocytes that were negative for CD4/CD14/CD16/CD19 . A stringent tetramer gating strategy was employed whereby CD8+ T cells labeled with only two fluorophores were considered antigen-specific. Memory status of tetramer-positive CD8+ T cells was determined by lack of CCR7 and CD45RA co-expression.

☒ Tick this box to confirm that a figure exemplifying the gating strategy is provided in the Supplementary Information.
